# Assessment of viral community functional potential from viral metagenomes may be hampered by contamination with cellular sequences

**DOI:** 10.1098/rsob.130160

**Published:** 2013-12

**Authors:** Simon Roux, Mart Krupovic, Didier Debroas, Patrick Forterre, François Enault

**Affiliations:** 1 Laboratoire ‘Microorganismes: Génome et Environnement’, Clermont Université, Université Blaise Pascal, Clermont-Ferrand, France; 2 CNRS UMR 6023, LMGE, Aubière, France; 3 Département de Microbiologie, Institut Pasteur, Unité Biologie Moléculaire du Gène chez les Extrêmophiles, Paris, France; 4 Laboratoire de Biologie Moléculaire du Gène chez les Extrêmophiles, Institut de Génétique et Microbiologie, Université Paris Sud, CNRS UMR 8621, Orsay, France

**Keywords:** phages, viruses, metagenomics, functional potential

## Abstract

Although the importance of viruses in natural ecosystems is widely acknowledged, the functional potential of viral communities is yet to be determined. Viral genomes are traditionally believed to carry only those genes that are directly pertinent to the viral life cycle, though this view was challenged by the discovery of metabolism genes in several phage genomes. Metagenomic approaches extended these analyses to a community scale, and several studies concluded that microbial and viral communities encompass similar functional potentials. However, these conclusions could originate from the presence of cellular DNA within viral metagenomes. We developed a computational method to estimate the proportion and origin of cellular sequences in a set of 67 published viromes. A quarter of the datasets were found to contain a substantial amount of sequences originating from cellular genomes. When considering only viromes with no cellular DNA detected, the functional potential of viral and microbial communities was found to be fundamentally different—a conclusion more consistent with the actual picture drawn from known viruses. Yet a significant number of cellular metabolism genes was still retrieved in these viromes, suggesting that the presence of auxiliary genes involved in various metabolic pathways within viral genomes is a general trend in the virosphere.

## 2. Introduction

Studies on the quantitative and functional importance of viruses in natural environments emerged more than 20 years ago with reports on the high concentration of bacteriophages in natural waters [1]. Viruses were progressively shown to be the most abundant biological entities in the biosphere [2] and these observations have prompted scientists to determine the roles of viruses in diverse ecosystems. Viruses are now considered an important factor in the control of microorganisms in various ecological niches [3,4], interfering with major biogeochemical cycles [2]. In addition, viruses also mediate genetic exchange among bacteria by transduction (i.e. the process by which DNA is transferred from one bacterium to another by a virus) and may have been having a great influence on the evolution of cellular organisms since the beginning of cellular life [5].

Although viruses were first believed to carry only those genes that are directly involved in viral reproduction [6], accumulation of complete viral genome sequences during the past decade revealed a deviation from this general paradigm. Besides the bona fide viral genes (i.e. for virion structure and assembly, and genome replication), several viruses were found to contain ‘auxiliary metabolism genes’. Phosphate metabolism-associated genes, for example, were described in *Roseobacter* phage SIOI [7], while several photosystem genes were discovered in cyanophages [8,9]. The discovery of such metabolism genes in several viral genomes was one of the elements fuelling the recently renewed debate about the true nature of viruses and their place among cellular life forms [10,11]. However, the precise range of metabolism-associated genes encompassed in viral genomes is still to be characterized.

Metagenomic approaches provide access to genetic material at a community scale, and seem thereby well fitted to address the question of the functional potential of environmental viral communities. Owing to the growing awareness of the key role of viruses in the biosphere, a great deal of viromes (i.e. viral metagenomes) were generated to better understand the structure and dynamics of viral communities from various biomes. Surprisingly, for most viromes, reads with detectable homologues are mostly affiliated to prokaryotic genes [12–14]. This observation is explained by the protein conservation across viral and cellular genomes, and the presence of prophage sequences within microbial genomes, these two factors being amplified by the fact that more microbial than viral sequences are available in databases [15]. Even more puzzling, functional profiles were determined to be similar for microbial and viral metagenomes [16]. Nevertheless, a reasonable doubt is associated with the fact that all cellular functions are represented in a similar proportion in microbial and viral genomes. Indeed, such a similarity could also result from the presence of cellular DNA in viromes, presence that cannot be excluded [17].

In this study, 67 published viral metagenomes from various biomes were analysed to identify and quantify the extent and possible origins of bacterial-like sequences in viromes. After identification of datasets that correspond to viromes *sensu stricto* (i.e. sequence datasets exclusively from the viral community), a more accurate picture of the prevalence of diverse metabolism genes encoded by viruses could be drawn, providing a first unbiased view of the functional potential of viral communities across various biomes.

## 3. Material and methods

### 3.1. Genomic and metagenomic sequence data

The prokaryotic sequences used as references (1312 complete genomes and the corresponding 4 457 923 protein sequences) originated from KEGG database [18]. Viral genomes (2852) and the encoded protein sequences (104 703) were obtained from RefSeqVirus database [19]. Reference databases were downloaded in June 2011 and March 2012, respectively. The metagenomic data were composed of 45 microbial and 67 viral publicly available metagenomes [14,16,20–22] (see electronic supplementary material, table S1).

### 3.2. Detection of ribosomal DNA in viromes

Genes encoding the 16S and 23S rRNAs (from prokaryotic genomes) were identified in viromes using rna_hmm, a sensitive tool based on HMM search [23]. Ribosomal DNA (rDNA) gene prediction was then checked through a Blast comparison with the Silva database [24].

### 3.3. Detection of prophage-like regions in prokaryotic genomes

Prokaryote sequences similar to viral sequences, referred to as viral-like-genes, were identified by Blastp comparison [25] according to bit-score and *E*-value thresholds of 50 and 0.001, respectively. Prophage-like regions were then defined according to the following criteria: a region of four or more genes, containing at least one viral-like gene, and composed of only viral-like genes or hypothetical protein-coding genes (i.e. bacterial genes for which no function are identified, noted by the keywords ‘hypothetical protein’ or ‘putative protein’ in their annotation). Although several more sophisticated prophage detection tools are available [26,27], we intentionally relied on such ‘naive’ prophage definition criteria in order to detect not only functional prophages but also the defective and degenerated ones.

### 3.4. Comparison of viromes and microbiomes to prokaryotic genomes

To avoid bias resulting from differences in the length of metagenomic sequences (see electronic supplementary material, table S1), all virome reads were randomly truncated to 100 bp before proceeding to comparison. Viral genomes from RefSeqVirus were also truncated to 100 bp and used as a simulated metagenome (100 000 sequences of 100 bp generated with Grinder [28]). All resulting 100 bp reads were compared with prokaryotic genomes using tBlastx (bit-score and *E*-value thresholds of 50 and 0.001, respectively). Each read was affiliated to its best-matched prokaryotic genome so that, for each metagenome, the microbial hit ratio (MHR) was determined as
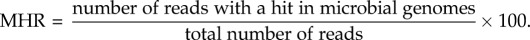


According to the prophage-like regions formerly identified, the prophage hit ratio (PHR) was determined as



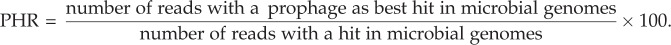


**Figure 1.**
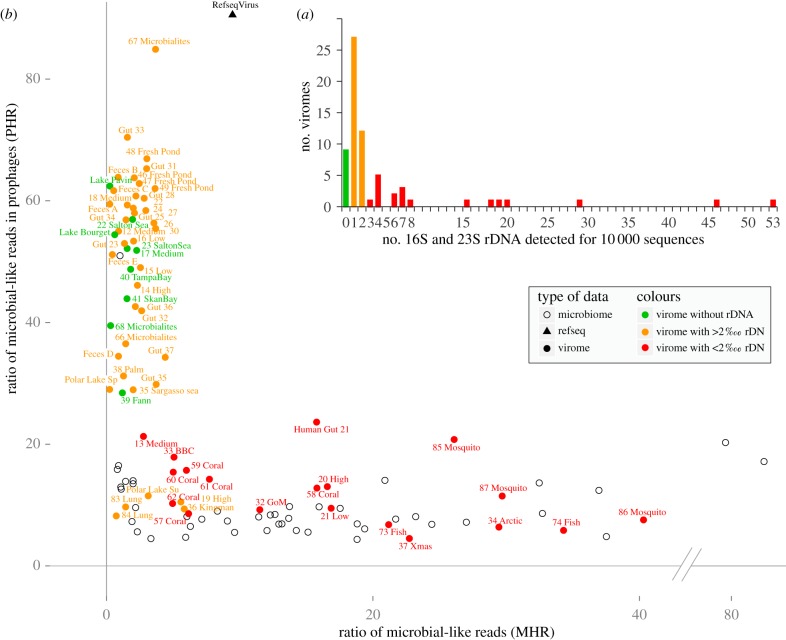
(*a*) Distribution of relative number of rDNA genes detected in viromes. The three defined categories are coloured green for virome free from cellular DNA, orange for a low level of cellular DNA and red for a high level of cellular DNA. (*b*) PHR/MHR plot for each metagenome, either viral (filled dots) or microbial (black circles). For each dataset, the MHR represents the proportion of reads having a significant similarity in a prokaryote genome. For reads having a hit in a bacterial genome, the PHR represents the proportion of these microbial reads that are found in a prophage-like region. Viromes are coloured according to their number of rDNA genes detected.

These two ratios are summarized on the PHR versus MHR plot (figure 1*b*).

### 3.5. Identification of the origin of cellular DNA in viromes

To ensure that a low PHR did not result from affiliation of reads to specific genomic regions, such as unknown viral genes or isolated genes common to prokaryotes and viruses, a complementary procedure was performed. For each virome, recruitment plots were generated for each genome recruiting 500 or more reads. Plots were manually inspected when the PHR of a virome–genome pair was lower than the prophage ratio (±5%) of the genome determined from the prophage detection step (i.e. in cases where the virome reads did not seem to be specifically associated with prophage regions, but rather equally distributed along the genome). This detailed analysis of virome–genome pairs enabled us to identify the genome(s) involved for each virome in which cellular DNA was detected. All recruitment plots are available on a dedicated web page: http://metavir-meb.univ-bpclermont.fr/Recruitment_plots/recruitment_plot_gallery.php.

### 3.6. Detection of gene transfer agent gene clusters in microbial genomes

To determine the possible presence of cellular DNA in viromes owing to gene transfer agents (GTAs), four previously described GTA gene clusters (see electronic supplementary material, table S4) were used to detect potential homologous clusters in prokaryotic genomes using Blastp (bit-score and *E*-value thresholds of 50 and 0.001, respectively). Three of these clusters are well documented and represent experimentally confirmed GTA gene clusters [29]: one in the Spirochaetes *Brachyspira hyodysenteriae* [30], and two in the α-proteobacteria *Rhodobacter capsulatus* [31] and *Silicibacter pomeroyi* [32]*.* The fourth cluster used is a predicted GTA-encoding genomic region from *Methanococcus voltae* A3 [33], a methanogenic, anaerobic archaeon previously demonstrated to produce GTA particles [34]. Genomic regions enriched in GTA-like genes were manually inspected and GTA clusters in the reference set of prokaryotic genomes were predicted according to the following conditions: the absence of gene coding for an integrase, the size of the genomic region considered (less than 40 genes) and the genomic neighbourhood of the putative cluster. According to the identification of cellular DNA in viromes and of genomes containing GTA gene clusters, the ratio of GTA-containing genome was calculated for each virome.

### 3.7. Functional analysis of viromes and microbiomes

Functional profiles of 42 viromes and 45 microbiomes, previously analysed by Dinsdale *et al*. [16] and Kristensen *et al.* [17] (see electronic supplementary material, table S1), were downloaded from the Mg-Rast web-server [35] and compared. Three comparisons of profiles were performed: all viromes versus all microbiomes, viromes with clearly identified microbial-originating sequences (‘red’ viromes) versus all microbiomes, and viromes considered as mostly composed of viral sequences (‘green’ and ‘orange’ viromes) versus all microbiomes. Plots were generated for each combination and Pearson's correlation coefficients were computed.

Functional annotation of the nine viral-only viromes was performed using tBlastx comparison between viromes and the KEGG database, the KEGG Orthology (KO) system and the associated online pathway representation [36].

## 4. Results and discussion

### 4.1. Evidence for the presence of cellular DNA in some viromes

The detection of typical prokaryotic genes never retrieved in a viral genome, such as those coding for ribosomal RNA (rDNA), indicates that a virome most probably contains DNA from cellular origin. The ratio of rDNA genes was determined in the 67 public viromes analysed in this study (see electronic supplementary material, table S1). According to this ratio, ranging from 0 to 5.3‰, viromes were separated into three groups (figure 1*a*; electronic supplementary material, table S2):

— viromes with no rDNA genes that can be considered as devoid of cellular sequences;
— viromes with a rDNA ratio lower than 0.2‰ (2 from 10 000 sequences), for which the amount of cellular sequences can be considered as very low and likely to be negligible; and
— viromes with a rDNA ratio higher than 0.2‰ (up to 5.3‰), similar in average to the rDNA ratios observed in microbiomes that can be considered as containing a non-negligible proportion of cellular sequences.

A gradient of presence of rDNA genes is therefore observed and is highly dependent on the investigated ecosystem. For example, all of the human gut viromes were found to contain rDNA sequences and only viromes from aquatic systems were rDNA-free. This first observation illustrated that it might prove difficult to purify viral capsids from complex matrices (e.g. faeces, gut, coral samples, etc.), but also prompted us to determine the extent of cellular DNA in viromes beyond rDNA genes.

### 4.2. What is the extent of microbial DNA in viromes?

We complemented the detection of rDNA genes by determining the ratio of virome reads with a hit against a microbial genome (MHR). MHRs exhibited a great variability, ranging from 0.2 to 40.3% (6.2% on average; electronic supplementary material, table S2). Moreover, viromes with a high MHR (more than 10%) also have a high rDNA ratio (more than 0.2‰), confirming the presence of microbial sequences in some viromes. Even if these two indices revealed similar trends, we had to verify that sequences similar to microbial genomes highlighted in the MHR are not bona fide viral sequences similar to prophages (which are annotated as prokaryotic).

### 4.3. Is cellular-like DNA in fact prophage-like DNA?

Genomic studies have revealed the prevalence of prophages in many and diverse prokaryotes [37]. We therefore hypothesized that some virome reads are similar to bacterial genomes not because of a cellular origin but because of a similarity to a prophage. To confirm this assumption, prophage-like regions in prokaryotic genomes were identified. We detected 55 837 prophage-like regions in the 1312 genomes analysed, which encompassed 11% of the genes in the considered genomes. Virome reads similar to prophage-like regions were then identified and a PHR was calculated, which spanned from 4.5 to 84.9% (37.7% on average; figure 1*b*; electronic supplementary material, table S2) in the analysed viromes. The PHR was also computed for microbiomes (10.1% on average) and, as expected, was very close to the proportion of prophage-like genes in microbial genomes.

To gain a more accurate view of prophage importance within microbial hits from viromes, the MHR and the PHR were plotted simultaneously for both microbiomes and viromes. The resulting plot reasserted that the 67 viromes investigated exhibited different characteristics consistent with the rDNA ratio observations (figure 1*b*; electronic supplementary material, table S2):

— Viromes devoid of rDNA (depicted in green in figure 1) are clearly distinct from microbial metagenomes: microbial-like sequences in these viromes are rare (low MHR, 1.3% on average), and most of them match prophage-like regions (high PHR, 48.7% on average). These results further support the conclusion that these datasets can be considered as viromes *sensu stricto*.
— Viromes with low rDNA ratio (depicted in orange in figure 1) display low MHRs and high PHRs (average of 2.7% and 47.5%, respectively), indicating that most viromes in this category contain only a few microbial sequences.
— Viromes with high rDNA ratio (depicted in red in figure 1) are indistinguishable from microbial metagenomes. Indeed, the average MHR and PHR values for viromes in this category and microbiomes are very similar (MHR: 16.7% versus 15.8%; PHR: 12.4% versus 10.1%, for ‘red’ viromes and microbial metagenomes, respectively), strongly indicating that these viromes contain numerous microbial sequences.

**Figure 2.**
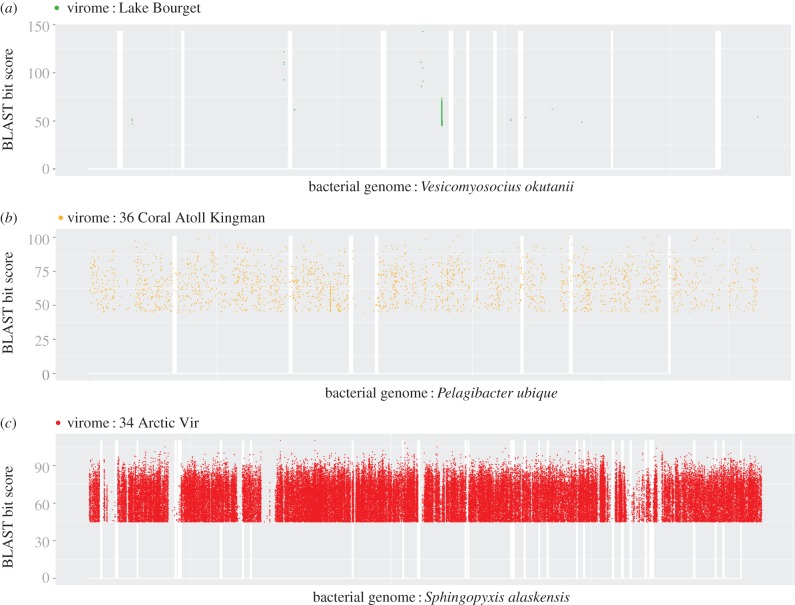
Recruitment plots for three virome–microbial genome associations. Virome reads were affiliated to the KEGG genome with the best tBlastx score. Reads were then plotted at the position of the hit on the corresponding genome (*x*-axis), the sequence conservation being displayed as the identity percentage between read and genome on the *y*-axis. (*a*) 17 444 reads of the Lake Bourget virome are recruited by *Candidatus Vesicomyosocius okutanii*. (*b*) ‘36 Coral Atol’ reads recruited by *Pelagibacter ubique* (1973 reads). (*c*) Recruitment of 91 315 reads from the ‘34 Arctic Vir’ virome by the genome of the Alphaproteobacteria *Sphingopyxis alaskensis*.

Recruitment plots as well as genome coverage ratio generated for selected virome–genome pairs (pairs with a low PHR; see electronic supplementary material) confirmed these observations. For ‘green’ and ‘orange’ viromes, the reads similar to non-prophage genes were often restricted to specific regions, and thus likely to be unpredicted prophage-like region or unknown genes shared by viruses and prokaryotes (figure 2*a*; electronic supplementary material, table S3). Alternatively, low and scattered coverage of bacterial genomes could also result from the rare and random integration of bacterial DNA in generalized transducing phage genomes. Conversely, all recruitment plots for ‘red’ viromes displayed a hit distribution throughout all bacterial genomes with high gene coverage ratios (figure 2*b*; electronic supplementary material, table S3). A virome from Arctic Sea samples [38] represents one of the most striking examples of a virome containing bacterial genomic DNA. Recruitment analysis showed that 91 315 reads from this virome can be matched with *Sphingopyxis alaskensis* (figure 2*c*), covering almost the entire genome.

These results confirm that low rates of affiliation to microbes are mainly owing to prophage-like hits, whereas high rates of affiliation to microbes (correlated to a significant detection of rDNA) are clearly linked to the presence of cellular DNA in samples. However, two different routes of acquisition of this bacterial DNA seem to exist. Indeed, the presence of cellular DNA in eukaryote-associated samples is consistent with potential shortcomings in experimental protocols, as virus-like particles (VLPs) are described as difficult to purify from such matrices [39]. Thus, even the most elaborate protocols are likely to be susceptible to residual contamination with microbial cells or free extracellular nucleic acids. More surprisingly, even though the purification of VLPs from aquatic samples seems possible, the presence of cellular DNA was still detected in several aquatic viromes. Thereby, methodological constraints may not be the sole factor explaining the detection of microbial sequences in viromes.

### 4.4. Source of prokaryotic sequences: gene transfer agents are invited to the party

Several types of VLPs were described as containing genetic material coming from a cellular genome, the most well-known being the GTAs [17,40]. GTAs are host-encoded virus-like elements that package random fragments of the host chromosome [29]. Structurally, GTAs resemble small-tailed phages [41], but do not possess any of the properties (e.g. plaque formation, transmission of viral genes) that are typically associated with phages [41,42]. In our attempt to identify the origin of prokaryotic material in viromes, we verified the viability of the ‘GTA hypothesis’ presented by Kristensen *et al*. [17].

To this purpose, each prokaryotic genome from KEGG database was analysed for the presence of potential GTA gene clusters similar to the four GTA gene clusters reported previously (see electronic supplementary material, table S4). We identified 72 prokaryotic strains (approx. 6% of the known prokaryotic genomes), predominantly affiliated to the **α*-proteobacteria*, containing putative GTA gene clusters (see electronic supplementary material, table S4). We then identified for each ‘red’ virome how many genomes, among the 50 most detected, exhibited GTA gene clusters (see electronic supplementary material, table S3). From this analysis, a dichotomous distribution of viromes emerged:

— Eukaryote-associated samples appear to be free from GTA as only approximately 9% of the bacterial genomes detected in viromes displayed GTA gene clusters.
— Marine samples could contain a significant amount of GTA particles, as more than 50% of the bacterial genomes retrieved in viromes contained at least one GTA cluster. Accordingly, the presence of microbial sequences in seawater viromes could be linked to GTAs rather than to technical limits.

This high ratio of sequences similar to GTA-encoding bacterial genomes in marine viromes is consistent with the high abundance of GTA particles predicted in marine bacterioplankton [43]. However, the definition used to detect GTA gene cluster is likely to include defective prophages, which most probably lead to an over-estimation of GTA in microbial genomes. In any case, GTA now identified in many diverse prokaryotes and particularly in marine *Roseobacter* [31,32] could be of major importance for directed gene transfer between phylogenetically related bacteria in low-density habitats such as seawater.

### 4.5. Towards a new picture of virus-associated functional profiles

**Figure 3.**
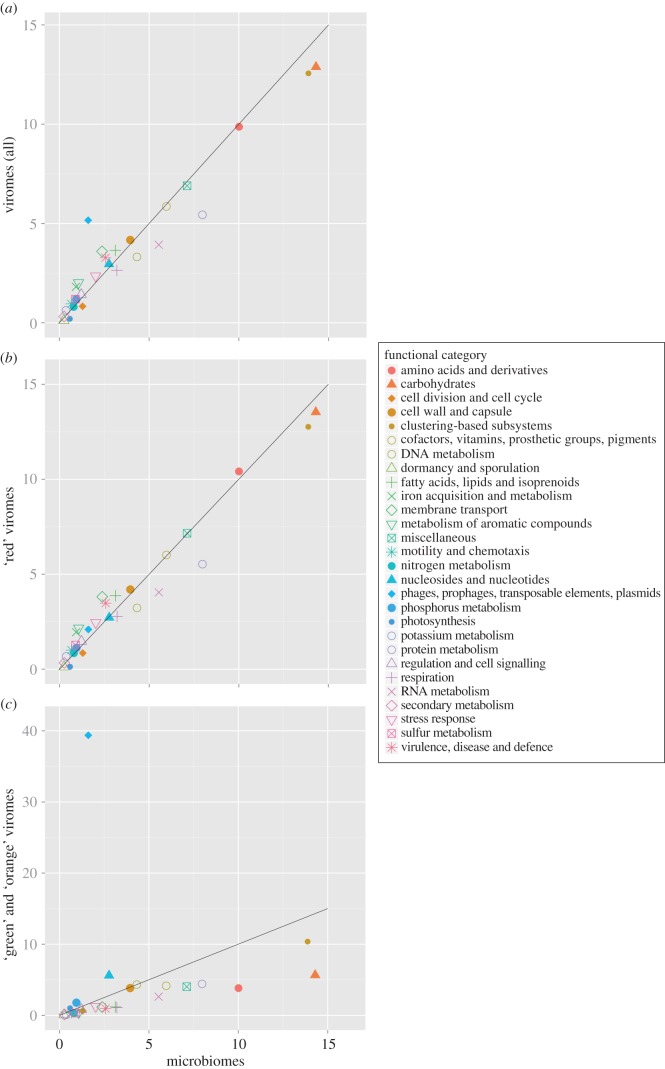
Comparison of the functional profiles of viromes and microbiomes, considering (*a*) all viromes, (*b*) viromes with clearly identified microbial-originating sequences (‘red’ viromes) and (*c*) viromes considered as mostly composed of viral sequences (‘green’ and ‘orange’ viromes). The percentage of reads affiliated to each SEED category (level 1) is indicated for microbiomes (*x*-axis) and viromes (*y*-axis).

Viromes are usually considered as entirely composed of viral sequences, and therefore used to determine the functions encoded in genomes of environmental viral communities. When comparing functional profiles, the enrichment in VLPs in viromes does not result in significant differences between viromes and microbial metagenomes (Pearson correlation coefficient of 0.93; figure 3*a*). This is consistent with a previous observation [16], and was suggested to result from both the high number of genes exchanged between viral and microbial genomes and the registered functional categories in databases which describe cellular rather than viral functions [17]. Yet another explanation could be that the presence of prokaryotic DNA in viromes introduces a bias into functional profile analyses [17]. Following identification of viromes containing cellular sequences, we postulated that a new picture of the functional profiles of viral communities might emerge from these data. To test this hypothesis, we computed functional profiles for two sets of viromes using their rDNA ratios: (i) viromes with clearly identified microbial-originating sequences (‘red’ viromes; figure 3*b*) and (ii) viromes considered as mostly composed of viral sequences (‘green and orange’ viromes; figure 3*c*). The functional profiles obtained were very different (figure 3*b,c*). The functional profile of the first category of viromes was strongly correlated to the profile of microbiomes (Pearson correlation coefficient of 0.98), and the typical viral category ‘phages, prophages, transposable elements, plasmids’ ranked only at the 17th position in these viromes (2.09% of the functions; figure 3*b*). Conversely, a low correlation was found between functional profiles of the second category of viromes and microbiomes (Pearson correlation coefficient of 0.18), and these viromes displayed a strong enrichment in phage-like genes (39.8% for ‘phages, prophages, transposable elements, plasmids’). Furthermore, prevalence of other categories in viromes and microbiomes was also no longer equivalent: ‘green’ and ‘orange’ viromes were depleted of typical cellular categories rarely observed in sequenced phages (e.g. ‘cofactors, vitamins, prosthetic groups, pigments’), but cellular categories commonly identified in known phages were retrieved (e.g. ‘nucleosides and nucleotides’, ‘DNA metabolism’; figure 3*c*).

From this analysis, we demonstrated that the presence of bacterial DNA in several viromes biased the previous functional analyses of viromes, leading to an artefactual correlation between functional profiles of viromes and microbiomes. Even if all functional categories are retrieved in ‘viral-only’ viromes, indicating that all types of bacterial genes could be carried by the viral community, their proportions in viromes are highly different from those in microbiomes. Moreover, cellular sequences in viromes can have significant effects on the conclusions drawn from the functional analyses of these datasets. For example, the category ‘motility and chemotaxis’ enriched in viromes compared with microbiomes (1.00% and 0.66%) has been previously proposed as ‘an unexpected example of specialized metabolisms being carried within the viromes’ [16], but, according to our analysis, we postulate that this result was artefactual and linked to the presence of cellular DNA in viromes (enrichment of only 0.37% for ‘green’ and ‘orange’ viromes; figure 3*c*).

### 4.6. Viral pan-genome encompasses an unexpected diversity of metabolism genes

Owing to its numerical vastness and genetic diversity, the virosphere is expected to embrace a tremendous functional potential. However, the extent of this potential remains unclear. Furthermore, the finding that a number of published viromes is also composed of cellular sequences suggests that conclusions originally drawn from the analyses of the complete set of viromes might be inaccurate for depicting the functional potential of viruses *per se*. Obviously, the validity of the results is directly proportional to the ‘purity’ of the analysed dataset, and even if this presumed slight presence of non-viral DNA (‘orange’ viromes) generates only a background noise in a large spectrum analysis such as functional profiling, it can bias the results when considering cellular functions one by one. Thus, in order to increase the likelihood of functional assignments being associated with viruses rather than cellular organisms, we hereafter only considered ‘green’ viromes, in which no rDNA sequences were found.

A total number of 1233 different KEGG orthology (KO) groups were detected in this dataset from the total of 14 645 KO groups present in KEGG database. Comparison of these 1233 KO groups against the viral RefSeq sequences showed that 30% of them are represented in complete viral genomes. The most retrieved KO groups are often those already associated with viruses: 75% of the highly retrieved KO (associated to more than 20 sequences) are also represented in complete viral genomes, including proteins involved in all steps of viral infection cycle (i.e. virion morphogenesis, viral genome transcription, replication, recombination and repair, as well as cell lysis). These functional categories are well represented in the currently available viral genomes and will not be further discussed. Perhaps more unexpected was the identification of diverse protein functions responsible for modulation of cellular metabolism and virus–host interactions. Below, we briefly outline the most prominent KEGG functional categories retrieved and highlight potential roles of these proteins in the framework of viral infection cycles.

### 4.7. Energy metabolism genes

**Figure 4.**
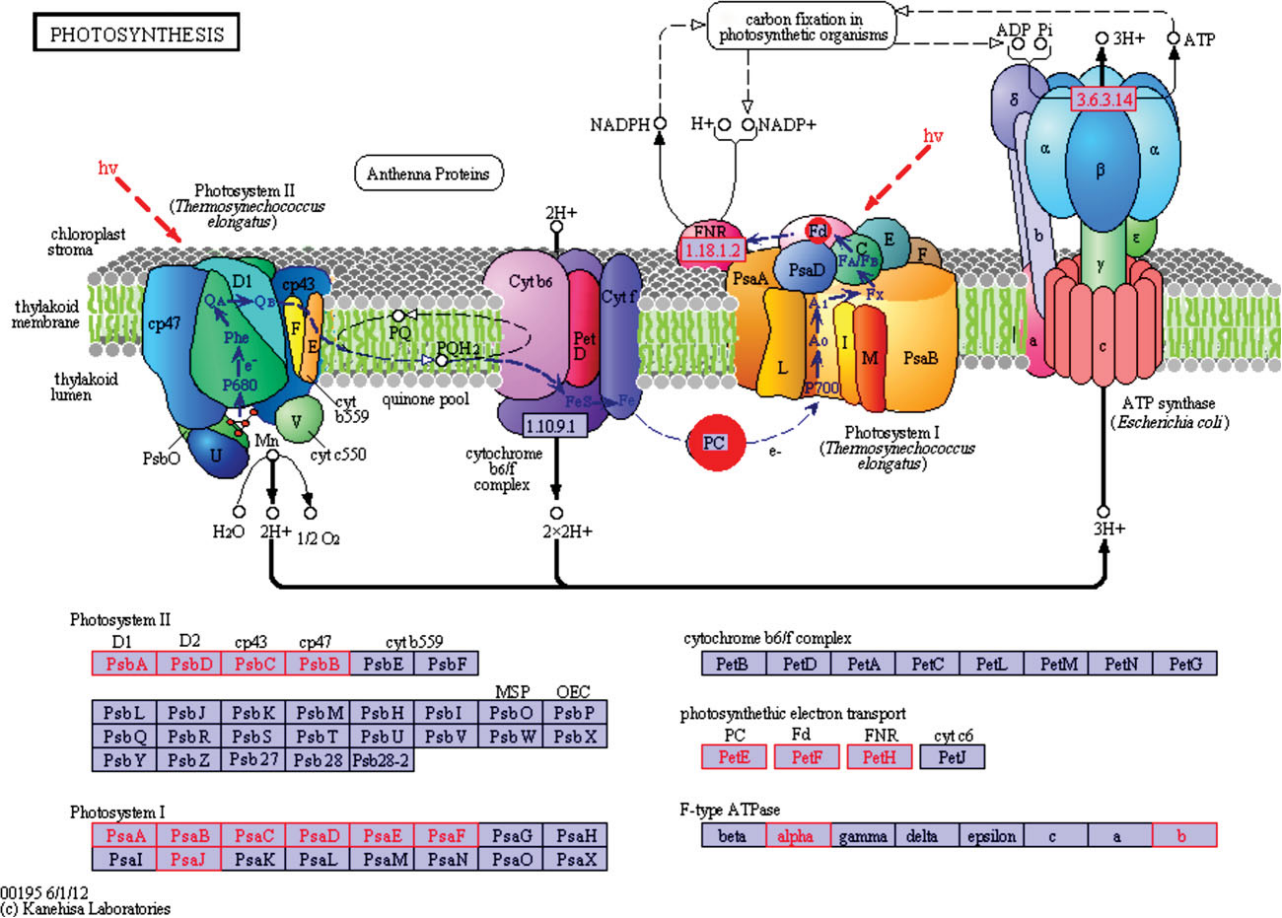
Mapping of virome-retrieved functions on the different types of photosystem. On this general representation of the photosystems, KO retrieved in uncontaminated viromes are highlighted in red on the list of KO at the bottom, and when possible on the chart at the top.

**Figure 5.**
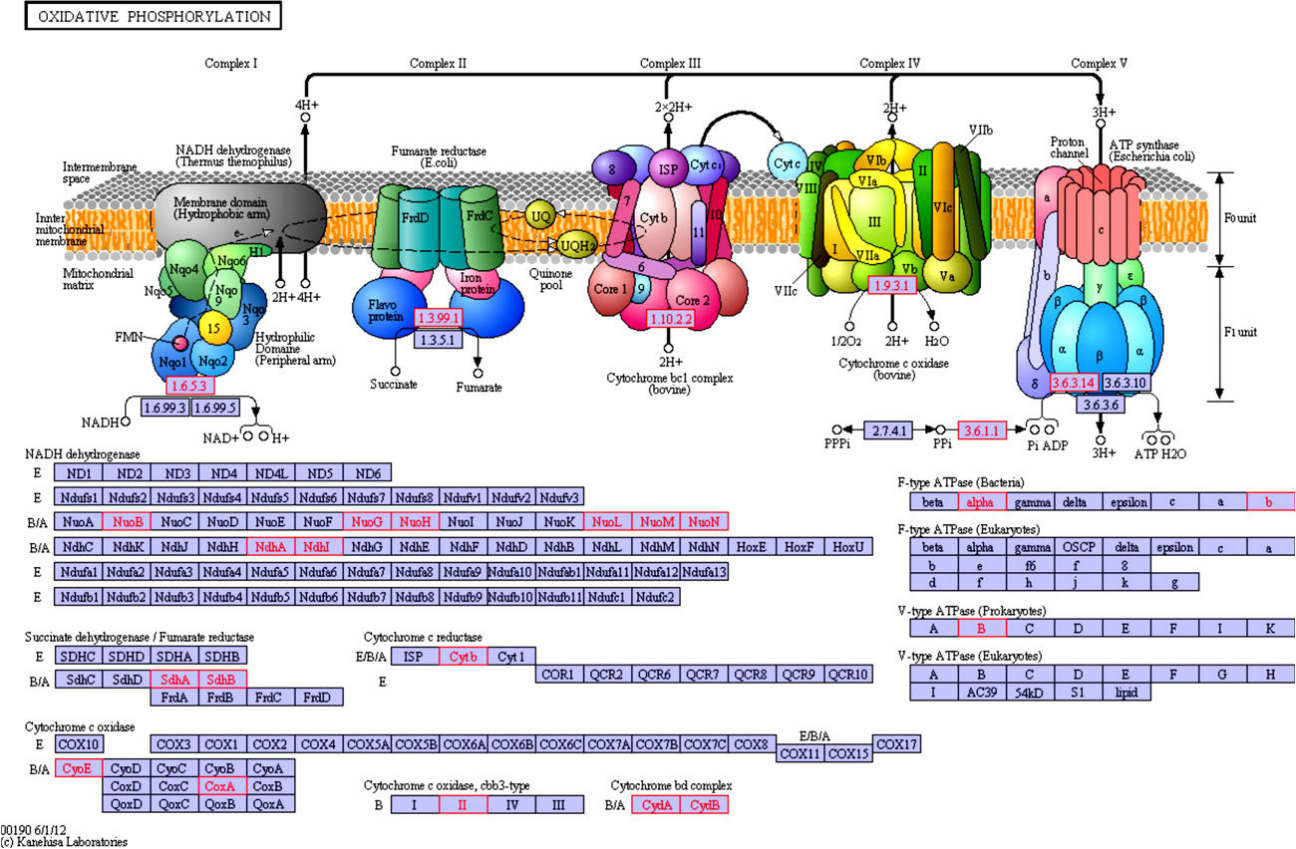
Mapping of virome-retrieved functions on oxydative phosphorylation pathway. On this general representation of the oxydative phosphorylation pathway, KO retrieved in uncontaminated viromes are highlighted in red on the list of KO at the bottom, and when possible on the chart at the top.

One of the landmark discoveries of the past decade was the identification of functional photosystem (PS) II genes in cyanophage genomes [9]. More recently, metagenomics analysis revealed that marine cyanophages might also encode the entire suite of proteins composing PSI (seven proteins) [44]. These findings have clearly demonstrated that viruses may play an active role in energy transformation. In accordance with previous results, our list of KO groups included components of both PSII (including proteins D1 and D2) and PSI (including PsaA and PsaB; table 1 and figure 4). These photosynthesis genes did not present the same pattern of distribution: PSI genes were found exclusively in marine viromes, while those of PSII were also present in freshwater and hypersaline environments (see electronic supplementary material, table S5). Surprisingly, our analysis suggests that besides photosynthesis genes viruses may encode a set of proteins involved in oxidative phosphorylation. We identified several components of the prokaryotic electron transport chain *Complexes I*, *II, III* and *IV* (table 1 and figure 5). Intriguingly, it appears that viruses might also harbour genes for at least some subunits (α, β) of the *F_0_F_1_ ATP synthase* (also referred to as Complex V) as well as genes for inorganic *pyrophosphatase* (Ppa), which is responsible for supplying inorganic phosphate for ATP synthesis by ATP synthase. Notably, the latter set of enzymes might also operate in conjunction with the photosystem genes. Indeed, genes for the *a*, *b* and *c* subunits of the *F_0_F_1_ ATP synthase* have recently been reported in the environmental Global Ocean Sampling (GOS) cyanophage clone JCVI_SCAF_1096628171668 [45]. Similarly, metagenomic studies have previously suggested that cyanophages might harbour the ndhI, ndhD and ndhP genes of the Complex I [44,45]. Finally, we found both subunits (CydA and CydB) of the two-component *cytochrome bd quinol oxidase*, which is associated with microaerobic dioxygen respiration [46].

**Table 1.** Subset of KO retrieved more than five times in non-contaminated viromes, never described in complete viral genomes, and implicated in selected pathways. The complete list of KO retrieved in the nine viral-only viromes is available as electronic supplementary material, table S5.

KO category/ID	KO name	KO definition	no. reads	no. viromes
ko00195 photosynthesis				
K02689	psaA	photosystem I P700 chlorophyll a apoprotein A1	52	1
K02690	psaB	photosystem I P700 chlorophyll a apoprotein A2	50	1
K02691	psaC	photosystem I subunit VII	10	1
K02692	psaD	photosystem I subunit II	10	1
K02705	psbC	photosystem II CP43 chlorophyll apoprotein	6	2
ko00190 oxidative phosphorylation				
K00240	sdhB	succinate dehydrogenase iron-sulfur protein [EC:1.3.99.1]	18	1
K00412	CYTB, petB	ubiquinol-cytochrome *c* reductase cytochrome *b* subunit [EC:1.10.2.2]	7	2
K00425	cydA	cytochrome *bd*-I oxidase subunit I [EC:1.10.3.-]	15	1
K02274	coxA	cytochrome *c* oxidase subunit I [EC:1.9.3.1]	7	2
K05580	ndhI	NADH dehydrogenase I subunit I [EC:1.6.5.3]	66	1
ko00010 glycolysis/gluconeogenesis				
K00162	PDHB, pdhB	pyruvate dehydrogenase E1 component subunit beta [EC:1.2.4.1]	37	3
K01623	ALDO, fbaB	fructose-bisphosphate aldolase, class I [EC:4.1.2.13]	12	1
ko00020 citrate cycle (TCA cycle)				
K00162	PDHB, pdhB	pyruvate dehydrogenase E1 component subunit beta [EC:1.2.4.1]	37	3
K00240	sdhB	succinate dehydrogenase iron-sulfur protein [EC:1.3.99.1]	18	1
ko00030 pentose phosphate pathway				
K00615	E2.2.1.1, tktA, tktB	transketolase [EC:2.2.1.1]	59	3
K01623	ALDO, fbaB	fructose-bisphosphate aldolase, class I [EC:4.1.2.13]	12	1
K01808	E5.3.1.6B, rpiB	ribose 5-phosphate isomerase B [EC:5.3.1.6]	10	3
ko00520 amino sugar and nucleotide sugar metabolism				
K00523	ascD, ddhD, rfbI	CDP-4-dehydro-6-deoxyglucose reductase [EC:1.17.1.1]	7	2
K00790	murA	UDP-*N*-acetylglucosamine 1-carboxyvinyltransferase [EC:2.5.1.7]	12	2
K00978	rfbF	glucose-1-phosphate cytidylyltransferase [EC:2.7.7.33]	6	1
K00983	E2.7.7.43, neuA, CMAS	*N*-acylneuraminate cytidylyltransferase [EC:2.7.7.43]	11	5
K01654	E2.5.1.56, neuB	*N*-acetylneuraminate synthase [EC:2.5.1.56]	126	8
K01709	rfbG	CDP-glucose 4,6-dehydratase [EC:4.2.1.45]	6	3
K01809	E5.3.1.8, manA	mannose-6-phosphate isomerase [EC:5.3.1.8]	17	3
K03431	glmM	phosphoglucosamine mutase [EC:5.4.2.10]	5	2
K12454	rfbE	CDP-paratose 2-epimerase [EC:5.1.3.10]	19	4
ko00540 lipopolysaccharide biosynthesis				
K02535	lpxC	UDP-3-O-[3-hydroxymyristoyl] *N*-acetylglucosamine deacetylase [EC:3.5.1.-]	5	2
K02536	lpxD	UDP-3-O-[3-hydroxymyristoyl] glucosamine *N*-acyltransferase [EC:2.3.1.-]	12	2
K02843	waaF, rfaF	heptosyltransferase II [EC:2.4.-.-]	18	2
ko00550 peptidoglycan biosynthesis				
K00790	murA	UDP-*N*-acetylglucosamine 1-carboxyvinyltransferase [EC:2.5.1.7]	12	2
ko00970 aminoacyl-tRNA biosynthesis				
K01872	AARS, alaS	alanyl-tRNA synthetase [EC:6.1.1.7]	7	4
K04567	KARS, lysS	lysyl-tRNA synthetase, class II [EC:6.1.1.6]	18	2
ko03010 ribosome				
K02945	RP-S1, rpsA	small subunit ribosomal protein S1	17	3
K02970	RP-S21, rpsU	small subunit ribosomal protein S21	18	4

### 4.8. Carbon metabolism genes

Unexpectedly, the dataset contained a substantial number of enzymes involved in such fundamental cellular metabolism pathways as glycolysis, tricarboxylic acid (TCA) cycle and pentose phosphate pathway (PPP) (table 1; electronic supplementary material, table S5). With few exceptions, genes of this category are not typically found in viral genomes.

#### 4.8.1. Glycolysis

Glycolysis is a universal metabolic pathway of converting glucose into pyruvate and generating small amounts of the high-energy compounds adenosine triphosphate (ATP) and nicotinamide adenine dinucleotide (NADH). The glycolytic breakdown of glucose in anaerobic or severely hypoxic conditions is the sole source of ATP for many microorganisms. We identified 11 KO groups that were related to glycolysis pathway and detected more than once in viromes (table 1; electronic supplementary material, table S5). A growing body of evidence suggests that viruses might modulate the host metabolism according to their needs. For example, it has been suggested that cyanophage-encoded proteins may modify the photosynthetic electron transfer chain such that the cyclic electron flow around PSI would be favoured over the linear one, leading to preferential production of ATP [45]. In this light, it is tempting to speculate that the viral versions of glycolysis enzymes might be differentially susceptible to allosteric regulation compared with their cellular counterparts so as to maximize the energy production for optimal virus replication.

#### 4.8.2. Tricarboxylic acid cycle and pyruvate metabolism

In aerobic conditions, glycolysis, fat and protein catabolic pathways converge on the TCA cycle. As a result, carbohydrates, fatty acids and amino acids are oxidized to CO_2_ with most of the energy of oxidation temporarily held in the electron carriers FADH_2_ and NADH, which eventually enter the respiratory chain where the energy of electron flow is converted to ATP. Thus, the TCA cycle represents the central catabolic pathway in aerobic organisms. We identified 10 non-singleton virome-associated KO groups involved in the TCA cycle (KO groups detected more than once in viromes), including *pyruvate dehydrogenase* (E1 subunits α and β), which is responsible for converting pyruvate generated during glycolysis into acetyl-CoA. In addition, 11 non-singleton KO groups were found to be affiliated with the pyruvate metabolism pathway (ko00620) (table 1; electronic supplementary material, table S5).

#### 4.8.3. Pentose phosphate pathway

Ten non-singleton KO groups in our dataset mapped to the PPP, which represents an alternative route of glucose metabolism. PPP is a two-phase pathway leading to production of reducing equivalent NADPH (during oxidative phase) and pentose phosphates for synthesis of nucleotides and amino acids (during non-oxidative phase). It has previously been demonstrated that some cyanophages encode functional homologues of cyanobacterial *transaldolase* (TalC) [47,48], *6-phosphogluconate dehydrogenase* (Gnd) and *glucose-6-phosphate 1-dehydrogenase* (G6PD) [49], key enzymes of the PPP. TalC, Gnd and G6PD were all retrieved in our analysis among high-confidence virome-associated KO groups. In addition to the three enzymes mentioned above*,* our data suggest that viruses carry genes for other PPP enzymes, including *transketolase* (Tkt), *phosphoribosyl pyrophosphate synthase* (PRPS), *ribose-5-phosphate isomerase* (rpiB) and *fructose-biphosphate aldolase of class I* and *II* (fbaB and fbaA; table 1; electronic supplementary material, table S5)*.* Notably, G6PD catalyses the first, essentially irreversible reaction in the oxidative phase of the PPP and is the rate-limiting enzyme of the pathway. Expression of viral G6PD might thus stimulate PPP, indicating that this pathway is beneficial for virus replication. Indeed, it has been shown that cyanophages specifically direct carbon flflux away from the Calvin cycle towards the PPP, this way ensuring that the ATP and NADPH produced by photosynthesis are not consumed in the Calvin cycle but are rather used to fuel phage dNTP biosynthesis [47]. This is consistent with the identification of the virome-associated genes for PRPS, one of the key enzymes in the *de novo* and *salvage* biosynthesis of nucleotides.

### 4.9. Translation genes

Sequencing of the Mimivirus genome revealed that viruses might occasionally encode proteins involved in translation, such as aminoacyl tRNA synthetases (aaRS), and translation initiation and elongation factors [50]. This finding has subsequently been confirmed by additional genome sequences of large eukaryotic [51,52] and, more recently, bacterial [53] viruses. To date, members of the *Mimiviridae* were found to encode seven different aaRS—ArgRS, TyrRS, CysRS, MetRS, IleRS, TrpRS and AsnRS [50–52]—while *Bacillus megaterium* phage G carries a gene for SerRS [53]. In the uncontaminated viromes, we identified aaRS genes specific for 18 of the 20 proteinogenic amino acids, as well as several genes for enzymes involved in the modification of aminoacyl-tRNAs, including methionyl-tRNA formyltransferase (required for formation of formylMet-tRNA, an initiator tRNA in bacteria, mitochondria and chloroplasts) and aminoacyl-tRNA amidotransferase (table 1; electronic supplementary material, table S5). In addition, we found genes for translation initiation (IF-1, 2 and 3), elongation (EF-G) and peptide chain release (RF-1 and RF-3) factors.

As expected, no rRNA genes were retrieved. However, several rRNA modification enzymes, such as rRNA methyltransferases and rRNA pseudouridine synthase, were identified. Finally, a set of six non-singleton ribosomal proteins were also present in the filtered dataset (table 1; electronic supplementary material, table S5). To our knowledge, there are no precedents of ribosomal proteins being encoded by viruses. Thus, it is not clear whether the two genes signify the presence of cellular sequences or genuine gene acquisitions by viruses. However, the point can be made that there is no obvious reason why these ribosomal protein genes, which are detected up to 18 times within four different viromes, should be recovered in the viral fraction to the exclusion of all other ribosomal genes, including those for rRNA, which are often present in multiple copies per cellular genome and are statistically more likely to be identified among cellular-originating sequence [54]. Ribosomal protein genes are known to be transferred horizontally [55–58], although the particular routes of such transfer remain unclear. One possibility, which might be strengthened by observations presented above, is that viruses serve as vehicles for horizontal transfer of ribosomal protein genes, as is the case with many other cellular genes [59]. What could be a role of ribosomal protein in the course of a viral cycle? Modification of the ribosomes by viral versions of the ribosomal proteins might allow viruses to overcome a translational shutoff in the host, which may be triggered by viral infection. Indeed, bacterial viruses are known to induce the toxin components of certain toxin–antitoxin systems [60], some of which are known to poison or stall the ribosomes [61]. Alternatively, many ribosomal proteins perform extraribosomal functions, a phenomenon known as moonlighting [62,63]. Notably, protein S1, one of the most detected in our dataset, is one of such proteins; in addition to being a structural component of the ribosomes, S1 regulates expression of several ribosomal operons, including its own [62]. Finally, Qβ and other leviviruses hijack S1 to serve as a subunit of their RNA replicases [64]. It is thus possible that viruses recruit ribosomal protein genes for functions that have little to do with ribosome structure.

Peculiarly, ribosomes represent one of the final frontiers distinguishing viruses and cellular organisms [65], at least from the genomic perspective. Additional efforts focused on exploration of genetic diversity in the virosphere, and especially these intriguing ribosomal proteins, are undoubtedly needed to resolve this puzzle.

## 5. Conclusion

The putative presence of non-viral sequences in viromes undoubtedly raises questions about these datasets, but must not be seen as challenging all previous results and conclusions. Indeed, the presence of cellular DNA in viromes certainly has little effect on the analysis and interpretation of sequences that can be unequivocally assigned to viruses (i.e. when reasonably close homologues are present in the genomes of cultivated viruses), as was the case in most virome studies published. However, questions related to functional capacity of uncultured viral communities, and specifically the diversity of microbial-like genes in viral genomes, require all sequences in the viromes to be of viral origin in order to be rigorously addressed. If the latter point is neglected, the validity and value of conclusions drawn from the virome analyses become questionable, as illustrated by the results presented in this study.

Our study also pinpoints the different sources of cellular sequences in viromes obtained from different environments, stressing the role of GTAs in the case of seawater samples. Unfortunately, as GTAs display a viral capsid structure, it is probable that no preparation step will be able to separate them from actual viral capsids, and hence this type of ‘contamination’ is probably irremediable. Moreover, beyond GTAs, other bacteria-produced elements such as DNA-containing membrane vesicles can also be confused with viral particles [40], thus being potential entry points for cellular DNA in viromes. In such cases, downstream bioinformatics analysis will be needed to check their presence in viromes.

Ultimately, one of the most significant findings resulting from this analysis was the abundance and global distribution of virome-associated operational (metabolic) genes. Indeed, it appears that in all analysed biomes, viruses intensively tinker with the metabolism of their hosts. A great deal of functional and genomic data on photosynthetic genes in cyanophages made this viral group stand out as an exception, or a peculiarity within the virosphere in the eyes of many (micro)biologists. Here, we provided evidence suggesting that beside photosynthesis, viruses might tap into such central metabolic pathways as oxidative phosphorylation, glycolysis, TCA and PPP. It is noteworthy that some of these metabolic enzymes have been previously identified in viral genomes. In order to validate these observations (e.g. the presence of ribosomal protein S1 in viral genomes), methods such as gene-targeted metagenomics [66] could help one to get a genomic context for gene(s) of interest. Although the available scattered data did not allow one to draw generalizing conclusions on the role of viruses in the cellular metabolism beyond particular virus–host systems, our analysis of viromes issued from diverse environments illuminates a somewhat unexpected picture of global ‘viral’ metabolism, suggesting that viruses might actively dictate the metabolism of infected cells on a global scale.

## Supplementary Material

Supplementary material
