# Supplementary material for: Assessment of viral community functional potential from viral metagenomes may be hampered by contamination with cellular sequences
Source: Open Biol. 2013 Dec;3(12):130160. doi: 10.1098/rsob.130160 (PMC3877843; doi:10.1098/rsob.130160)
Supplement: Supplementary material [file rsob130160supp1.pdf]

Table S1 : **List of viromes and microbiomes used in this study.** The web-servers hosting the datasets are: NCBI (www.ncbi.nlm.nih.gov), MG-Rast (http://metagenomics.anl.gov), and Metavir (http://metavir-meb.univ-bpclermont.fr). Each virome and microbiome is identified by an Id. throughout the paper. When available, the methodology used to purify viral particle is indicated (CsCl : Cesium Chloride, PEG : Polyethylene Glycol, LASL : linker amplified shotgun library and MDA : phi29-mediated multiple displacement amplification).

Virome id	Virome name	Available on	Methodology used in sample preparation	Sample origin	Sample type	Number of sequences	Mean size of sequences
12	Medium viruses (MP1128)	MG-Rast – 4440427.3	PEG – CsCL- MDA	Solar Salterns – California	Hypersaline	39,439	100.43
13	Medium viruses (MP1116)	MG-Rast – 4440428.3	PEG – CsCL- MDA	Solar Salterns – California	Hypersaline	58,319	98.11
14	High saltern viral (HP1116)	MG-Rast – 4440421.3	PEG – CsCL- MDA	Solar Salterns – California	Hypersaline	151,180	99.76
15	Low saltern (Pond 11) viruses	MG-Rast – 4440436.3	PEG – CsCL- MDA	Solar Salterns – California	Hypersaline	268,049	104.47
16	Low saltern (Pond 11) viruses (LP1110)	MG-Rast – 4440432.3	PEG – CsCL- MDA	Solar Salterns – California	Hypersaline	109,836	104.35
17	Medium saltern viruses (Pond MP1110)	MG-Rast – 4440431.3	PEG – CsCL- MDA	Solar Salterns – California	Hypersaline	39,348	101.40
18	Medium saltern viruses (MP1122)	MG-Rast – 4440417.3	PEG – CsCL- MDA	Solar Salterns – California	Hypersaline	55,142	100.79
19	High saltern viruses (HP1207)	MG-Rast – 4440145.4	PEG – CsCL- MDA	Solar Salterns – California	Hypersaline	46,628	102.15
20	High saltern viral (HP1128)	MG-Rast – 4440144.4	PEG – CsCL- MDA	Solar Salterns – California	Hypersaline	4,536	100.15
21	Low saltern viruses (LP1128)	MG-Rast – 4440420.3	PEG – CsCL- MDA	Solar Salterns – California	Hypersaline	62,363	103.87
22	Salton Sea Phage 1	MG-Rast – 4440327.3		Salton Sea – California	Hypersaline	55,467	103.60
23	Salton Sea Phage 3	MG-Rast – 4440328.3		Salton Sea – California	Hypersaline	29,814	99.20
32	Marine Phages GOM	MG-Rast – 4440304.3	CsCL – MDA	Gulf of Mexico	Seawater	262,501	101.59
33	Marine Phages BBC	MG-Rast – 4440305.3	CsCL – MDA	British Columbia	Seawater	414,964	102.14
34	Marine Phages Arctic	MG-Rast – 4440306.3	CsCL – MDA	Arctic sea	Seawater	686,209	99.15
35	Marine Phage SAR	MG-Rast – 4440322.3	CsCL – MDA	Sargasso sea	Seawater	397,939	104.31
36	Line Islands Kingman Reef B2 phage	MG-Rast – 4440036.3	CsCL – MDA	Kingmann - Line Islands	Seawater	93,744	108.34
37	Line Islands Christmas Reef B3 phage	MG-Rast – 4440038.3	CsCL – MDA	Christmas - Line Islands	Seawater	279,882	110.56
38	Line Islands Palmyra F8 Phage	MG-Rast – 4440040.3	CsCL – MDA	Palmyra - Line Islands	Seawater	318,178	104.78
39	Line Islands Tabuaeran B1 Phage	MG-Rast – 4440280.3	CsCL – MDA	Tabuaeran - Line Islands	Seawater	378,475	104.13
40	Tampa Bay phage from induction experiment	MG-Rast – 4440102.3	PEG – CsCL- MDA	Tampa Bay – Florida	Seawater	279,129	103.95
41	Skan Bay Phage 1	MG-Rast – 4440330.3		Skan Bay – Alaska	Seawater	30,831	104.56
46	Tpond phage 3	MG-Rast – 4440424.3	PEG – CsCL- MDA	Tilapia Pond 3 – California	Freshwater	56,549	101.06
47	Healthy Tilapia pond phages	MG-Rast – 4440412.3	PEG – CsCL- MDA	Healthy fish Pond – California	Freshwater	60,135	101.07
48	Healthy Prebead tank phages	MG-Rast – 4440414.3	PEG – CsCL- MDA	Prebead Pond – California	Freshwater	67,785	103.21
49	Tilapia pond	MG-Rast – 4440439.3	PEG – CsCL- MDA	Tilapia Pond – California	Freshwater	264,844	102.25
57	Porites compressa time zero viruses	MG-Rast – 4440376.3	CsCL – MDA	Porites Compressa	Eukaryote	39,113	101.32
58	Porites compressa control treated viruses	MG-Rast – 4440374.3	CsCL – MDA	Porites Compressa	Eukaryote	39,191	103.70
59	Porites compressa DOC treated viruses	MG-Rast – 4440370.3	CsCL – MDA	Porites Compressa	Eukaryote	35,409	102.18
60	Porites compressa pH treated viruses	MG-Rast – 4440371.3	CsCL – MDA	Porites Compressa	Eukaryote	49,949	104.73
61	Porites compressa nutrient treated viruses	MG-Rast – 4440377.3	CsCL – MDA	Porites Compressa	Eukaryote	34,139	107.18
62	Porites compressa temperature treated viruses	MG-Rast – 4440375.3	CsCL – MDA	Porites Compressa	Eukaryote	38,482	113.38
66	Pozas Azules Stromatolites phages	MG-Rast – 4440320.3	CsCL – MDA	Paztac Azules	Microbialites	301,264	104.64
67	Rios Mesquites Stromatolites phages	MG-Rast – 4440321.3	CsCL – MDA	Rio Mesquites	Microbialites	324,500	104.22
68	Highborne Cay Stromatolite phage	MG-Rast – 4440323.3	CsCL – MDA	Bahamas	Microbialites	148,334	100.52
73	Healthy slime viruses	MG-Rast – 4440065.3		Healthy fish gut	Eukaryote	61,022	98.45
74	Morbid slime viruses	MG-Rast – 4440064.3		Morbid fish gut	Eukaryote	59,599	98.32
83	CF Lung Sputum Viruses	MG-Rast – 4440441.3	CsCL – MDA	Human Lung (USA)	Eukaryote	92,223	80.71
84	Health Lung Sputum Viruses	MG-Rast – 4440442.4	CsCL – MDA	Human Lung (USA)	Eukaryote	39,489	84.80
85	Mosquito DNA 1	MG-Rast – 4440052.3	CsCL – MDA	Mosquito (USA)	Eukaryote	336,760	102.61
86	Mosquito DNA 2	MG-Rast – 4440053.3	CsCL – MDA	Mosquito (USA)	Eukaryote	638,689	100.32
87	Mosquito DNA 3	MG-Rast – 4440054.3	CsCL – MDA	Mosquito (USA)	Eukaryote	601,040	104.16
AlSp	Lake Limnopolar Spring	Metavir – Project Lake Limnopolar	Sucrose cushion – MDA	Lake Limnopolar	Freshwater	41,322	239.65
AlSu	Lake Limnopolar Summer	Metavir – Project Lake Limnopolar	Sucrose cushion – MDA	Lake Limnopolar	Freshwater	38,475	221.27
MET6	Lake Pavin	Metavir – Project French Lakes	PEG – MDA	Lake Pavin – France	Freshwater	649,290	412.31
MET7	Lake Bourget	Metavir – Project French Lakes	PEG – MDA	Lake Bourget – France	Freshwater	593,084	433.41
SRR043421	Human Gut L1 8	Metavir – Project Human Gut Diet	CsCL – MDA	Human Gut (USA) – X1	Eukaryote	30,873	372.90
SRR043422	Human Gut L2 1	Metavir – Project Human Gut Diet	CsCL – MDA	Human Gut (USA) – L1	Eukaryote	132,569	379.12
SRR043423	Human Gut L2 7	Metavir – Project Human Gut Diet	CsCL – MDA	Human Gut (USA) – L2	Eukaryote	61,104	370.82
SRR043424	Human Gut L2 8	Metavir – Project Human Gut Diet	CsCL – MDA	Human Gut (USA) – L2	Eukaryote	148,781	370.31
SRR043425	Human Gut H1 7	Metavir – Project Human Gut Diet	CsCL – MDA	Human Gut (USA) – L2	Eukaryote	16,955	375.02
SRR043426	Human Gut H1 8	Metavir – Project Human Gut Diet	CsCL – MDA	Human Gut (USA) – H1	Eukaryote	13,048	378.90
SRR043427	Human Gut H2 8	Metavir – Project Human Gut Diet	CsCL – MDA	Human Gut (USA) – H1	Eukaryote	16,747	365.41
SRR043428	Human Gut L1 1	Metavir – Project Human Gut Diet	CsCL – MDA	Human Gut (USA) – H2	Eukaryote	16,137	366.71
SRR043430	Human Gut H1 2	Metavir – Project Human Gut Diet	CsCL – MDA	Human Gut (USA) – L1	Eukaryote	107,259	405.06
SRR043431	Human Gut H1 1	Metavir – Project Human Gut Diet	CsCL – MDA	Human Gut (USA) – H1	Eukaryote	107,993	409.48
SRR043432	Human Gut H2 1	Metavir – Project Human Gut Diet	CsCL – MDA	Human Gut (USA) – H1	Eukaryote	25,648	377.67
SRR043433	Human Gut L3 1	Metavir – Project Human Gut Diet	CsCL – MDA	Human Gut (USA) – H2	Eukaryote	23,614	372.91
SRR043434	Human Gut L3 2	Metavir – Project Human Gut Diet	CsCL – MDA	Human Gut (USA) – L3	Eukaryote	33,489	369.66
SRR043435	Human Gut L3 7	Metavir – Project Human Gut Diet	CsCL – MDA	Human Gut (USA) – L3	Eukaryote	76,090	384.09
SRR043436	Human Gut L3 8	Metavir – Project Human Gut Diet	CsCL – MDA	Human Gut (USA) – L3	Eukaryote	15,166	382.16
SRR043437	Human Gut F-A	Metavir – Project Human Gut Diet	CsCL – MDA	Human Gut (USA) – L3	Eukaryote	59,155	382.72
SRR089800	Human Gut F-B	Metavir – Project Human Feces	CsCL – MDA	Human Gut (South Korea)	Eukaryote	113,054	431.05
SRR089802	Human Gut F-C	Metavir – Project Human Feces	CsCL – MDA	Human Gut (South Korea)	Eukaryote	109,569	435.21
SRR089803	Human Gut F-D	Metavir – Project Human Feces	CsCL – MDA	Human Gut (South Korea)	Eukaryote	68,391	437.07
SRR089804	Human Gut F-E	Metavir – Project Human Feces	CsCL – MDA	Human Gut (South Korea)	Eukaryote	115,121	433.08
SRR089805	Human Gut Unknown	Metavir – Project Human Feces	CsCL – MDA	Human Gut (South Korea)	Eukaryote	98,511	417.05

Table S2 : **rDNA ratios, Microbial hit ratio (MHR) and prophage hit ratio (PHR) for each metagenome**, with the different data used to calculate these ratios (total number of sequences, number of 16S and 23S rDNA detected, number of sequences with a microbial hits, number of microbial hits within a prophage-like region). rDNA ratios are colored as in Figure 1, *i.e.* green for 0, orange for 0 to 2 ‱, and red for more than 2‱. MHR are highlighted in blue when lower than 5%. PHR are highlighted in red when lower than 10% and in green when greater than 25%.

Virome Id	Name	Biome	Total number of sequences	Number of 16S rDNA detected	Number of 23S rDNA detected	rDNA ‰	Total number of hits with prokaryote genomes	Number of hits in prophage-like region	Number of hits in non-prophage region	Microbial Hit Ratio	Prophage Hit Ratio
12	Medium viruses (MP1128)	Hypersaline	39 439	1	2	0.76	360	198	162	0.91%	55.00%
13	Medium viruses (MP1116)	Hypersaline	58 319	11	32	7.37	1 621	345	1 276	2.78%	21.28%
14	High saltern viral (HP1116)	Hypersaline	151 180	3	3	0.40	3 520	1 623	1 897	2.33%	46.11%
15	Low saltern (Pond 11) viruses	Hypersaline	268 049	9	21	1.12	6 847	3 374	3 473	2.55%	49.28%
16	Low saltern (Pond 11) viruses (LP1110)	Hypersaline	109 836	0	1	0.09	2 239	1 195	1 044	2.04%	53.37%
17	Medium saltern viruses (Pond MP1110)	Hypersaline	39 348	0	0	0.00	893	463	430	2.27%	51.85%
18	Medium saltern viruses (MP1122)	Hypersaline	55 142	3	0	0.54	865	513	352	1.57%	59.31%
19	High saltern viruses (HP1207)	Hypersaline	46 628	5	4	1.93	2 606	274	2 332	5.59%	10.51%
20	High saltern viral (HP1128)	Hypersaline	4 536	2	22	52.91	752	98	654	16.58%	13.03%
21	Low saltern viruses (LP1128)	Hypersaline	62 363	63	112	28.06	10 517	997	9 520	16.86%	9.48%
22	Salton Sea Phage 1	Hypersaline	55 467	0	0	0.00	959	546	413	1.73%	56.93%
23	Salton Sea Phage 3	Hypersaline	29 814	0	0	0.00	464	242	222	1.56%	52.16%
32	Marine Phages GOM	Seawater	262 501	29	50	3.01	30 214	2 789	27 425	11.51%	9.23%
33	Marine Phages BBC	Seawater	414 964	27	61	2.12	21 048	3 762	17 286	5.07%	17.87%
34	Marine Phages Arctic	Seawater	686 209	162	231	5.73	202 106	12 898	189 208	29.45%	6.38%
35	Marine Phage SAR	Seawater	397 939	7	18	0.63	7 539	2 211	5 328	1.89%	29.33%
36	Line Islands Kingman Reef B2 phage	Seawater	93 744	0	8	0.85	5 477	513	4 964	5.84%	9.37%
37	Line Islands Christmas Reef B3 phage	Seawater	279 882	52	103	5.54	63 629	2 869	60 760	22.73%	4.51%
38	Line Islands Palmyra F8 Phage	Seawater	318 178	0	13	0.41	4 079	1 273	2 806	1.28%	31.21%
39	Line Islands Tabuaeran B1 Phage	Seawater	378 475	0	0	0.00	4 533	1 289	3 244	1.20%	28.44%
40	Tampa Bay phage from induction experiment	Seawater	279 129	0	0	0.00	5 080	2 476	2 604	1.82%	48.74%
41	Skan Bay Phage 1	Seawater	30 831	0	0	0.00	476	209	267	1.54%	43.91%
46	Tpond phage 3	Freshwater	56 549	0	6	1.06	1 274	810	464	2.25%	63.58%
47	Healthy Tilapia pond phages	Freshwater	60 135	2	0	0.33	1 352	853	499	2.25%	63.09%
48	Healthy Prebead tank phages	Freshwater	67 785	2	5	1.03	2 058	1 377	681	3.04%	66.91%
49	Tilapia pond	Freshwater	264 844	2	9	0.42	9 674	5 999	3 675	3.65%	62.01%
57	Porites compressa time zero viruses	Coral	39 113	2	10	3.07	2412	207	2 205	6.17%	8.58%
58	Porites compressa control treated viruses	Coral	39 191	144	33	45.16	6191	792	5 399	15.80%	12.79%
59	Porites compressa DOC treated viruses	Coral	35 409	18	52	19.77	2126	334	1 792	6.00%	15.71%
60	Porites compressa pH treated viruses	Coral	49 949	2	31	6.61	2505	386	2 119	5.02%	15.41%
61	Porites compressa nutrient treated viruses	Coral	34 139	1	20	6.15	2639	376	2 263	7.73%	14.25%
62	Porites compressa temperature treated viruses	Coral	38 482	16	38	14.03	1 911	196	1 715	4.97%	10.26%
66	Pozas Azules Stromatolites phages	Microbialites	301 264	4	7	0.37	4 361	1 592	2 769	1.45%	36.51%
67	Rios Mesquites Stromatolites phages	Microbialites	324 500	4	8	0.37	11 966	10 158	1 808	3.69%	84.89%
68	Highborne Cay Stromatolite phage	Microbialites	148 334	0	0	0.00	466	184	282	0.31%	39.48%
73	Healthy slime viruses	Fish	61 022	22	89	18.19	12 924	877	12 047	21.18%	6.79%
74	Morbid slime viruses	Fish	59 599	25	82	17.95	20 446	1 194	19 252	34.31%	5.84%
83	CF Lung Sputum Viruses	Human Lung	92 223	6	10	1.73	1 331	129	1 202	1.44%	9.69%
84	Health Lung Sputum Viruses	Human Lung	39 489	0	3	0.76	292	24	268	0.74%	8.22%
85	Mosquito DNA 1	Mosquito	336 760	61	63	3.68	87 878	18 256	69 622	26.10%	20.77%
86	Mosquito DNA 2	Mosquito	638 689	133	114	3.87	257 424	19 480	237 944	40.31%	7.57%
87	Mosquito DNA 3	Mosquito	601 040	162	228	6.49	178 516	20 504	158 012	29.70%	11.49%
AISp	Lake Limnopolar Spring	Freshwater	41 322	0	2	0.48	640	311	329	0.24%	29.00%
AISu	Lake Limnopolar Summer	Freshwater	38 475	1	2	0.78	4 457	786	3 671	3.14%	11.52%
MET6	Lake Pavin	Freshwater	649 290	0	0	0.00	41 460	25 795	15 665	0.26%	62.42%
MET7	Lake Bourget	Freshwater	593 084	0	0	0.00	96 717	58 217	38 500	0.62%	54.42%
SRR043421	Human Gut L1 8	Human gut	30 873	4	8	3.89	14 767	5 859	8 908	15.77%	23.63%
SRR043422	Human Gut L2 1	Human gut	132 569	13	12	1.89	21 405	14 266	7 139	2.83%	60.41%
SRR043423	Human Gut L2 7	Human gut	61 104	1	2	0.49	6 069	3 203	2 866	1.47%	52.73%
SRR043424	Human Gut L2 8	Human gut	148 781	8	9	1.14	35 356	22 808	12 548	2.02%	58.79%
SRR043425	Human Gut H1 7	Human gut	16 955	1	2	1.77	3 692	2 247	1 445	2.11%	57.98%
SRR043426	Human Gut H1 8	Human gut	13 048	0	1	0.77	5 298	3 524	1 774	3.56%	56.34%
SRR043427	Human Gut H2 8	Human gut	16 747	0	1	0.60	4 763	3 083	1 680	2.97%	58.43%
SRR043428	Human Gut L1 1	Human gut	16 137	0	1	0.62	3 429	2 247	1 182	2.21%	60.78%
SRR043430	Human Gut H1 2	Human gut	107 259	2	12	1.31	39 254	27 821	11 433	3.70%	55.44%
SRR043431	Human Gut H1 1	Human gut	107 993	3	3	0.56	34 802	24 199	10 603	3.02%	65.28%
SRR043432	Human Gut H2 1	Human gut	25 648	0	2	0.78	5 999	3 731	2 268	2.64%	42.18%
SRR043433	Human Gut L3 1	Human gut	23 614	0	1	0.42	2 785	2 027	758	1.58%	70.43%
SRR043434	Human Gut L3 2	Human gut	33 489	1	0	0.30	4 785	3 281	1 504	1.59%	56.87%
SRR043435	Human Gut L3 7	Human gut	76 090	3	8	1.45	14 467	7 963	6 504	3.74%	29.84%
SRR043436	Human Gut L3 8	Human gut	15 166	1	0	0.66	2 722	1 661	1 061	2.29%	42.36%
SRR043437	Human Gut F-A	Human gut	59 155	2	5	1.18	10 975	6 311	4 664	4.41%	34.28%
SRR089800	Human Gut F-B	Human gut	113 054	9	0	0.80	6 308	3 504	2 804	0.25%	59.44%
SRR089802	Human Gut F-C	Human gut	109 569	3	1	0.37	11 199	5 706	5 493	0.88%	63.86%
SRR089803	Human Gut F-D	Human gut	68 391	0	3	0.44	8 597	5 614	2 983	0.55%	61.66%
SRR089804	Human Gut F-E	Human gut	115 121	1	3	0.35	9 733	5 397	4 336	0.91%	34.45%
SRR089805	Human Gut Unknown	Human gut	98 511	9	5	1.42	5 481	3 384	2 097	0.45%	50.91%

Microbiome Id	Name	Biome	Total number of sequences	Number of 16S rDNA detected	Number of 23S rDNA detected	rDNA ‰	Total number of hits for 10 000 reads	Number of hits in prophage-like region	Number of hits in non-prophage region	Microbial Hit Ratio	Prophage Hit Ratio
1	Soudan Red Stuff	Subterranean	334 386	236	642	26.26	1 230	103	1 127	12.30%	8.37%
2	Soudan Black Stuff	Subterranean	388 627	19	23	1.08	219	21	198	2.19%	9.59%
3	Low saltern microbes (Pond 11)	Hypersaline	268 206	196	359	20.69	1 425	83	1 342	14.25%	5.82%
4	Medium saltern microbes (MB1110)	Hypersaline	38 929	43	71	29.28	1 882	130	1 752	18.82%	6.91%
5	Medium saltern microbes (MB1111)	Hypersaline	23 261	37	48	36.54	1 938	118	1 820	19.38%	6.09%
6	Low saltern pond plasmids (TT)	Hypersaline	111 431	117	179	26.56	1 205	70	1 135	12.05%	5.81%
7	Medium saltern microbial (MB1128)	Hypersaline	8 062	5	12	21.09	508	33	475	6.30%	6.50%
8	High saltern microbial (HB1128)	Hypersaline	35 446	14	41	15.52	2 321	188	2 133	23.21%	8.10%
9	Salton Sea Bacteria 1	Hypersaline	178 407	47	80	7.12	716	55	661	7.16%	7.68%
10	Medium salinity microbial (MB1116)	Hypersaline	120 987	134	221	29.34	2 171	167	2 004	21.71%	7.69%
11	Low salinity microbial (LB1128)	Hypersaline	34 296	27	41	19.83	595	28	567	5.95%	4.71%
24	Line Islands Kingman Reef B2 bacteria	Marine	188 445	11	23	1.80	334	15	319	3.34%	4.49%
25	Line Islands Christmas Reef B3 bacteria	Marine	227 542	18	42	2.64	192	14	178	1.92%	7.29%
26	Line Islands Palmyra F8 Bacteria	Marine	289 723	35	102	4.73	604	49	555	6.04%	8.11%
27	Line Islands Tabuaeran B1 Bacteria	Marine	290 844	4	48	1.79	200	28	172	2.00%	14.00%
28	DMSP 1 (MAM.1)	Marine	54 848	84	88	31.36	1 513	84	1 429	15.13%	5.55%
29	DMSP 2 (MAM.2)	Marine	50 313	88	112	39.75	962	53	909	9.62%	5.51%
30	VAN 1 (MAM 3)	Marine	12 446	17	19	28.92	908	67	841	9.08%	7.38%
31	VAN 2 (MAM 4)	Marine	33 773	39	81	35.53	1 317	91	1 226	13.17%	6.91%
42	Tilapia pond microbes	Freshwater	381 076	130	325	11.94	1 265	107	1 158	12.65%	8.46%
43	Healthy Tilapia pond microbes	Freshwater	63 978	46	77	19.23	1 143	92	1 051	11.43%	8.05%
44	Healthy Prebead tank microbes	Freshwater	44 094	25	51	17.24	1 368	107	1 261	13.68%	7.82%
45	Tpond microbe 3	Freshwater	67 612	62	122	27.21	1 294	89	1 205	12.94%	6.88%
50	Porites compressa time zero bacteria	Coral	53 473	2	17	3.55	144	20	124	1.44%	13.89%
51	Porites compressa control treated bacteria	Coral	65 191	4	19	3.53	111	14	97	1.11%	12.61%
52	Porites compressa temperature treated bacteria	Coral	61 356	17	18	5.70	91	15	76	0.91%	16.48%
53	Porites compressa DOC treated microbes	Coral	62 959	9	23	5.08	82	13	69	0.82%	15.85%
54	Porites compressa pH treated microbes	Coral	67 994	21	31	7.65	108	14	94	1.08%	12.96%
55	Porites compressa nutrient treated microbes	Coral	65 008	13	46	9.08	200	27	173	2.00%	13.50%
56	Porites astreoides microbial extraction	Coral	316 279	348	952	41.10	232	13	219	2.32%	5.60%
63	Rios Mesquites Stromatolites bacteria	Microbialites	124 694	11	16	2.17	1 881	82	1 799	18.81%	4.36%
64	Highborne Cay stromatolite bacteria	Microbialites	257 573	7	2	0.35	102	52	50	1.02%	50.98%
65	Pozas Azule II stromatolite microbes	Microbialites	326 146	45	130	5.37	833	75	758	8.33%	9.00%
69	Healthy slime bacteria	Fish	66 066	53	165	33.00	2 442	167	2 275	24.42%	6.84%
70	Morbid slime bacteria	Fish	82 442	105	345	54.58	2 701	194	2 507	27.01%	7.18%
71	Healthy gut bacteria	Fish	51 498	48	144	37.28	3 752	181	3 571	37.52%	4.82%
72	Morbid gut bacteria	Fish	60 311	65	229	48.75	3 696	459	3 237	36.96%	12.42%
75	Pooled Planktonic	Cow rumen	236 830	292	502	33.53	1 596	155	1 441	15.96%	9.71%
76	80F6	Cow rumen	178 713	224	398	34.80	1 754	166	1 588	17.54%	9.46%
77	640F6	Cow rumen	264 849	345	554	33.94	1 374	134	1 240	13.74%	9.75%
78	710F6	Cow rumen	345 317	645	1179	52.82	3 272	282	2 990	32.72%	8.62%
79	Lean Mice	Mice	49 074	31	53	17.12	8 245	1 414	6 831	82.45%	17.15%
80	Obese Mice	Mice	35 053	28	33	17.40	7 956	1 613	6 343	79.56%	20.27%
81	Chicken Cecum B	Chicken cecum	237 940	390	678	44.89	2 090	294	1 796	20.90%	14.07%
82	Chicken Cecum A	Chicken cecum	294 682	453	824	43.33	3 248	443	2 805	32.48%	13.64%

Table S3 : **Table of virome-genome association** where more than 500 reads from the virome were found to be similar to a non-prophage region of the genome. For each virome-genome pair, the number of reads with a hit is indicated, as well as the evaluation of the scattering of the reads from a recruitment plot (X : near complete coverage of the genome, - : coverage restricted to specific regions, ? : too few hits to assess), the percentage and number of genes covered by virome reads, and the presence of a GTA cluster in the genome,. Virome are colored as in Fig 1. All recruitment plots are available on http://metavir-meb.univ-bpclermont.fr/Recruitment_plots/recruitment_plot_gallery.php

Genome	Virome	Biome	Hit number	Cover – Plot	% Gene	Nb Genes	GTA
vok	MET7	Freshwater	1744	-	1.02	978	-
ajs	SRR089804	Eukaryote	2055	-	0.07	4383	-
pub	36	Marine	1973	X	56.23	1389	-
amc	21	Marine	1085	X	19.27	4146	-
sal	32	Marine	12435	X	70.71	3260	X
mlo	32	Marine	2028	X	16.38	7333	-
sal	33	Marine	6335	X	60.40	3260	X
mlo	33	Marine	1592	X	14.17	7333	-
sal	34	Marine	91315	X	79.82	3260	X
mlo	34	Marine	11602	X	33.68	7333	-
nar.pNL1	34	Marine	5954	X	47.59	187	X
eli	34	Marine	5647	X	14.78	3059	X
nar	34	Marine	4203	X	15.88	4031	X
swi	34	Marine	3981	X	14.12	5455	-
mes	34	Marine	3715	X	27.80	4684	-
hdn	34	Marine	2186	X	27.06	3600	-
pub	37	Marine	34799	X	91.36	1389	-
hse	73	Eukaryote	7162	X	47.17	4804	-
hse	74	Eukaryote	10548	X	55.25	4804	-
sbp	85	Eukaryote	19568	X	69.48	4666	-
sbn	85	Eukaryote	10004	X	52.83	4859	-
shw	85	Eukaryote	5748	X	40.60	4217	-
sbm	85	Eukaryote	5730	X	39.06	4596	-
son	85	Eukaryote	1821	?	9.80	4745	-
spe	86	Eukaryote	54182	X	65.50	5064	-
ssn	86	Eukaryote	15864	-	5.81	4804	-
pak	86	Eukaryote	12868	?	33.36	4956	-
sbc	86	Eukaryote	9600	-	0.39	5323	-
pau	86	Eukaryote	9345	-	21.67	5977	-
ecj	86	Eukaryote	9224	-	0.89	4494	-
sdy	86	Eukaryote	8047	-	0.80	4892	-
cpi	86	Eukaryote	6076	X	15.62	7399	-
mpo	86	Eukaryote	5237	?	11.22	5546	X
ecr	86	Eukaryote	3371	-	0.24	4635	-
ypy	86	Eukaryote	3365	X	21.26	4313	-
mea	86	Eukaryote	2559	-	7.63	6345	X
sty.pHCM1	86	Eukaryote	2539	-	2.85	246	-
yen	86	Eukaryote	2227	?	17.57	4280	-
pap	86	Eukaryote	1909	-	9.73	6369	-
swi	86	Eukaryote	1762	-	6.58	5455	-
pac	86	Eukaryote	1675	-	16.09	2368	-
mdi	86	Eukaryote	1665	-	4.46	6030	X
dia	86	Eukaryote	1660	?	7.20	3609	-
sjp.1	86	Eukaryote	1530	?	8.63	4461	X
pak	87	Eukaryote	22945	?	52.66	2410	-
pfs	87	Eukaryote	18589	X	61.80	6584	-
spe	87	Eukaryote	10150	X	45.70	5064	-
pac	87	Eukaryote	6797	-	30.32	2368	-
cpi	87	Eukaryote	1590	X	8.74	7399	-
bth	SRR043421	Eukaryote	3659	X	36.70	4902	-

Table S4 : **Detection of putative GTA clusters in KEGG genomes**. Each line describes a region considered as a putative GTA cluster (complete or fragmented), and displays the first and last gene of the region, the number of GTA-associated genes, number of viral-associated genes, and the taxonomic affiliation of the species involved.

gene_start	gene_stop	nb_genes	nb_GTA_genes	nb_viral_genes	species	kingdom	class	genus	species
rfe:RF_0311	rfe:RF_0318	8	1	2	rfe	Bacteria	Alphaproteobacteria	Rickettsia	Rickettsia felis
rfe:RF_0471	rfe:RF_0471	1	1	1	rfe	Bacteria	Alphaproteobacteria	Rickettsia	Rickettsia felis
rfe:RF_0479	rfe:RF_0479	1	1	1	rfe	Bacteria	Alphaproteobacteria	Rickettsia	Rickettsia felis
rfe:RF_0745	rfe:RF_0759	15	3	4	rfe	Bacteria	Alphaproteobacteria	Rickettsia	Rickettsia felis
rbe:RBE_0303	rbe:RBE_0311	9	3	4	rbe	Bacteria	Alphaproteobacteria	Rickettsia	Rickettsia bellii RML369-C
rbe:RBE_0629	rbe:RBE_0638	10	1	4	rbe	Bacteria	Alphaproteobacteria	Rickettsia	Rickettsia bellii RML369-C
rbe:RBE_0759	rbe:RBE_0759	1	1	1	rbe	Bacteria	Alphaproteobacteria	Rickettsia	Rickettsia bellii RML369-C
rbo:A1I_04355	rbo:A1I_04420	12	1	4	rbo	Bacteria	Alphaproteobacteria	Rickettsia	Rickettsia bellii OSU 85-389
rbo:A1I_04905	rbo:A1I_04905	1	1	1	rbo	Bacteria	Alphaproteobacteria	Rickettsia	Rickettsia bellii OSU 85-389
rbo:A1I_06250	rbo:A1I_06285	8	3	3	rbo	Bacteria	Alphaproteobacteria	Rickettsia	Rickettsia bellii OSU 85-389
ott:OTT_0461	ott:OTT_0468	8	1	3	ott	Bacteria	Alphaproteobacteria	Orientia	Orientia tsutsugamushi Ikeda
ott:OTT_0894	ott:OTT_0904	11	3	5	ott	Bacteria	Alphaproteobacteria	Orientia	Orientia tsutsugamushi Ikeda
ott:OTT_1321	ott:OTT_1321	1	1	1	ott	Bacteria	Alphaproteobacteria	Orientia	Orientia tsutsugamushi Ikeda
wol:WD0379	wol:WD0383	5	1	2	wol	Bacteria	Alphaproteobacteria	Wolbachia	Wolbachia wMel
wol:WD0443	wol:WD0449	6	1	2	wol	Bacteria	Alphaproteobacteria	Wolbachia	Wolbachia wMel
wol:WD0458	wol:WD0458	1	1	1	wol	Bacteria	Alphaproteobacteria	Wolbachia	Wolbachia wMel
wol:WD0744	wol:WD0749	7	1	1	wol	Bacteria	Alphaproteobacteria	Wolbachia	Wolbachia wMel
wol:WD1012	wol:WD1012	1	1	1	wol	Bacteria	Alphaproteobacteria	Wolbachia	Wolbachia wMel
wol:WD1311	wol:WD1311	1	1	1	wol	Bacteria	Alphaproteobacteria	Wolbachia	Wolbachia wMel
wri:WRi_002750	wri:WRi_002750	1	1	1	wri	Bacteria	Alphaproteobacteria	Wolbachia	Wolbachia sp. wRi
wri:WRi_002970	wri:WRi_003020	6	1	3	wri	Bacteria	Alphaproteobacteria	Wolbachia	Wolbachia sp. wRi
wri:WRi_004090	wri:WRi_004090	1	1	1	wri	Bacteria	Alphaproteobacteria	Wolbachia	Wolbachia sp. wRi
wri:WRi_007300	wri:WRi_007300	1	1	1	wri	Bacteria	Alphaproteobacteria	Wolbachia	Wolbachia sp. wRi
wri:WRi_009700	wri:WRi_009700	1	1	1	wri	Bacteria	Alphaproteobacteria	Wolbachia	Wolbachia sp. wRi
wri:WRi_013380	wri:WRi_013380	1	1	1	wri	Bacteria	Alphaproteobacteria	Wolbachia	Wolbachia sp. wRi
ama:AM397	ama:AM406	8	3	5	ama	Bacteria	Alphaproteobacteria	Anaplasma	Anaplasma marginale St. Maries
ama:AM809	ama:AM809	1	1	1	ama	Bacteria	Alphaproteobacteria	Anaplasma	Anaplasma marginale St. Maries
ama:AM1304	ama:AM1305	2	2	2	ama	Bacteria	Alphaproteobacteria	Anaplasma	Anaplasma marginale St. Maries
amf:AMF_293	amf:AMF_296	4	3	3	amf	Bacteria	Alphaproteobacteria	Anaplasma	Anaplasma marginale Florida
amf:AMF_601	amf:AMF_601	1	1	1	amf	Bacteria	Alphaproteobacteria	Anaplasma	Anaplasma marginale Florida
amf:AMF_985	amf:AMF_986	2	2	2	amf	Bacteria	Alphaproteobacteria	Anaplasma	Anaplasma marginale Florida
acn:ACIS_00100	acn:ACIS_00101	2	2	2	acn	Bacteria	Alphaproteobacteria	Anaplasma	Anaplasma centrale
acn:ACIS_00531	acn:ACIS_00531	1	1	1	acn	Bacteria	Alphaproteobacteria	Anaplasma	Anaplasma centrale
acn:ACIS_00894	acn:ACIS_00899	4	3	3	acn	Bacteria	Alphaproteobacteria	Anaplasma	Anaplasma centrale
aph:APH_0022	aph:APH_0023	2	2	2	aph	Bacteria	Alphaproteobacteria	Anaplasma	Anaplasma phagocytophilum
aph:APH_0091	aph:APH_0091	1	1	1	aph	Bacteria	Alphaproteobacteria	Anaplasma	Anaplasma phagocytophilum
aph:APH_0378	aph:APH_0378	1	1	1	aph	Bacteria	Alphaproteobacteria	Anaplasma	Anaplasma phagocytophilum
aph:APH_0680	aph:APH_0688	9	2	2	aph	Bacteria	Alphaproteobacteria	Anaplasma	Anaplasma phagocytophilum
aph:APH_0861	aph:APH_0861	1	1	1	aph	Bacteria	Alphaproteobacteria	Anaplasma	Anaplasma phagocytophilum
eru:Erum0200	eru:Erum0210	2	2	2	eru	Bacteria	Alphaproteobacteria	Ehrlichia	Ehrlichia ruminantium Welgevonden (South Africa)
eru:Erum2630	eru:Erum2660	4	3	3	eru	Bacteria	Alphaproteobacteria	Ehrlichia	Ehrlichia ruminantium Welgevonden (South Africa)
eru:Erum5190	eru:Erum5230	5	1	2	eru	Bacteria	Alphaproteobacteria	Ehrlichia	Ehrlichia ruminantium Welgevonden (South Africa)
erw:ERWE_CDS_00070	erw:ERWE_CDS_00080	2	2	2	erw	Bacteria	Alphaproteobacteria	Ehrlichia	Ehrlichia ruminantium Welgevonden (France)
erw:ERWE_CDS_02670	erw:ERWE_CDS_02700	4	3	3	erw	Bacteria	Alphaproteobacteria	Ehrlichia	Ehrlichia ruminantium Welgevonden (France)
erw:ERWE_CDS_05440	erw:ERWE_CDS_05500	7	1	2	erw	Bacteria	Alphaproteobacteria	Ehrlichia	Ehrlichia ruminantium Welgevonden (France)
erg:ERGA_CDS_00070	erg:ERGA_CDS_00080	2	2	2	erg	Bacteria	Alphaproteobacteria	Ehrlichia	Ehrlichia ruminantium Gardel
erg:ERGA_CDS_02630	erg:ERGA_CDS_02660	4	3	3	erg	Bacteria	Alphaproteobacteria	Ehrlichia	Ehrlichia ruminantium Gardel
erg:ERGA_CDS_05340	erg:ERGA_CDS_05390	6	1	2	erg	Bacteria	Alphaproteobacteria	Ehrlichia	Ehrlichia ruminantium Gardel
ecn:Ecaj_0012	ecn:Ecaj_0013	2	2	2	ecn	Bacteria	Alphaproteobacteria	Ehrlichia	Ehrlichia canis
ecn:Ecaj_0253	ecn:Ecaj_0256	4	3	3	ecn	Bacteria	Alphaproteobacteria	Ehrlichia	Ehrlichia canis
ecn:Ecaj_0528	ecn:Ecaj_0528	1	1	1	ecn	Bacteria	Alphaproteobacteria	Ehrlichia	Ehrlichia canis
ech:ECH_0032	ech:ECH_0033	2	2	2	ech	Bacteria	Alphaproteobacteria	Ehrlichia	Ehrlichia chaffeensis
ech:ECH_0500	ech:ECH_0500	1	1	1	ech	Bacteria	Alphaproteobacteria	Ehrlichia	Ehrlichia chaffeensis
ech:ECH_0830	ech:ECH_0830	1	1	1	ech	Bacteria	Alphaproteobacteria	Ehrlichia	Ehrlichia chaffeensis
ech:ECH_0835	ech:ECH_0836	2	2	2	ech	Bacteria	Alphaproteobacteria	Ehrlichia	Ehrlichia chaffeensis
pla:Plav_0902	pla:Plav_0923	22	12	13	pla	Bacteria	Alphaproteobacteria	Parvibaculum	Parvibaculum lavamentivorans
atu:Atu0948	atu:Atu8142	23	9	9	atu	Bacteria	Alphaproteobacteria	Agrobacterium	Agrobacterium tumefaciens C58
avi:Avi_1329	avi:Avi_1357	21	13	12	avi	Bacteria	Alphaproteobacteria	Agrobacterium	Agrobacterium vitis S4
bme:BMEI1338	bme:BMEI1350	13	9	7	bme	Bacteria	Alphaproteobacteria	Brucella	Brucella melitensis bv. 1 16M
bmi:BMEA_A0624	bmi:BMEA_A0641	18	10	9	bmi	Bacteria	Alphaproteobacteria	Brucella	Brucella melitensis ATCC 23457
bmf:BAB1_0608	bmf:BAB1_0627	20	10	9	bmf	Bacteria	Alphaproteobacteria	Brucella	Brucella melitensis biovar Abortus
bmb:BruAb1_0605	bmb:BruAb1_0622	18	9	9	bmb	Bacteria	Alphaproteobacteria	Brucella	Brucella abortus 9-941
bmc:BAbS19_I05690	bmc:BAbS19_I05870	18	11	10	bmc	Bacteria	Alphaproteobacteria	Brucella	Brucella abortus S19
bms:BR0584	bms:BR0603	20	11	10	bms	Bacteria	Alphaproteobacteria	Brucella	Brucella suis 1330
bmt:BSUIS_A0613	bmt:BSUIS_A0633	19	10	9	bmt	Bacteria	Alphaproteobacteria	Brucella	Brucella suis ATCC 23445
bov:BOV_0584	bov:BOV_0602	19	8	8	bov	Bacteria	Alphaproteobacteria	Brucella	Brucella ovis
bcs:BCAN_A0598	bcs:BCAN_A0618	20	11	10	bcs	Bacteria	Alphaproteobacteria	Brucella	Brucella canis
bmr:BMI_I583	bmr:BMI_I602	20	12	10	bmr	Bacteria	Alphaproteobacteria	Brucella	Brucella microti
rpa:RPA1885	rpa:RPA1908	23	11	8	rpa	Bacteria	Alphaproteobacteria	Rhodopseudomonas	Rhodopseudomonas palustris CGA009
rpa:RPA1912	rpa:RPA1912	1	1	1	rpa	Bacteria	Alphaproteobacteria	Rhodopseudomonas	Rhodopseudomonas palustris CGA009
rpa:RPA1914	rpa:RPA1914	1	1	1	rpa	Bacteria	Alphaproteobacteria	Rhodopseudomonas	Rhodopseudomonas palustris CGA009
rpb:RPB_3455	rpb:RPB_3491	37	15	16	rpb	Bacteria	Alphaproteobacteria	Rhodopseudomonas	Rhodopseudomonas palustris HaA2
rpc:RPC_1797	rpc:RPC_1829	33	12	10	rpc	Bacteria	Alphaproteobacteria	Rhodopseudomonas	Rhodopseudomonas palustris BisB18
rpd:RPD_1964	rpd:RPD_1987	24	9	8	rpd	Bacteria	Alphaproteobacteria	Rhodopseudomonas	Rhodopseudomonas palustris BisB5
rpd:RPD_1991	rpd:RPD_1996	6	4	5	rpd	Bacteria	Alphaproteobacteria	Rhodopseudomonas	Rhodopseudomonas palustris BisB5
rpe:RPE_1893	rpe:RPE_1893	1	1	1	rpe	Bacteria	Alphaproteobacteria	Rhodopseudomonas	Rhodopseudomonas palustris BisA53
rpe:RPE_1895	rpe:RPE_1898	4	2	2	rpe	Bacteria	Alphaproteobacteria	Rhodopseudomonas	Rhodopseudomonas palustris BisA53
rpe:RPE_1902	rpe:RPE_1927	26	10	8	rpe	Bacteria	Alphaproteobacteria	Rhodopseudomonas	Rhodopseudomonas palustris BisA53
rpt:Rpal_2092	rpt:Rpal_2102	11	4	5	rpt	Bacteria	Alphaproteobacteria	Rhodopseudomonas	Rhodopseudomonas palustris TIE-1
rpt:Rpal_2106	rpt:Rpal_2116	11	7	4	rpt	Bacteria	Alphaproteobacteria	Rhodopseudomonas	Rhodopseudomonas palustris TIE-1
rpt:Rpal_2122	rpt:Rpal_2122	1	1	1	rpt	Bacteria	Alphaproteobacteria	Rhodopseudomonas	Rhodopseudomonas palustris TIE-1
rpt:Rpal_2124	rpt:Rpal_2124	1	1	1	rpt	Bacteria	Alphaproteobacteria	Rhodopseudomonas	Rhodopseudomonas palustris TIE-1

gene_start	gene_stop	nb_genes	nb_GTA_genes	nb_viral_genes	species	kingdom	class	genus	species
nwi:Nwi_1159	nwi:Nwi_1186	28	12	11	nwi	Bacteria	Alphaproteobacteria	Nitrobacter	Nitrobacter winogradskyi
nha:Nham_1408	nha:Nham_1415	8	4	4	nha	Bacteria	Alphaproteobacteria	Nitrobacter	Nitrobacter hamburgensis
nha:Nham_1419	nha:Nham_1432	14	7	7	nha	Bacteria	Alphaproteobacteria	Nitrobacter	Nitrobacter hamburgensis
oca:OCAR_6586	oca:OCAR_6597	12	7	6	oca	Bacteria	Alphaproteobacteria	Oligotropha	Oligotropha carboxidovorans
oca:OCAR_6601	oca:OCAR_6611	11	4	4	oca	Bacteria	Alphaproteobacteria	Oligotropha	Oligotropha carboxidovorans
xau:Xaut_3144	xau:Xaut_3150	7	2	3	xau	Bacteria	Alphaproteobacteria	Xanthobacter	Xanthobacter autotrophicus
xau:Xaut_3154	xau:Xaut_3164	11	5	4	xau	Bacteria	Alphaproteobacteria	Xanthobacter	Xanthobacter autotrophicus
xau:Xaut_3168	xau:Xaut_3175	8	4	6	xau	Bacteria	Alphaproteobacteria	Xanthobacter	Xanthobacter autotrophicus
azc:AZC_1105	azc:AZC_1105	1	1	1	azc	Bacteria	Alphaproteobacteria	Azorhizobium	Azorhizobium caulinodans
azc:AZC_1109	azc:AZC_1128	20	10	11	azc	Bacteria	Alphaproteobacteria	Azorhizobium	Azorhizobium caulinodans
azc:AZC_3029	azc:AZC_3029	1	1	1	azc	Bacteria	Alphaproteobacteria	Azorhizobium	Azorhizobium caulinodans
sno:Snov_0699	sno:Snov_0724	26	11	13	sno	Bacteria	Alphaproteobacteria	Starkeya	Starkeya novella
mex:Mext_1412	mex:Mext_1434	23	11	12	mex	Bacteria	Alphaproteobacteria	Methylobacterium	Methylobacterium extorquens
mea:Mex_1p1299	mea:Mex_1p1322	24	11	12	mea	Bacteria	Alphaproteobacteria	Methylobacterium	Methylobacterium extorquens AM1
mdi:METDI2070	mdi:METDI2094	25	11	12	mdi	Bacteria	Alphaproteobacteria	Methylobacterium	Methylobacterium extorquens DM4
mrd:Mrad2831_4959	mrd:Mrad2831_4965	7	4	4	mrd	Bacteria	Alphaproteobacteria	Methylobacterium	Methylobacterium radiotolerans
mrd:Mrad2831_4969	mrd:Mrad2831_4981	13	8	5	mrd	Bacteria	Alphaproteobacteria	Methylobacterium	Methylobacterium radiotolerans
mpo:Mpop_1408	mpo:Mpop_1421	14	6	7	mpo	Bacteria	Alphaproteobacteria	Methylobacterium	Methylobacterium populi
mpo:Mpop_1426	mpo:Mpop_1431	6	4	5	mpo	Bacteria	Alphaproteobacteria	Methylobacterium	Methylobacterium populi
mch:Mchl_1686	mch:Mchl_1709	24	11	12	mch	Bacteria	Alphaproteobacteria	Methylobacterium	Methylobacterium chloromethanicum
mno:Mnod_2003	mno:Mnod_2018	16	4	7	mno	Bacteria	Alphaproteobacteria	Methylobacterium	Methylobacterium nodulans
mno:Mnod_2593	mno:Mnod_2618	26	5	19	mno	Bacteria	Alphaproteobacteria	Methylobacterium	Methylobacterium nodulans
mno:Mnod_4275	mno:Mnod_4299	25	5	20	mno	Bacteria	Alphaproteobacteria	Methylobacterium	Methylobacterium nodulans
mno:Mnod_6557	mno:Mnod_6577	21	4	12	mno	Bacteria	Alphaproteobacteria	Methylobacterium	Methylobacterium nodulans
mno:Mnod_7308	mno:Mnod_7342	35	6	13	mno	Bacteria	Alphaproteobacteria	Methylobacterium	Methylobacterium nodulans
msl:Msil_2179	msl:Msil_2179	1	1	1	msl	Bacteria	Alphaproteobacteria	Methylocella	Methylocella silvestris
msl:Msil_3038	msl:Msil_3069	32	4	8	msl	Bacteria	Alphaproteobacteria	Methylocella	Methylocella silvestris
ccr:CC_2772	ccr:CC_2790	19	10	10	ccr	Bacteria	Alphaproteobacteria	Caulobacter	Caulobacter crescentus CB15
ccs:CCNA_02859	ccs:CCNA_02877	18	9	9	ccs	Bacteria	Alphaproteobacteria	Caulobacter	Caulobacter crescentus NA1000
ccs:CCNA_02880	ccs:CCNA_02880	1	1	1	ccs	Bacteria	Alphaproteobacteria	Caulobacter	Caulobacter crescentus NA1000
cak:Caul_3900	cak:Caul_3920	21	9	9	cak	Bacteria	Alphaproteobacteria	Caulobacter	Caulobacter sp. K31
cse:Cseg_0983	cse:Cseg_1002	20	9	9	cse	Bacteria	Alphaproteobacteria	Caulobacter	Caulobacter segnis
bsb:Bresu_1222	bsb:Bresu_1249	28	3	10	bsb	Bacteria	Alphaproteobacteria	Brevundimonas	Brevundimonas subvibrioides
bsb:Bresu_1255	bsb:Bresu_1273	19	6	6	bsb	Bacteria	Alphaproteobacteria	Brevundimonas	Brevundimonas subvibrioides
sil:SPO2249	sil:SPO2267	17	17	9	sil	Bacteria	Alphaproteobacteria	Ruegeria	Silicibacter pomeroyi
sit:TM1040_1056	sit:TM1040_1074	19	18	10	sit	Bacteria	Alphaproteobacteria	Ruegeria	Ruegeria sp. TM1040
rsp:RSP_2465	rsp:RSP_2480	18	16	9	rsp	Bacteria	Alphaproteobacteria	Rhodobacter	Rhodobacter sphaeroides 2.4.1
rsh:Rsph17029_1128	rsh:Rsph17029_1145	18	17	9	rsh	Bacteria	Alphaproteobacteria	Rhodobacter	Rhodobacter sphaeroides ATCC 17029
rsq:Rsph17025_1073	rsq:Rsph17025_1090	18	17	9	rsq	Bacteria	Alphaproteobacteria	Rhodobacter	Rhodobacter sphaeroides ATCC 17025
rsk:RSKD131_0781	rsk:RSKD131_0797	17	17	9	rsk	Bacteria	Alphaproteobacteria	Rhodobacter	Rhodobacter sphaeroides KD131
rcp:RCAP_rcc01682	rcp:RCAP_rcc01699	18	18	9	rcp	Bacteria	Alphaproteobacteria	Rhodobacter	Rhodobacter capsulatus
jan:Jann_1631	jan:Jann_1647	17	16	11	jan	Bacteria	Alphaproteobacteria	Jannaschia	Jannaschia sp. CCS1
rde:RD1_3016	rde:RD1_3035	17	16	9	rde	Bacteria	Alphaproteobacteria	Roseobacter	Roseobacter denitrificans
pde:Pden_2886	pde:Pden_2902	17	16	10	pde	Bacteria	Alphaproteobacteria	Paracoccus	Paracoccus denitrificans
dsh:Dshi_2162	dsh:Dshi_2179	18	15	10	dsh	Bacteria	Alphaproteobacteria	Dinoroseobacter	Dinoroseobacter shibae
mmr:Mmar10_0917	mmr:Mmar10_0934	18	9	9	mmr	Bacteria	Alphaproteobacteria	Maricaulis	Maricaulis maris
hba:Hbal_1246	hba:Hbal_1255	10	4	4	hba	Bacteria	Alphaproteobacteria	Hirschia	Hirschia baltica
hba:Hbal_1259	hba:Hbal_1268	10	6	5	hba	Bacteria	Alphaproteobacteria	Hirschia	Hirschia baltica
nar:Saro_3116	nar:Saro_3133	18	8	8	nar	Bacteria	Alphaproteobacteria	Novosphingobium	Novosphingobium aromaticivorans
sal:Sala_1989	sal:Sala_2005	17	8	9	sal	Bacteria	Alphaproteobacteria	Sphingopyxis	Sphingopyxis alaskensis
sjp:SJA_C1-12210	sjp:SJA_C1-12390	19	8	7	sjp	Bacteria	Alphaproteobacteria	Sphingobium	Sphingobium japonicum
eli:ELI_13955	eli:ELI_14045	19	9	8	eli	Bacteria	Alphaproteobacteria	Erythrobacter	Erythrobacter litoralis
pbr:PB2503_08004	pbr:PB2503_08139	28	9	11	pbr	Bacteria	Alphaproteobacteria	Parvularcula	Parvularcula bermudensis
bhy:BHWA1_01823	bhy:BHWA1_01856	24	24	1	bhy	Bacteria	Spirochaetes	Brachyspira	Brachyspira hyodysenteriae
brm:Bmur_1423	brm:Bmur_1441	19	13	1	brm	Bacteria	Spirochaetes	Brachyspira	Brachyspira murdochii
bpo:BP951000_1199	bpo:BP951000_1209	11	11	0	bpo	Bacteria	Spirochaetes	Brachyspira	Brachyspira pilosicoli
bpo:BP951000_1214	bpo:BP951000_1221	8	5	2	bpo	Bacteria	Spirochaetes	Brachyspira	Brachyspira pilosicoli
mfe:Mefer_1536	mfe:Mefer_1553	19	7	3	mfe	Archaea	Euryarchaeota	Methanocaldococcus	Methanocaldococcus fervens
mvo:Mvol_0401	mvo:Mvol_0415	15	15	4	mvo	Archaea	Euryarchaeota	Methanococcus	Methanococcus voltae

Table S5 : **Complete table of Kegg Orthologous groups retrieved in the viromes considered as free from any cellular DNA** (viromes with no rDNA detected).

Pathway	KO	Name	Definition	Class	Nb Reads	Nb Viromes
ko00010_Glycolysis / Gluconeogenesis						
	K00001	E1.1.1.1, adh	alcohol dehydrogenase [EC:1.1.1.1]	Carbohydrate Metabolism - Metabolism of Cofactors and Vitamins - Xenobiotics Biodegradation and Metabolism - Amino Acid Metabolism - Lipid Metabolism	1	1
	K00121	frmA, ADH5, adhC	S-(hydroxymethyl)glutathione dehydrogenase / alcohol dehydrogenase [EC:1.1.1.284 1.1.1.1]	Carbohydrate Metabolism - Metabolism of Cofactors and Vitamins - Xenobiotics Biodegradation and Metabolism - Amino Acid Metabolism - Lipid Metabolism - Energy Metabolism	8	3
	K00128	E1.2.1.3	aldehyde dehydrogenase (NAD+) [EC:1.2.1.3]	Carbohydrate Metabolism - Metabolism of Other Amino Acids - Xenobiotics Biodegradation and Metabolism - Metabolism of Terpenoids and Polyketides - Amino Acid Metabolism - Lipid Metabolism	4	4
	K00134	GAPDH, gapA	glyceraldehyde 3-phosphate dehydrogenase [EC:1.2.1.12]	Carbohydrate Metabolism - Neurodegenerative Diseases	3	2
	K00161	PDHA, pdhA	pyruvate dehydrogenase E1 component subunit alpha [EC:1.2.4.1]	Carbohydrate Metabolism - Amino Acid Metabolism	57	3
	K00162	PDHB, pdhB	pyruvate dehydrogenase E1 component subunit beta [EC:1.2.4.1]	Carbohydrate Metabolism - Amino Acid Metabolism	37	3
	K00382	DLD, lpd, pdhD	dihydrolipoamide dehydrogenase [EC:1.8.1.4]	Carbohydrate Metabolism - Amino Acid Metabolism	1	1
	K00627	DLAT, aceF, pdhC	pyruvate dehydrogenase E2 component (dihydrolipoamide acetyltransferase) [EC:2.3.1.12]	Carbohydrate Metabolism	1	1
	K00845	glk	glucokinase [EC:2.7.1.2]	Carbohydrate Metabolism - Biosynthesis of Other Secondary Metabolites	2	2
	K00927	PGK, pgk	phosphoglycerate kinase [EC:2.7.2.3]	Carbohydrate Metabolism - Energy Metabolism	2	1
	K01610	E4.1.1.49, pckA	phosphoenolpyruvate carboxykinase (ATP) [EC:4.1.1.49]	Carbohydrate Metabolism - Energy Metabolism	4	2
	K01623	ALDO, fbaB	fructose-bisphosphate aldolase, class I [EC:4.1.2.13]	Carbohydrate Metabolism - Energy Metabolism	12	1
	K01624	FBA, fbaA	fructose-bisphosphate aldolase, class II [EC:4.1.2.13]	Carbohydrate Metabolism - Energy Metabolism	2	1
	K01792	E5.1.3.15	glucose-6-phosphate 1-epimerase [EC:5.1.3.15]	Carbohydrate Metabolism	1	1
	K01810	GPI, pgi	glucose-6-phosphate isomerase [EC:5.3.1.9]	Carbohydrate Metabolism	2	2
	K01895	ACSS, acs	acetyl-CoA synthetase [EC:6.2.1.1]	Carbohydrate Metabolism - Lipid Metabolism - Energy Metabolism	1	1
	K02791	PTS-Mal-EIIC, malX	PTS system, maltose and glucose-specific IIC component	Carbohydrate Metabolism - Membrane Transport	1	1
	K04041	fbp3	fructose-1,6-bisphosphatase III [EC:3.1.3.11]	Carbohydrate Metabolism - Energy Metabolism	1	1
	K13953	adhP	alcohol dehydrogenase, propanol-preferring [EC:1.1.1.1]	Carbohydrate Metabolism - Metabolism of Cofactors and Vitamins - Xenobiotics Biodegradation and Metabolism - Amino Acid Metabolism - Lipid Metabolism	1	1
ko00020_Citrate cycle (TCA cycle)						
	K00024	mdh	malate dehydrogenase [EC:1.1.1.37]	Carbohydrate Metabolism - Energy Metabolism	3	3
	K00161	PDHA, pdhA	pyruvate dehydrogenase E1 component subunit alpha [EC:1.2.4.1]	Carbohydrate Metabolism - Amino Acid Metabolism	57	3
	K00162	PDHB, pdhB	pyruvate dehydrogenase E1 component subunit beta [EC:1.2.4.1]	Carbohydrate Metabolism - Amino Acid Metabolism	37	3
	K00164	OGDH, sucA	2-oxoglutarate dehydrogenase E1 component [EC:1.2.4.2]	Carbohydrate Metabolism - Amino Acid Metabolism	2	1
	K00174	korA	2-oxoglutarate ferredoxin oxidoreductase subunit alpha [EC:1.2.7.3]	Carbohydrate Metabolism - Energy Metabolism	1	1
	K00175	korB	2-oxoglutarate ferredoxin oxidoreductase subunit beta [EC:1.2.7.3]	Carbohydrate Metabolism - Energy Metabolism	2	2
	K00239	sdhA	succinate dehydrogenase flavoprotein subunit [EC:1.3.99.1]	Carbohydrate Metabolism - Xenobiotics Biodegradation and Metabolism - Energy Metabolism	2	2
	K00240	sdhB	succinate dehydrogenase iron-sulfur protein [EC:1.3.99.1]	Carbohydrate Metabolism - Xenobiotics Biodegradation and Metabolism - Energy Metabolism	18	1
	K00382	DLD, lpd, pdhD	dihydrolipoamide dehydrogenase [EC:1.8.1.4]	Carbohydrate Metabolism - Amino Acid Metabolism	1	1
	K00627	DLAT, aceF, pdhC	pyruvate dehydrogenase E2 component (dihydrolipoamide acetyltransferase) [EC:2.3.1.12]	Carbohydrate Metabolism	1	1
	K00658	DLST, sucB	2-oxoglutarate dehydrogenase E2 component (dihydrolipoamide succinyltransferase) [EC:2.3.1.61]	Carbohydrate Metabolism - Amino Acid Metabolism	2	2
	K01610	E4.1.1.49, pckA	phosphoenolpyruvate carboxykinase (ATP) [EC:4.1.1.49]	Carbohydrate Metabolism - Energy Metabolism	4	2
	K01681	ACO, acnA	aconitate hydratase 1 [EC:4.2.1.3]	Carbohydrate Metabolism - Energy Metabolism	4	3
ko00030_Pentose phosphate pathway						
	K00033	E1.1.1.44, PGD, gnd	6-phosphogluconate dehydrogenase [EC:1.1.1.44]	Carbohydrate Metabolism - Metabolism of Other Amino Acids	20	3
	K00036	G6PD, zwf	glucose-6-phosphate 1-dehydrogenase [EC:1.1.1.49]	Carbohydrate Metabolism - Metabolism of Other Amino Acids	22	2
	K00615	E2.2.1.1, tktA, tktB	transketolase [EC:2.2.1.1]	Carbohydrate Metabolism - Metabolism of Terpenoids and Polyketides - Energy Metabolism	59	3
	K00616	E2.2.1.2, talA, talB	transaldolase [EC:2.2.1.2]	Carbohydrate Metabolism	46	4
	K00852	E2.7.1.15, rbsK	ribokinase [EC:2.7.1.15]	Carbohydrate Metabolism	3	2
	K00874	kdgK	2-dehydro-3-deoxygluconokinase [EC:2.7.1.45]	Carbohydrate Metabolism	1	1
	K00948	PRPS, prsA	ribose-phosphate pyrophosphokinase [EC:2.7.6.1]	Carbohydrate Metabolism - Nucleotide Metabolism	9	6
	K01619	E4.1.2.4, deoC	deoxyribose-phosphate aldolase [EC:4.1.2.4]	Carbohydrate Metabolism	3	2
	K01623	ALDO, fbaB	fructose-bisphosphate aldolase, class I [EC:4.1.2.13]	Carbohydrate Metabolism - Energy Metabolism	12	1
	K01624	FBA, fbaA	fructose-bisphosphate aldolase, class II [EC:4.1.2.13]	Carbohydrate Metabolism - Energy Metabolism	2	1
	K01783	rpe, RPE	ribulose-phosphate 3-epimerase [EC:5.1.3.1]	Carbohydrate Metabolism - Energy Metabolism	1	1
	K01808	E5.3.1.6B, rpiB	ribose 5-phosphate isomerase B [EC:5.3.1.6]	Carbohydrate Metabolism - Energy Metabolism	10	3
	K01810	GPI, pgi	glucose-6-phosphate isomerase [EC:5.3.1.9]	Carbohydrate Metabolism	2	2
	K04041	fbp3	fructose-1,6-bisphosphatase III [EC:3.1.3.11]	Carbohydrate Metabolism - Energy Metabolism	1	1
ko00040_Pentose and glucuronate interconversions						
	K00012	UGDH, ugd	UDPglucose 6-dehydrogenase [EC:1.1.1.22]	Carbohydrate Metabolism	91	5
	K00041	uxaB	tagaturonate reductase [EC:1.1.1.58]	Carbohydrate Metabolism	1	1
	K00128	E1.2.1.3	aldehyde dehydrogenase (NAD+) [EC:1.2.1.3]	Carbohydrate Metabolism - Metabolism of Other Amino Acids - Xenobiotics Biodegradation and Metabolism - Metabolism of Terpenoids and Polyketides - Amino Acid Metabolism - Lipid Metabolism	4	4
	K00848	rhaB	rhamnulokinase [EC:2.7.1.5]	Carbohydrate Metabolism	1	1
	K00874	kdgK	2-dehydro-3-deoxygluconokinase [EC:2.7.1.45]	Carbohydrate Metabolism	1	1
	K01783	rpe, RPE	ribulose-phosphate 3-epimerase [EC:5.1.3.1]	Carbohydrate Metabolism - Energy Metabolism	1	1
	K01786	araD	L-ribulose-5-phosphate 4-epimerase [EC:5.1.3.4]	Carbohydrate Metabolism	1	1
	K01812	uxaC	glucuronate isomerase [EC:5.3.1.12]	Carbohydrate Metabolism	1	1
	K03082	sgbU	hexulose-6-phosphate isomerase [EC:5.-.-.-]	Carbohydrate Metabolism	1	1
ko00051_Fructose and mannose metabolism						
	K00008	E1.1.1.14, gutB	L-iditol 2-dehydrogenase [EC:1.1.1.14]	Carbohydrate Metabolism	3	2
	K00066	algD	GDP-mannose 6-dehydrogenase [EC:1.1.1.132]	Carbohydrate Metabolism - Signal Transduction	3	1
	K00100	E1.1.1.-		Carbohydrate Metabolism - Xenobiotics Biodegradation and Metabolism - Lipid Metabolism	1	1
	K00754	E2.4.1.-		Carbohydrate Metabolism	149	4
	K00847	E2.7.1.4, scrK	fructokinase [EC:2.7.1.4]	Carbohydrate Metabolism	1	1
	K00848	rhaB	rhamnulokinase [EC:2.7.1.5]	Carbohydrate Metabolism	1	1
	K00966	GMPP	mannose-1-phosphate guanylyltransferase [EC:2.7.7.13]	Carbohydrate Metabolism	4	2
	K00971	E2.7.7.22, manC	mannose-1-phosphate guanylyltransferase [EC:2.7.7.22]	Carbohydrate Metabolism	12	4
	K01623	ALDO, fbaB	fructose-bisphosphate aldolase, class I [EC:4.1.2.13]	Carbohydrate Metabolism - Energy Metabolism	12	1
	K01624	FBA, fbaA	fructose-bisphosphate aldolase, class II [EC:4.1.2.13]	Carbohydrate Metabolism - Energy Metabolism	2	1
	K01628	fucA	L-fuculose-phosphate aldolase [EC:4.1.2.17]	Carbohydrate Metabolism	1	1
	K01711	E4.2.1.47, gmd	GDPmannose 4,6-dehydratase [EC:4.2.1.47]	Carbohydrate Metabolism	533	7
	K01809	E5.3.1.8, manA	mannose-6-phosphate isomerase [EC:5.3.1.8]	Carbohydrate Metabolism	17	3
	K01840	E5.4.2.8, manB	phosphomannomutase [EC:5.4.2.8]	Carbohydrate Metabolism	1	1
	K02377	E1.1.1.271, fcl	GDP-L-fucose synthase [EC:1.1.1.271]	Carbohydrate Metabolism	251	7
	K02770	PTS-Fru-EIIC, fruA	PTS system, fructose-specific IIC component	Carbohydrate Metabolism - Membrane Transport	1	1
	K02795	PTS-Man-EIIC, manY	PTS system, mannose-specific IIC component	Carbohydrate Metabolism - Membrane Transport	1	1
	K04041	fbp3	fructose-1,6-bisphosphatase III [EC:3.1.3.11]	Carbohydrate Metabolism - Energy Metabolism	1	1
ko00052_Galactose metabolism						
	K00845	glk	glucokinase [EC:2.7.1.2]	Carbohydrate Metabolism - Biosynthesis of Other Secondary Metabolites	2	2
	K01187	E3.2.1.20, malZ	alpha-glucosidase [EC:3.2.1.20]	Carbohydrate Metabolism	1	1
	K01190	lacZ	beta-galactosidase [EC:3.2.1.23]	Carbohydrate Metabolism - Glycan Biosynthesis and Metabolism - Lipid Metabolism	1	1
	K01193	E3.2.1.26, sacA	beta-fructofuranosidase [EC:3.2.1.26]	Carbohydrate Metabolism	1	1
	K01684	E4.2.1.6, dgoAb	galactonate dehydratase [EC:4.2.1.6]	Carbohydrate Metabolism	2	2
	K01784	galE, GALE	UDP-glucose 4-epimerase [EC:5.1.3.2]	Carbohydrate Metabolism	175	8
ko00053_Ascorbate and aldarate metabolism						
	K00012	UGDH, ugd	UDPglucose 6-dehydrogenase [EC:1.1.1.22]	Carbohydrate Metabolism	91	5

K00128	E1.2.1.3	aldehyde dehydrogenase (NAD+) [EC:1.2.1.3]	Carbohydrate Metabolism - Metabolism of Other Amino Acids - Xenobiotics Biodegradation and Metabolism - Metabolism of Terpenoids and Polyketides - Amino Acid Metabolism - Lipid Metabolism	4	4
K03077	ulaF, sgaE	L-ribulose-5-phosphate 4-epimerase [EC:5.1.3.4]	Carbohydrate Metabolism	1	1
ko00061_Fatty acid biosynthesis					
K00059	fabG	3-oxoacyl-[acyl-carrier protein] reductase [EC:1.1.1.100]	Lipid Metabolism	10	4
K00647	fabB	3-oxoacyl-[acyl-carrier-protein] synthase I [EC:2.3.1.41]	Lipid Metabolism	6	2
K00648	fabH	3-oxoacyl-[acyl-carrier-protein] synthase III [EC:2.3.1.180]	Lipid Metabolism	6	3
K01962	accA	acetyl-CoA carboxylase carboxyl transferase subunit alpha [EC:6.4.1.2]	Carbohydrate Metabolism - Metabolism of Terpenoids and Polyketides - Lipid Metabolism - Energy Metabolism	1	1
K01963	accD	acetyl-CoA carboxylase carboxyl transferase subunit beta [EC:6.4.1.2]	Carbohydrate Metabolism - Metabolism of Terpenoids and Polyketides - Lipid Metabolism - Energy Metabolism	2	2
K02372	fabZ	3R-hydroxymyristoyl ACP dehydrase [EC:4.2.1.-]	Lipid Metabolism	1	1
K09458	fabF	3-oxoacyl-[acyl-carrier-protein] synthase II [EC:2.3.1.179]	Lipid Metabolism	4	2
K11263	bccA	acetyl-/propionyl-CoA carboxylase, biotin carboxylase, biotin carboxyl carrier protein [EC:6.3.4.14]	Lipid Metabolism	1	1
ko00071_Fatty acid metabolism					
K00001	E1.1.1.1, adh	alcohol dehydrogenase [EC:1.1.1.1]	Carbohydrate Metabolism - Metabolism of Cofactors and Vitamins - Xenobiotics Biodegradation and Metabolism - Amino Acid Metabolism - Lipid Metabolism	1	1
K00121	frmA, ADH5, adhC	S-(hydroxymethyl)glutathione dehydrogenase / alcohol dehydrogenase [EC:1.1.1.284 1.1.1.1]	Carbohydrate Metabolism - Metabolism of Cofactors and Vitamins - Xenobiotics Biodegradation and Metabolism - Amino Acid Metabolism - Lipid Metabolism - Energy Metabolism	8	3
K00128	E1.2.1.3	aldehyde dehydrogenase (NAD+) [EC:1.2.1.3]	Carbohydrate Metabolism - Metabolism of Other Amino Acids - Xenobiotics Biodegradation and Metabolism - Metabolism of Terpenoids and Polyketides - Amino Acid Metabolism - Lipid Metabolism	4	4
K00249	E1.3.99.3, ACADM, acd	acyl-CoA dehydrogenase [EC:1.3.99.3]	Carbohydrate Metabolism - Metabolism of Other Amino Acids - Endocrine System - Amino Acid Metabolism - Lipid Metabolism	1	1
K00529	hcaD	ferredoxin--NAD+ reductase [EC:1.18.1.3]	Xenobiotics Biodegradation and Metabolism - Amino Acid Metabolism - Lipid Metabolism	1	1
K00626	E2.3.1.9, atoB	acetyl-CoA C-acetyltransferase [EC:2.3.1.9]	Carbohydrate Metabolism - Signal Transduction - Xenobiotics Biodegradation and Metabolism - Metabolism of Terpenoids and Polyketides - Amino Acid Metabolism - Lipid Metabolism - Energy Metabolism	4	3
K01692	E4.2.1.17, paaG	enoyl-CoA hydratase [EC:4.2.1.17]	Carbohydrate Metabolism - Metabolism of Other Amino Acids - Xenobiotics Biodegradation and Metabolism - Metabolism of Terpenoids and Polyketides - Amino Acid Metabolism - Lipid Metabolism	33	3
K01825	fadB	3-hydroxyacyl-CoA dehydrogenase / enoyl-CoA hydratase / 3-hydroxybutyryl-CoA epimerase / enoyl-CoA isomerase [EC:1.1.1.35 4.2.1.17 5.1.2.3 5.3.3.8]	Carbohydrate Metabolism - Metabolism of Other Amino Acids - Xenobiotics Biodegradation and Metabolism - Metabolism of Terpenoids and Polyketides - Amino Acid Metabolism - Lipid Metabolism - Energy Metabolism	1	1
K01897	ACSL, fadD	long-chain acyl-CoA synthetase [EC:6.2.1.3]	Endocrine System - Transport and Catabolism - Lipid Metabolism	4	2
K05297	E1.18.1.1	rubredoxin-NAD+ reductase [EC:1.18.1.1]	Lipid Metabolism	1	1
K06445	fadE	acyl-CoA dehydrogenase [EC:1.3.99.-]	Lipid Metabolism	1	1
K13953	adhP	alcohol dehydrogenase, propanol-preferring [EC:1.1.1.1]	Carbohydrate Metabolism - Metabolism of Cofactors and Vitamins - Xenobiotics Biodegradation and Metabolism - Amino Acid Metabolism - Lipid Metabolism	1	1
ko00072_Synthesis and degradation of ketone bodies					
K00626	E2.3.1.9, atoB	acetyl-CoA C-acetyltransferase [EC:2.3.1.9]	Carbohydrate Metabolism - Signal Transduction - Xenobiotics Biodegradation and Metabolism - Metabolism of Terpenoids and Polyketides - Amino Acid Metabolism - Lipid Metabolism - Energy Metabolism	4	3
K01640	E4.1.3.4, HMGCL, hmgL	hydroxymethylglutaryl-CoA lyase [EC:4.1.3.4]	Carbohydrate Metabolism - Transport and Catabolism - Metabolism of Terpenoids and Polyketides - Amino Acid Metabolism - Lipid Metabolism	18	1
ko00130_Ubiquinone and other terpenoid-quinone biosynthesis					
K03179	ubiA	4-hydroxybenzoate octaprenyltransferase [EC:2.5.1.-]	Metabolism of Cofactors and Vitamins - Metabolism of Terpenoids and Polyketides	1	1
K03183	ubiE	ubiquinone/menaquinone biosynthesis methyltransferase [EC:2.1.1.163 2.1.1.-]	Metabolism of Cofactors and Vitamins	2	2
ko00190_Oxidative phosphorylation					
K00239	sdhA	succinate dehydrogenase flavoprotein subunit [EC:1.3.99.1]	Carbohydrate Metabolism - Xenobiotics Biodegradation and Metabolism - Energy Metabolism	2	2
K00240	sdhB	succinate dehydrogenase iron-sulfur protein [EC:1.3.99.1]	Carbohydrate Metabolism - Xenobiotics Biodegradation and Metabolism - Energy Metabolism	18	1
K00331	nuoB	NADH dehydrogenase I subunit B [EC:1.6.5.3]	Energy Metabolism	2	2
K00336	nuoG	NADH dehydrogenase I subunit G [EC:1.6.5.3]	Energy Metabolism	1	1
K00337	nuoH	NADH dehydrogenase I subunit H [EC:1.6.5.3]	Energy Metabolism	2	2
K00341	nuoL	NADH dehydrogenase I subunit L [EC:1.6.5.3]	Energy Metabolism	2	2
K00342	nuoM	NADH dehydrogenase I subunit M [EC:1.6.5.3]	Energy Metabolism	1	1
K00343	nuoN	NADH dehydrogenase I subunit N [EC:1.6.5.3]	Energy Metabolism	1	1
K00405	ccoO	cb-type cytochrome c oxidase subunit II [EC:1.9.3.1]	Energy Metabolism	1	1
K00412	CYTB, petB	ubiquinol-cytochrome c reductase cytochrome b subunit [EC:1.10.2.2]	Circulatory System - Neurodegenerative Diseases - Energy Metabolism	7	2
K00425	cydA	cytochrome bd-I oxidase subunit I [EC:1.10.3.-]	Energy Metabolism	15	1
K00426	cydB	cytochrome bd-I oxidase subunit II [EC:1.10.3.-]	Energy Metabolism	2	2
K01507	E3.6.1.1, ppa	inorganic pyrophosphatase [EC:3.6.1.1]	Energy Metabolism	3	2
K02109	ATPF0B, atpF	F-type H+-transporting ATPase subunit b [EC:3.6.3.14]	Energy Metabolism	2	2
K02111	ATPF1A, atpA	F-type H+-transporting ATPase subunit alpha [EC:3.6.3.14]	Energy Metabolism	4	2
K02118	ATPVB, ntpB	V-type H+-transporting ATPase subunit B [EC:3.6.3.14]	Energy Metabolism	1	1
K02274	coxA	cytochrome c oxidase subunit I [EC:1.9.3.1]	Energy Metabolism	7	2
K02301	cyoE	protoheme IX farnesyltransferase [EC:2.5.1.-]	Metabolism of Cofactors and Vitamins - Metabolism of Terpenoids and Polyketides - Energy Metabolism	2	2
K05572	ndhA	NADH dehydrogenase I subunit 1 [EC:1.6.5.3]	Energy Metabolism	1	1
K05580	ndhI	NADH dehydrogenase I subunit I [EC:1.6.5.3]	Energy Metabolism	66	1
ko00195_Photosynthesis					
K02109	ATPF0B, atpF	F-type H+-transporting ATPase subunit b [EC:3.6.3.14]	Energy Metabolism	2	2
K02111	ATPF1A, atpA	F-type H+-transporting ATPase subunit alpha [EC:3.6.3.14]	Energy Metabolism	4	2
K02638	petE	plastocyanin	Energy Metabolism	5	2
K02639	petF	ferredoxin	Energy Metabolism	10	2
K02641	petH	ferredoxin--NADP+ reductase [EC:1.18.1.2]	Energy Metabolism	1	1
K02689	psaA	photosystem I P700 chlorophyll a apoprotein A1	Energy Metabolism	52	1
K02690	psaB	photosystem I P700 chlorophyll a apoprotein A2	Energy Metabolism	50	1
K02691	psaC	photosystem I subunit VII	Energy Metabolism	10	1
K02692	psaD	photosystem I subunit II	Energy Metabolism	10	1
K02693	psaE	photosystem I subunit IV	Energy Metabolism	2	1
K02694	psaF	photosystem I subunit III	Energy Metabolism	4	1
K02697	psaJ	photosystem I subunit IX	Energy Metabolism	2	1
K02703	psbA	photosystem II P680 reaction center D1 protein	Energy Metabolism	334	6
K02704	psbB	photosystem II CP47 chlorophyll apoprotein	Energy Metabolism	1	1
K02705	psbC	photosystem II CP43 chlorophyll apoprotein	Energy Metabolism	6	2
K02706	psbD	photosystem II P680 reaction center D2 protein	Energy Metabolism	129	4
ko00230_Purine metabolism					
K00088	E1.1.1.205, guaB	IMP dehydrogenase [EC:1.1.1.205]	Nucleotide Metabolism - Xenobiotics Biodegradation and Metabolism	57	4
K00364	E1.7.1.7, guaC	GMP reductase [EC:1.7.1.7]	Nucleotide Metabolism	74	5
K00524	E1.17.4.1	ribonucleotide reductase, class II [EC:1.17.4.1]	Nucleotide Metabolism	3	2
K00525	E1.17.4.1A, nrdA, nrdE	ribonucleoside-diphosphate reductase alpha chain [EC:1.17.4.1]	Nucleotide Metabolism - Replication and Repair	3331	9
K00526	E1.17.4.1B, nrdB, nrdF	ribonucleoside-diphosphate reductase beta chain [EC:1.17.4.1]	Nucleotide Metabolism - Replication and Repair	869	8
K00527	nrdD	ribonucleoside-triphosphate reductase [EC:1.17.4.2]	Nucleotide Metabolism	57	7
K00759	E2.4.2.7, apt	adenine phosphoribosyltransferase [EC:2.4.2.7]	Nucleotide Metabolism	5	2
K00760	E2.4.2.8, hpt	hypoxanthine phosphoribosyltransferase [EC:2.4.2.8]	Nucleotide Metabolism - Xenobiotics Biodegradation and Metabolism	62	4
K00764	E2.4.2.14, purF	amidophosphoribosyltransferase [EC:2.4.2.14]	Enzyme Families - Nucleotide Metabolism - Amino Acid Metabolism	2	2
K00860	cysC	adenylylsulfate kinase [EC:2.7.1.25]	Nucleotide Metabolism - Energy Metabolism	165	4
K00939	E2.7.4.3, adk	adenylate kinase [EC:2.7.4.3]	Nucleotide Metabolism	15	3
K00940	E2.7.4.6, ndk	nucleoside-diphosphate kinase [EC:2.7.4.6]	Nucleotide Metabolism	2	1
K00942	E2.7.4.8, gmk	guanylate kinase [EC:2.7.4.8]	Nucleotide Metabolism	1	1
K00948	PRPS, prsA	ribose-phosphate pyrophosphokinase [EC:2.7.6.1]	Carbohydrate Metabolism - Nucleotide Metabolism	9	6
K00951	relA	GTP pyrophosphokinase [EC:2.7.6.5]	Nucleotide Metabolism	3	2
K00955	cysNC	bifunctional enzyme CysN/CysC [EC:2.7.7.4 2.7.1.25]	Metabolism of Other Amino Acids - Nucleotide Metabolism - Energy Metabolism	2	2

K00957	cysD	sulfate adenylyltransferase subunit 2 [EC:2.7.7.4]	Metabolism of Other Amino Acids - Nucleotide Metabolism - Energy Metabolism	2	2
K00962	pnp, PNPT1	polyribonucleotide nucleotidyltransferase [EC:2.7.7.8]	Folding, Sorting and Degradation - Nucleotide Metabolism	1	1
K01081	E3.1.3.5	5'-nucleotidase [EC:3.1.3.5]	Signaling Molecules and Interaction - Metabolism of Cofactors and Vitamins - Nucleotide Metabolism	3	3
K01428	ureC	urease subunit alpha [EC:3.5.1.5]	Infectious Diseases - Nucleotide Metabolism - Xenobiotics Biodegradation and Metabolism - Amino Acid Metabolism	2	1
K01429	ureB	urease subunit beta [EC:3.5.1.5]	Nucleotide Metabolism - Xenobiotics Biodegradation and Metabolism - Amino Acid Metabolism	1	1
K01486	ade	adenine deaminase [EC:3.5.4.2]	Nucleotide Metabolism	1	1
K01515	nudF	ADP-ribose pyrophosphatase [EC:3.6.1.13]	Nucleotide Metabolism	1	1
K01524	ppx-gppA	exopolyphosphatase / guanosine-5'-triphosphate,3'-diphosphate pyrophosphatase [EC:3.6.1.11 3.6.1.40]	Nucleotide Metabolism	1	1
K01588	purE	5-(carboxyamino)imidazole ribonucleotide mutase [EC:5.4.99.18]	Nucleotide Metabolism	1	1
K01756	E4.3.2.2, purB	adenylosuccinate lyase [EC:4.3.2.2]	Nucleotide Metabolism - Amino Acid Metabolism	3	3
K01768	E4.6.1.1	adenylate cyclase [EC:4.6.1.1]	Nucleotide Metabolism - Cell Growth and Death	61	5
K01923	purC	phosphoribosylaminoimidazole-succinocarboxamide synthase [EC:6.3.2.6]	Nucleotide Metabolism	1	1
K01933	purM	phosphoribosylformylglycinamidine cyclo-ligase [EC:6.3.3.1]	Nucleotide Metabolism	7	2
K01939	E6.3.4.4, purA	adenylosuccinate synthase [EC:6.3.4.4]	Nucleotide Metabolism - Amino Acid Metabolism	47	6
K01945	purD	phosphoribosylamine--glycine ligase [EC:6.3.4.13]	Nucleotide Metabolism	1	1
K01951	E6.3.5.2, guaA	GMP synthase (glutamine-hydrolysing) [EC:6.3.5.2]	Enzyme Families - Nucleotide Metabolism - Xenobiotics Biodegradation and Metabolism	1	1
K01952	E6.3.5.3, purL	phosphoribosylformylglycinamidine synthase [EC:6.3.5.3]	Nucleotide Metabolism	1	1
K02319	DPA, polB1	DNA polymerase I [EC:2.7.7.7]	Nucleotide Metabolism - Replication and Repair	468	5
K02322	DPB1	DNA polymerase II large subunit [EC:2.7.7.7]	Nucleotide Metabolism - Replication and Repair	41	2
K02323	DPB2	DNA polymerase II small subunit [EC:2.7.7.7]	Nucleotide Metabolism - Replication and Repair	5	2
K02335	DPO1, polA	DNA polymerase I [EC:2.7.7.7]	Nucleotide Metabolism - Replication and Repair	921	8
K02337	DPO3A1, dnaE	DNA polymerase III subunit alpha [EC:2.7.7.7]	Nucleotide Metabolism - Replication and Repair	178	7
K02338	DPO3B, dnaN	DNA polymerase III subunit beta [EC:2.7.7.7]	Nucleotide Metabolism - Replication and Repair	18	2
K02340	DPO3D1, holA	DNA polymerase III subunit delta [EC:2.7.7.7]	Nucleotide Metabolism - Replication and Repair	1	1
K02341	DPO3D2, holB	DNA polymerase III subunit delta' [EC:2.7.7.7]	Nucleotide Metabolism - Replication and Repair	1	1
K02342	DPO3E, dnaQ	DNA polymerase III subunit epsilon [EC:2.7.7.7]	Nucleotide Metabolism - Replication and Repair	16	3
K02343	DPO3G, dnaX	DNA polymerase III subunit gamma/tau [EC:2.7.7.7]	Nucleotide Metabolism - Replication and Repair	13	3
K03040	rpoA	DNA-directed RNA polymerase subunit alpha [EC:2.7.7.6]	Transcription - Nucleotide Metabolism - Replication and Repair	40	7
K03041	rpoA1	DNA-directed RNA polymerase subunit A' [EC:2.7.7.6]	Transcription - Nucleotide Metabolism	5	2
K03042	rpoA2	DNA-directed RNA polymerase subunit A'' [EC:2.7.7.6]	Transcription - Nucleotide Metabolism	6	1
K03043	rpoB	DNA-directed RNA polymerase subunit beta [EC:2.7.7.6]	Transcription - Nucleotide Metabolism - Replication and Repair	5	5
K03044	rpoB1	DNA-directed RNA polymerase subunit B' [EC:2.7.7.6]	Transcription - Nucleotide Metabolism	4	3
K03045	rpoB2	DNA-directed RNA polymerase subunit B'' [EC:2.7.7.6]	Transcription - Nucleotide Metabolism	2	1
K03046	rpoC	DNA-directed RNA polymerase subunit beta' [EC:2.7.7.6]	Transcription - Nucleotide Metabolism - Replication and Repair	7	4
K03053	rpoH	DNA-directed RNA polymerase subunit H [EC:2.7.7.6]	Transcription - Nucleotide Metabolism	1	1
K03056	rpoL	DNA-directed RNA polymerase subunit L [EC:2.7.7.6]	Transcription - Nucleotide Metabolism	2	1
K03060	rpoZ	DNA-directed RNA polymerase subunit omega [EC:2.7.7.6]	Transcription - Nucleotide Metabolism - Replication and Repair	1	1
K03763	DPO3A2, polC	DNA polymerase III subunit alpha, Gram-positive type [EC:2.7.7.7]	Nucleotide Metabolism - Replication and Repair	9	3
K03783	punA	purine-nucleoside phosphorylase [EC:2.4.2.1]	Metabolism of Cofactors and Vitamins - Nucleotide Metabolism	1	1
K03784	deoD	purine-nucleoside phosphorylase [EC:2.4.2.1]	Metabolism of Cofactors and Vitamins - Nucleotide Metabolism	2	2
K10807	RRM1	ribonucleoside-diphosphate reductase subunit M1 [EC:1.17.4.1]	Metabolism of Other Amino Acids - Nucleotide Metabolism - Replication and Repair	83	4
K10808	RRM2	ribonucleoside-diphosphate reductase subunit M2 [EC:1.17.4.1]	Metabolism of Other Amino Acids - Nucleotide Metabolism - Cell Growth and Death - Replication and Repair	67	5
K11175	purN	phosphoribosylglycinamide formyltransferase 1 [EC:2.1.2.2]	Metabolism of Cofactors and Vitamins - Nucleotide Metabolism	12	2
K11177	yagR	xanthine dehydrogenase YagR molybdenum-binding subunit [EC:1.17.1.4]	Nucleotide Metabolism	1	1
K13798	K13798, rpoB	DNA-directed RNA polymerase subunit B [EC:2.7.7.6]	Transcription - Nucleotide Metabolism	3	2

ko00240_Pyrimidine metabolism

K00226	pyrD	dihydroorotate oxidase [EC:1.3.3.1]	Nucleotide Metabolism	1	1
K00384	E1.8.1.9, trxB	thioredoxin reductase (NADPH) [EC:1.8.1.9]	Metabolism of Other Amino Acids - Nucleotide Metabolism	4	2
K00524	E1.17.4.1	ribonucleotide reductase, class II [EC:1.17.4.1]	Nucleotide Metabolism	3	2
K00525	E1.17.4.1A, nrdA, nrdE	ribonucleoside-diphosphate reductase alpha chain [EC:1.17.4.1]	Nucleotide Metabolism - Replication and Repair	3331	9
K00526	E1.17.4.1B, nrdB, nrdF	ribonucleoside-diphosphate reductase beta chain [EC:1.17.4.1]	Nucleotide Metabolism - Replication and Repair	869	8
K00527	nrdD	ribonucleoside-triphosphate reductase [EC:1.17.4.2]	Nucleotide Metabolism	57	7
K00560	E2.1.1.45, thyA	thymidylate synthase [EC:2.1.1.45]	Metabolism of Cofactors and Vitamins - Nucleotide Metabolism	528	9
K00762	pyrE	orotate phosphoribosyltransferase [EC:2.4.2.10]	Nucleotide Metabolism	20	2
K00857	E2.7.1.21, tdk	thymidine kinase [EC:2.7.1.21]	Nucleotide Metabolism - Xenobiotics Biodegradation and Metabolism	13	3
K00940	E2.7.4.6, ndk	nucleoside-diphosphate kinase [EC:2.7.4.6]	Nucleotide Metabolism	2	1
K00943	E2.7.4.9, tmk	dTMP kinase [EC:2.7.4.9]	Nucleotide Metabolism	64	2
K00945	cmk	cytidylate kinase [EC:2.7.4.14]	Nucleotide Metabolism	2	2
K00962	pnp, PNPT1	polyribonucleotide nucleotidyltransferase [EC:2.7.7.8]	Folding, Sorting and Degradation - Nucleotide Metabolism	1	1
K01081	E3.1.3.5	5'-nucleotidase [EC:3.1.3.5]	Signaling Molecules and Interaction - Metabolism of Cofactors and Vitamins - Nucleotide Metabolism	3	3
K01465	URA4, pyrC	dihydroorotase [EC:3.5.2.3]	Nucleotide Metabolism	1	1
K01493	comEB	dCMP deaminase [EC:3.5.4.12]	Nucleotide Metabolism - Membrane Transport	145	8
K01494	E3.5.4.13, dcd	dCTP deaminase [EC:3.5.4.13]	Nucleotide Metabolism	588	5
K01520	E3.6.1.23, dut	dUTP pyrophosphatase [EC:3.6.1.23]	Nucleotide Metabolism - Replication and Repair	181	5
K01591	pyrF	orotidine-5'-phosphate decarboxylase [EC:4.1.1.23]	Nucleotide Metabolism	3	1
K01937	E6.3.4.2, pyrG	CTP synthase [EC:6.3.4.2]	Nucleotide Metabolism	1	1
K01955	carB, CPA2	carbamoyl-phosphate synthase large subunit [EC:6.3.5.5]	Nucleotide Metabolism - Amino Acid Metabolism	2	2
K01956	carA, CPA1	carbamoyl-phosphate synthase small subunit [EC:6.3.5.5]	Nucleotide Metabolism - Amino Acid Metabolism	2	1
K02319	DPA, polB1	DNA polymerase I [EC:2.7.7.7]	Nucleotide Metabolism - Replication and Repair	468	5
K02322	DPB1	DNA polymerase II large subunit [EC:2.7.7.7]	Nucleotide Metabolism - Replication and Repair	41	2
K02323	DPB2	DNA polymerase II small subunit [EC:2.7.7.7]	Nucleotide Metabolism - Replication and Repair	5	2
K02335	DPO1, polA	DNA polymerase I [EC:2.7.7.7]	Nucleotide Metabolism - Replication and Repair	921	8
K02337	DPO3A1, dnaE	DNA polymerase III subunit alpha [EC:2.7.7.7]	Nucleotide Metabolism - Replication and Repair	178	7
K02338	DPO3B, dnaN	DNA polymerase III subunit beta [EC:2.7.7.7]	Nucleotide Metabolism - Replication and Repair	18	2
K02340	DPO3D1, holA	DNA polymerase III subunit delta [EC:2.7.7.7]	Nucleotide Metabolism - Replication and Repair	1	1
K02341	DPO3D2, holB	DNA polymerase III subunit delta' [EC:2.7.7.7]	Nucleotide Metabolism - Replication and Repair	1	1
K02342	DPO3E, dnaQ	DNA polymerase III subunit epsilon [EC:2.7.7.7]	Nucleotide Metabolism - Replication and Repair	16	3
K02343	DPO3G, dnaX	DNA polymerase III subunit gamma/tau [EC:2.7.7.7]	Nucleotide Metabolism - Replication and Repair	13	3
K02825	pyrR	pyrimidine operon attenuation protein / uracil phosphoribosyltransferase [EC:2.4.2.9]	Transcription - Nucleotide Metabolism	1	1
K03040	rpoA	DNA-directed RNA polymerase subunit alpha [EC:2.7.7.6]	Transcription - Nucleotide Metabolism - Replication and Repair	40	7
K03041	rpoA1	DNA-directed RNA polymerase subunit A' [EC:2.7.7.6]	Transcription - Nucleotide Metabolism	5	2
K03042	rpoA2	DNA-directed RNA polymerase subunit A'' [EC:2.7.7.6]	Transcription - Nucleotide Metabolism	6	1
K03043	rpoB	DNA-directed RNA polymerase subunit beta [EC:2.7.7.6]	Transcription - Nucleotide Metabolism - Replication and Repair	5	5
K03044	rpoB1	DNA-directed RNA polymerase subunit B' [EC:2.7.7.6]	Transcription - Nucleotide Metabolism	4	3
K03045	rpoB2	DNA-directed RNA polymerase subunit B'' [EC:2.7.7.6]	Transcription - Nucleotide Metabolism	2	1
K03046	rpoC	DNA-directed RNA polymerase subunit beta' [EC:2.7.7.6]	Transcription - Nucleotide Metabolism - Replication and Repair	7	4
K03053	rpoH	DNA-directed RNA polymerase subunit H [EC:2.7.7.6]	Transcription - Nucleotide Metabolism	1	1
K03056	rpoL	DNA-directed RNA polymerase subunit L [EC:2.7.7.6]	Transcription - Nucleotide Metabolism	2	1
K03060	rpoZ	DNA-directed RNA polymerase subunit omega [EC:2.7.7.6]	Transcription - Nucleotide Metabolism - Replication and Repair	1	1
K03465	E2.1.1.148, thyX, thy1	thymidylate synthase (FAD) [EC:2.1.1.148]	Metabolism of Cofactors and Vitamins - Nucleotide Metabolism	1150	7
K03763	DPO3A2, polC	DNA polymerase III subunit alpha, Gram-positive type [EC:2.7.7.7]	Nucleotide Metabolism - Replication and Repair	9	3
K03783	punA	purine-nucleoside phosphorylase [EC:2.4.2.1]	Metabolism of Cofactors and Vitamins - Nucleotide Metabolism	1	1
K03784	deoD	purine-nucleoside phosphorylase [EC:2.4.2.1]	Metabolism of Cofactors and Vitamins - Nucleotide Metabolism	2	2
K09903	pyrH	uridylate kinase [EC:2.7.4.22]	Nucleotide Metabolism	1	1
K10807	RRM1	ribonucleoside-diphosphate reductase subunit M1 [EC:1.17.4.1]	Metabolism of Other Amino Acids - Nucleotide Metabolism - Replication and Repair	83	4
K10808	RRM2	ribonucleoside-diphosphate reductase subunit M2 [EC:1.17.4.1]	Metabolism of Other Amino Acids - Nucleotide Metabolism - Cell Growth and Death - Replication and Repair	67	5
K13421	UMPS	uridine monophosphate synthetase [EC:2.4.2.10 4.1.1.23]	Nucleotide Metabolism - Xenobiotics Biodegradation and Metabolism	1	1
K13798	K13798, rpoB	DNA-directed RNA polymerase subunit B [EC:2.7.7.6]	Transcription - Nucleotide Metabolism	3	2

ko00250_Alanine, aspartate and glutamate metabolism

K00135	E1.2.1.16, gabD	succinate-semialdehyde dehydrogenase (NADP+) [EC:1.2.1.16]	Carbohydrate Metabolism - Amino Acid Metabolism	2	1
K00261	E1.4.1.3	glutamate dehydrogenase (NAD(P)+) [EC:1.4.1.3]	Metabolism of Other Amino Acids - Excretory System - Amino Acid Metabolism - Energy Metabolism	3	2
K00265	gltB	glutamate synthase (NADPH/NADH) large chain [EC:1.4.1.13 1.4.1.14]	Amino Acid Metabolism - Energy Metabolism	3	3

K00266	gltD	glutamate synthase (NADPH/NADH) small chain [EC:1.4.1.13 1.4.1.14]	Amino Acid Metabolism - Energy Metabolism	1	1
K00278	nadB	L-aspartate oxidase [EC:1.4.3.16]	Metabolism of Cofactors and Vitamins - Amino Acid Metabolism	1	1
K00294	E1.5.1.12	1-pyrroline-5-carboxylate dehydrogenase [EC:1.5.1.12]	Amino Acid Metabolism	1	1
K00764	E2.4.2.14, purF	amidophosphoribosyltransferase [EC:2.4.2.14]	Enzyme Families - Nucleotide Metabolism - Amino Acid Metabolism	2	2
K00812	E2.6.1.1A, aspB	aspartate aminotransferase [EC:2.6.1.1]	Biosynthesis of Other Secondary Metabolites - Amino Acid Metabolism - Energy Metabolism	46	3
K00820	E2.6.1.16, glmS	glucosamine--fructose-6-phosphate aminotransferase (isomerizing) [EC:2.6.1.16]	Carbohydrate Metabolism - Enzyme Families - Amino Acid Metabolism	20	4
K00823	puuE	4-aminobutyrate aminotransferase [EC:2.6.1.19]	Carbohydrate Metabolism - Metabolism of Other Amino Acids - Amino Acid Metabolism	1	1
K01424	E3.5.1.1, ansA, ansB	L-asparaginase [EC:3.5.1.1]	Metabolism of Other Amino Acids - Amino Acid Metabolism - Energy Metabolism	1	1
K01755	argH, ASL	argininosuccinate lyase [EC:4.3.2.1]	Amino Acid Metabolism	6	1
K01756	E4.3.2.2, purB	adenylosuccinate lyase [EC:4.3.2.2]	Nucleotide Metabolism - Amino Acid Metabolism	3	3
K01779	E5.1.1.13	aspartate racemase [EC:5.1.1.13]	Amino Acid Metabolism	1	1
K01915	E6.3.1.2, glnA	glutamine synthetase [EC:6.3.1.2]	Signal Transduction - Nervous System - Amino Acid Metabolism - Energy Metabolism	113	7
K01939	E6.3.4.4, purA	adenylosuccinate synthase [EC:6.3.4.4]	Nucleotide Metabolism - Amino Acid Metabolism	47	6
K01953	E6.3.5.4, asnB	asparagine synthase (glutamine-hydrolysing) [EC:6.3.5.4]	Enzyme Families - Amino Acid Metabolism - Energy Metabolism	14	2
K01955	carB, CPA2	carbamoyl-phosphate synthase large subunit [EC:6.3.5.5]	Nucleotide Metabolism - Amino Acid Metabolism	2	2
K01956	carA, CPA1	carbamoyl-phosphate synthase small subunit [EC:6.3.5.5]	Nucleotide Metabolism - Amino Acid Metabolism	2	1
K07250	gabT	4-aminobutyrate aminotransferase / (S)-3-amino-2-methylpropionate transaminase [EC:2.6.1.19 2.6.1.22]	Carbohydrate Metabolism - Metabolism of Other Amino Acids - Amino Acid Metabolism	1	1
K13821	putA	proline dehydrogenase / delta 1-pyrroline-5-carboxylate dehydrogenase [EC:1.5.99.8 1.5.1.12]	Amino Acid Metabolism	2	1

ko00253_Tetracycline biosynthesis

K01962	accA	acetyl-CoA carboxylase carboxyl transferase subunit alpha [EC:6.4.1.2]	Carbohydrate Metabolism - Metabolism of Terpenoids and Polyketides - Lipid Metabolism - Energy Metabolism	1	1
K01963	accD	acetyl-CoA carboxylase carboxyl transferase subunit beta [EC:6.4.1.2]	Carbohydrate Metabolism - Metabolism of Terpenoids and Polyketides - Lipid Metabolism - Energy Metabolism	2	2

ko00260_Glycine, serine and threonine metabolism

K00003	E1.1.1.3	homoserine dehydrogenase [EC:1.1.1.3]	Amino Acid Metabolism	1	1
K00050	E1.1.1.81, ttuD	hydroxypyruvate reductase [EC:1.1.1.81]	Carbohydrate Metabolism - Amino Acid Metabolism	1	1
K00058	serA, PHGDH	D-3-phosphoglycerate dehydrogenase [EC:1.1.1.95]	Amino Acid Metabolism - Energy Metabolism	2	1
K00130	betB, gbsA	betaine-aldehyde dehydrogenase [EC:1.2.1.8]	Amino Acid Metabolism	1	1
K00273	E1.4.3.3, DAO	D-amino-acid oxidase [EC:1.4.3.3]	Metabolism of Other Amino Acids - Biosynthesis of Other Secondary Metabolites - Transport and Catabolism - Amino Acid Metabolism	2	1
K00281	GLDC, gcvP	glycine dehydrogenase [EC:1.4.4.2]	Amino Acid Metabolism	10	3
K00282	gcvPA	glycine dehydrogenase subunit 1 [EC:1.4.4.2]	Amino Acid Metabolism	1	1
K00382	DLD, lpd, pdhD	dihydrolipoamide dehydrogenase [EC:1.8.1.4]	Carbohydrate Metabolism - Amino Acid Metabolism	1	1
K00600	E2.1.2.1, glyA	glycine hydroxymethyltransferase [EC:2.1.2.1]	Metabolism of Other Amino Acids - Metabolism of Cofactors and Vitamins - Amino Acid Metabolism - Energy Metabolism	25	4
K00605	E2.1.2.10, gcvT	aminomethyltransferase [EC:2.1.2.10]	Metabolism of Cofactors and Vitamins - Amino Acid Metabolism - Energy Metabolism	2	2
K00613	GATM	glycine amidinotransferase [EC:2.1.4.1]	Amino Acid Metabolism	5	3
K00643	E2.3.1.37, ALAS	5-aminolevulinate synthase [EC:2.3.1.37]	Metabolism of Cofactors and Vitamins - Amino Acid Metabolism	7	4
K00831	serC, PSAT1	phosphoserine aminotransferase [EC:2.6.1.52]	Metabolism of Cofactors and Vitamins - Amino Acid Metabolism - Energy Metabolism	2	1
K00836	E2.6.1.76, ectB	diaminobutyrate-2-oxoglutarate transaminase [EC:2.6.1.76]	Amino Acid Metabolism	2	1
K00928	E2.7.2.4, lysC	aspartate kinase [EC:2.7.2.4]	Amino Acid Metabolism	1	1
K00998	E2.7.8.8, pssA	phosphatidylserine synthase [EC:2.7.8.8]	Amino Acid Metabolism - Lipid Metabolism	2	2
K01079	serB, PSPH	phosphoserine phosphatase [EC:3.1.3.3]	Amino Acid Metabolism - Energy Metabolism	4	3
K01620	E4.1.2.5, ltaA	threonine aldolase [EC:4.1.2.5]	Amino Acid Metabolism	1	1
K01696	E4.2.1.20B, trpB	tryptophan synthase beta chain [EC:4.2.1.20]	Amino Acid Metabolism	1	1
K01697	E4.2.1.22, CBS	cystathionine beta-synthase [EC:4.2.1.22]	Amino Acid Metabolism	1	1
K01754	E4.3.1.19, ilvA, tdcB	threonine dehydratase [EC:4.3.1.19]	Amino Acid Metabolism	3	2
K02203	thrH	phosphoserine / homoserine phosphotransferase [EC:3.1.3.3 2.7.1.39]	Amino Acid Metabolism - Energy Metabolism	1	1
K02204	E2.7.1.39B, thrB	homoserine kinase type II [EC:2.7.1.39]	Amino Acid Metabolism	1	1
K06720	ectC	L-ectoine synthase [EC:4.2.1.108]	Amino Acid Metabolism	19	1
K13745	ddc	L-2,4-diaminobutyrate decarboxylase [EC:4.1.1.86]	Amino Acid Metabolism	1	1

ko00270_Cysteine and methionine metabolism

K00003	E1.1.1.3	homoserine dehydrogenase [EC:1.1.1.3]	Amino Acid Metabolism	1	1
K00548	E2.1.1.13, metH	5-methyltetrahydrofolate--homocysteine methyltransferase [EC:2.1.1.13]	Metabolism of Other Amino Acids - Metabolism of Cofactors and Vitamins - Amino Acid Metabolism	23	5
K00558	E2.1.1.37, DNMT, dcm	DNA (cytosine-5-)-methyltransferase [EC:2.1.1.37]	Replication and Repair - Amino Acid Metabolism	2547	9
K00640	E2.3.1.30, cysE	serine O-acetyltransferase [EC:2.3.1.30]	Amino Acid Metabolism - Energy Metabolism	2	2
K00641	E2.3.1.31, metX	homoserine O-acetyltransferase [EC:2.3.1.31]	Amino Acid Metabolism - Energy Metabolism	1	1
K00789	E2.5.1.6, metK	S-adenosylmethionine synthetase [EC:2.5.1.6]	Amino Acid Metabolism	13	4
K00797	E2.5.1.16, SRM, speE	spermidine synthase [EC:2.5.1.16]	Metabolism of Other Amino Acids - Amino Acid Metabolism	1	1
K00812	E2.6.1.1A, aspB	aspartate aminotransferase [EC:2.6.1.1]	Biosynthesis of Other Secondary Metabolites - Amino Acid Metabolism - Energy Metabolism	46	3
K00928	E2.7.2.4, lysC	aspartate kinase [EC:2.7.2.4]	Amino Acid Metabolism	1	1
K01243	mtnN, mtn, pfs	S-adenosylhomocysteine/5'-methylthioadenosine nucleosidase [EC:3.2.2.9]	Amino Acid Metabolism	1	1
K01251	E3.3.1.1, ahcY	adenosylhomocysteinase [EC:3.3.1.1]	Amino Acid Metabolism	5	2
K01611	E4.1.1.50, speD	S-adenosylmethionine decarboxylase [EC:4.1.1.50]	Amino Acid Metabolism	100	5
K01697	E4.2.1.22, CBS	cystathionine beta-synthase [EC:4.2.1.22]	Amino Acid Metabolism	1	1
K01738	cysK	cysteine synthase A [EC:2.5.1.47]	Amino Acid Metabolism - Energy Metabolism	30	5
K01740	E2.5.1.49, metY	O-acetylhomoserine (thiol)-lyase [EC:2.5.1.49]	Amino Acid Metabolism	4	2
K01760	metC	cystathionine beta-lyase [EC:4.4.1.8]	Metabolism of Other Amino Acids - Amino Acid Metabolism - Energy Metabolism	2	1
K07173	luxS	S-ribosylhomocysteine lyase [EC:4.4.1.21]	Infectious Diseases - Amino Acid Metabolism	1	1
K08965	mtnW	2,3-diketo-5-methylthiopentyl-1-phosphate enolase [EC:3.1.3.77]	Amino Acid Metabolism	1	1
K10764	metZ	O-succinylhomoserine sulfhydrylase [EC:2.5.1.-]	Amino Acid Metabolism	1	1
K12339	cysM	cysteine synthase B [EC:2.5.1.47]	Amino Acid Metabolism - Energy Metabolism	5	2

ko00280_Valine, leucine and isoleucine degradation

K00128	E1.2.1.3	aldehyde dehydrogenase (NAD+) [EC:1.2.1.3]	Carbohydrate Metabolism - Metabolism of Other Amino Acids - Xenobiotics Biodegradation and Metabolism - Metabolism of Terpenoids and Polyketides - Amino Acid Metabolism - Lipid Metabolism	4	4
K00140	E1.2.1.27, mmsA, iolA	methylmalonate-semialdehyde dehydrogenase [EC:1.2.1.27]	Carbohydrate Metabolism - Amino Acid Metabolism	1	1
K00167	E1.2.4.4B, bkdA2	2-oxoisovalerate dehydrogenase E1 component, beta subunit [EC:1.2.4.4]	Amino Acid Metabolism	1	1
K00249	E1.3.99.3, ACADM, acd	acyl-CoA dehydrogenase [EC:1.3.99.3]	Carbohydrate Metabolism - Metabolism of Other Amino Acids - Endocrine System - Amino Acid Metabolism - Lipid Metabolism	1	1
K00382	DLD, lpd, pdhD	dihydrolipoamide dehydrogenase [EC:1.8.1.4]	Carbohydrate Metabolism - Amino Acid Metabolism	1	1
K00626	E2.3.1.9, atoB	acetyl-CoA C-acetyltransferase [EC:2.3.1.9]	Carbohydrate Metabolism - Signal Transduction - Xenobiotics Biodegradation and Metabolism - Metabolism of Terpenoids and Polyketides - Amino Acid Metabolism - Lipid Metabolism - Energy Metabolism	4	3
K00826	E2.6.1.42, ilvE	branched-chain amino acid aminotransferase [EC:2.6.1.42]	Metabolism of Cofactors and Vitamins - Amino Acid Metabolism	3	2
K01640	E4.1.3.4, HMGCL, hmgL	hydroxymethylglutaryl-CoA lyase [EC:4.1.3.4]	Carbohydrate Metabolism - Transport and Catabolism - Metabolism of Terpenoids and Polyketides - Amino Acid Metabolism - Lipid Metabolism	18	1
K01692	E4.2.1.17, paaG	enoyl-CoA hydratase [EC:4.2.1.17]	Carbohydrate Metabolism - Metabolism of Other Amino Acids - Xenobiotics Biodegradation and Metabolism - Metabolism of Terpenoids and Polyketides - Amino Acid Metabolism - Lipid Metabolism	33	3
K01825	fadB	3-hydroxyacyl-CoA dehydrogenase / enoyl-CoA hydratase / 3-hydroxybutyryl-CoA epimerase / enoyl-CoA isomerase [EC:1.1.1.35 4.2.1.17 5.1.2.3 5.3.3.8]	Carbohydrate Metabolism - Metabolism of Other Amino Acids - Xenobiotics Biodegradation and Metabolism - Metabolism of Terpenoids and Polyketides - Amino Acid Metabolism - Lipid Metabolism - Energy Metabolism	1	1
K01847	MUT	methylmalonyl-CoA mutase [EC:5.4.99.2]	Carbohydrate Metabolism - Amino Acid Metabolism - Energy Metabolism	2	2
K01848	E5.4.99.2A, mcmA1	methylmalonyl-CoA mutase, N-terminal domain [EC:5.4.99.2]	Carbohydrate Metabolism - Amino Acid Metabolism - Energy Metabolism	1	1
K01969	E6.4.1.4B	3-methylcrotonyl-CoA carboxylase beta subunit [EC:6.4.1.4]	Amino Acid Metabolism	1	1
K07250	gabT	4-aminobutyrate aminotransferase / (S)-3-amino-2-methylpropionate transaminase [EC:2.6.1.19 2.6.1.22]	Carbohydrate Metabolism - Metabolism of Other Amino Acids - Amino Acid Metabolism	1	1
K11381	E1.2.4.4C, bkdA	2-oxoisovalerate dehydrogenase E1 component [EC:1.2.4.4]	Amino Acid Metabolism	6	2

ko00281_Geraniol degradation

K00257	E1.3.99.-		Xenobiotics Biodegradation and Metabolism - Metabolism of Terpenoids and Polyketides	2	2
K01640	E4.1.3.4, HMGCL, hmgL	hydroxymethylglutaryl-CoA lyase [EC:4.1.3.4]	Carbohydrate Metabolism - Transport and Catabolism - Metabolism of Terpenoids and Polyketides - Amino Acid Metabolism - Lipid Metabolism	18	1

K01692	E4.2.1.17, paaG	enoyl-CoA hydratase [EC:4.2.1.17]	Carbohydrate Metabolism - Metabolism of Other Amino Acids - Xenobiotics Biodegradation and Metabolism - Metabolism of Terpenoids and Polyketides - Amino Acid Metabolism - Lipid Metabolism	33	3
K01825	fadB	3-hydroxyacyl-CoA dehydrogenase / enoyl-CoA hydratase / 3-hydroxybutyryl-CoA epimerase / enoyl-CoA isomerase [EC:1.1.1.35 4.2.1.17 5.1.2.3 5.3.3.8]	Carbohydrate Metabolism - Metabolism of Other Amino Acids - Xenobiotics Biodegradation and Metabolism - Metabolism of Terpenoids and Polyketides - Amino Acid Metabolism - Lipid Metabolism - Energy Metabolism	1	1
ko00290_Valine, leucine and isoleucine biosynthesis					
K00053	ilvC	ketol-acid reductoisomerase [EC:1.1.1.86]	Metabolism of Cofactors and Vitamins - Amino Acid Metabolism	2	2
K00161	PDHA, pdhA	pyruvate dehydrogenase E1 component subunit alpha [EC:1.2.4.1]	Carbohydrate Metabolism - Amino Acid Metabolism	57	3
K00162	PDHB, pdhB	pyruvate dehydrogenase E1 component subunit beta [EC:1.2.4.1]	Carbohydrate Metabolism - Amino Acid Metabolism	37	3
K00826	E2.6.1.42, ilvE	branched-chain amino acid aminotransferase [EC:2.6.1.42]	Metabolism of Cofactors and Vitamins - Amino Acid Metabolism	3	2
K01649	E2.3.3.13, leuA	2-isopropylmalate synthase [EC:2.3.3.13]	Carbohydrate Metabolism - Amino Acid Metabolism	1	1
K01652	E2.2.1.6L, ilvB, ilvG, ilvI	acetolactate synthase I/II/III large subunit [EC:2.2.1.6]	Carbohydrate Metabolism - Metabolism of Cofactors and Vitamins - Amino Acid Metabolism	300	5
K01703	leuC	3-isopropylmalate/(R)-2-methylmalate dehydratase large subunit [EC:4.2.1.33 4.2.1.35]	Carbohydrate Metabolism - Amino Acid Metabolism	2	2
K01704	leuD	3-isopropylmalate/(R)-2-methylmalate dehydratase small subunit [EC:4.2.1.33 4.2.1.35]	Carbohydrate Metabolism - Amino Acid Metabolism	1	1
K01754	E4.3.1.19, ilvA, tdcB	threonine dehydratase [EC:4.3.1.19]	Amino Acid Metabolism	3	2
K01869	LARS, leuS	leucyl-tRNA synthetase [EC:6.1.1.4]	Translation - Amino Acid Metabolism	2	2
K01870	IARS, ileS	isoleucyl-tRNA synthetase [EC:6.1.1.5]	Translation - Amino Acid Metabolism	3	2
K01873	VARS, valS	valyl-tRNA synthetase [EC:6.1.1.9]	Translation - Amino Acid Metabolism	3	2
ko00300_Lysine biosynthesis					
K00003	E1.1.1.3	homoserine dehydrogenase [EC:1.1.1.3]	Amino Acid Metabolism	1	1
K00145	argC	N-acetyl-gamma-glutamyl-phosphate/N-acetyl-gamma-aminoadipyl-phosphate reductase [EC:1.2.1.38 1.2.1.-]	Amino Acid Metabolism	1	1
K00215	dapB	dihydrodipicolinate reductase [EC:1.3.1.26]	Amino Acid Metabolism	1	1
K00674	dapD	2,3,4,5-tetrahydropyridine-2-carboxylate N-succinyltransferase [EC:2.3.1.117]	Amino Acid Metabolism	4	2
K00928	E2.7.2.4, lysC	aspartate kinase [EC:2.7.2.4]	Amino Acid Metabolism	1	1
K01439	dapE	succinyl-diaminopimelate desuccinylase [EC:3.5.1.18]	Amino Acid Metabolism	1	1
K01586	lysA	diaminopimelate decarboxylase [EC:4.1.1.20]	Amino Acid Metabolism	3	2
K01714	dapA	dihydrodipicolinate synthase [EC:4.2.1.52]	Amino Acid Metabolism	1	1
K01778	dapF	diaminopimelate epimerase [EC:5.1.1.7]	Amino Acid Metabolism	1	1
K01929	murF	UDP-N-acetylmuramoylalanyl-D-glutamyl-2,6-diaminopimelate--D-alanyl-D-alanine ligase [EC:6.3.2.10]	Glycan Biosynthesis and Metabolism - Amino Acid Metabolism	1	1
K03918	lat	L-lysine 6-transaminase [EC:2.6.1.36]	Amino Acid Metabolism	2	2
ko00310_Lysine degradation					
K00128	E1.2.1.3	aldehyde dehydrogenase (NAD+) [EC:1.2.1.3]	Carbohydrate Metabolism - Metabolism of Other Amino Acids - Xenobiotics Biodegradation and Metabolism - Metabolism of Terpenoids and Polyketides - Amino Acid Metabolism - Lipid Metabolism	4	4
K00164	OGDH, sucA	2-oxoglutarate dehydrogenase E1 component [EC:1.2.4.2]	Carbohydrate Metabolism - Amino Acid Metabolism	2	1
K00626	E2.3.1.9, atoB	acetyl-CoA C-acetyltransferase [EC:2.3.1.9]	Carbohydrate Metabolism - Signal Transduction - Xenobiotics Biodegradation and Metabolism - Metabolism of Terpenoids and Polyketides - Amino Acid Metabolism - Lipid Metabolism - Energy Metabolism	4	3
K00658	DLST, sucB	2-oxoglutarate dehydrogenase E2 component (dihydrolipoamide succinyltransferase) [EC:2.3.1.61]	Carbohydrate Metabolism - Amino Acid Metabolism	2	2
K01423	E3.4.-.-		Metabolism of Cofactors and Vitamins - Amino Acid Metabolism	7	4
K01692	E4.2.1.17, paaG	enoyl-CoA hydratase [EC:4.2.1.17]	Carbohydrate Metabolism - Metabolism of Other Amino Acids - Xenobiotics Biodegradation and Metabolism - Metabolism of Terpenoids and Polyketides - Amino Acid Metabolism - Lipid Metabolism	33	3
K01825	fadB	3-hydroxyacyl-CoA dehydrogenase / enoyl-CoA hydratase / 3-hydroxybutyryl-CoA epimerase / enoyl-CoA isomerase [EC:1.1.1.35 4.2.1.17 5.1.2.3 5.3.3.8]	Carbohydrate Metabolism - Metabolism of Other Amino Acids - Xenobiotics Biodegradation and Metabolism - Metabolism of Terpenoids and Polyketides - Amino Acid Metabolism - Lipid Metabolism - Energy Metabolism	1	1
K01843	E5.4.3.2, kamA	lysine 2,3-aminomutase [EC:5.4.3.2]	Amino Acid Metabolism	2	1
ko00311_Penicillin and cephalosporin biosynthesis					
K00273	E1.4.3.3, DAO	D-amino-acid oxidase [EC:1.4.3.3]	Metabolism of Other Amino Acids - Biosynthesis of Other Secondary Metabolites - Transport and Catabolism - Amino Acid Metabolism	2	1
K01434	E3.5.1.11	penicillin amidase [EC:3.5.1.11]	Enzyme Families - Biosynthesis of Other Secondary Metabolites	1	1
K01467	E3.5.2.6, ampC, penP	beta-lactamase [EC:3.5.2.6]	Signal Transduction - Biosynthesis of Other Secondary Metabolites	1	1
ko00312_beta-Lactam resistance					
K01467	E3.5.2.6, ampC, penP	beta-lactamase [EC:3.5.2.6]	Signal Transduction - Biosynthesis of Other Secondary Metabolites	1	1
ko00330_Arginine and proline metabolism					
K00128	E1.2.1.3	aldehyde dehydrogenase (NAD+) [EC:1.2.1.3]	Carbohydrate Metabolism - Metabolism of Other Amino Acids - Xenobiotics Biodegradation and Metabolism - Metabolism of Terpenoids and Polyketides - Amino Acid Metabolism - Lipid Metabolism	4	4
K00145	argC	N-acetyl-gamma-glutamyl-phosphate/N-acetyl-gamma-aminoadipyl-phosphate reductase [EC:1.2.1.38 1.2.1.-]	Amino Acid Metabolism	1	1
K00147	proA	glutamate-5-semialdehyde dehydrogenase [EC:1.2.1.41]	Amino Acid Metabolism	1	1
K00261	E1.4.1.3	glutamate dehydrogenase (NAD(P)+) [EC:1.4.1.3]	Metabolism of Other Amino Acids - Excretory System - Amino Acid Metabolism - Energy Metabolism	3	2
K00273	E1.4.3.3, DAO	D-amino-acid oxidase [EC:1.4.3.3]	Metabolism of Other Amino Acids - Biosynthesis of Other Secondary Metabolites - Transport and Catabolism - Amino Acid Metabolism	2	1
K00294	E1.5.1.12	1-pyrroline-5-carboxylate dehydrogenase [EC:1.5.1.12]	Amino Acid Metabolism	1	1
K00472	E1.14.11.2	prolyl 4-hydroxylase [EC:1.14.11.2]	Amino Acid Metabolism	25	4
K00613	GATM	glycine amidinotransferase [EC:2.1.4.1]	Amino Acid Metabolism	5	3
K00797	E2.5.1.16, SRM, speE	spermidine synthase [EC:2.5.1.16]	Metabolism of Other Amino Acids - Amino Acid Metabolism	1	1
K00812	E2.6.1.1A, aspB	aspartate aminotransferase [EC:2.6.1.1]	Biosynthesis of Other Secondary Metabolites - Amino Acid Metabolism - Energy Metabolism	46	3
K00931	proB	glutamate 5-kinase [EC:2.7.2.11]	Amino Acid Metabolism	2	2
K01426	E3.5.1.4, amiE	amidase [EC:3.5.1.4]	Metabolism of Other Amino Acids - Xenobiotics Biodegradation and Metabolism - Amino Acid Metabolism	3	1
K01428	ureC	urease subunit alpha [EC:3.5.1.5]	Infectious Diseases - Nucleotide Metabolism - Xenobiotics Biodegradation and Metabolism - Amino Acid Metabolism	2	1
K01429	ureB	urease subunit beta [EC:3.5.1.5]	Nucleotide Metabolism - Xenobiotics Biodegradation and Metabolism - Amino Acid Metabolism	1	1
K01438	E3.5.1.16, argE	acetylornithine deacetylase [EC:3.5.1.16]	Amino Acid Metabolism	1	1
K01476	E3.5.3.1, rocF, arg	arginase [EC:3.5.3.1]	Infectious Diseases - Amino Acid Metabolism	11	1
K01480	E3.5.3.11, speB	agmatinase [EC:3.5.3.11]	Amino Acid Metabolism	2	1
K01581	E4.1.1.17, ODC1, speC, speF	ornithine decarboxylase [EC:4.1.1.17]	Metabolism of Other Amino Acids - Amino Acid Metabolism	1	1
K01611	E4.1.1.50, speD	S-adenosylmethionine decarboxylase [EC:4.1.1.50]	Amino Acid Metabolism	100	5
K01750	E4.3.1.12, ocd	ornithine cyclodeaminase [EC:4.3.1.12]	Amino Acid Metabolism	1	1
K01755	argH, ASL	argininosuccinate lyase [EC:4.3.2.1]	Amino Acid Metabolism	6	1
K01915	E6.3.1.2, glnA	glutamine synthetase [EC:6.3.1.2]	Signal Transduction - Nervous System - Amino Acid Metabolism - Energy Metabolism	113	7
K09251	E2.6.1.82	putrescine aminotransferase [EC:2.6.1.82]	Amino Acid Metabolism	2	1
K09472	puuC, aldH	gamma-glutamyl-gamma-aminobutyraldehyde dehydrogenase [EC:1.2.1.-]	Amino Acid Metabolism	1	1
K10536	E3.5.3.12	agmatine deiminase [EC:3.5.3.12]	Amino Acid Metabolism	2	1
K13747	nspC	carboxynorspermidine decarboxylase [EC:4.1.1.-]	Amino Acid Metabolism	1	1
K13821	putA	proline dehydrogenase / delta 1-pyrroline-5-carboxylate dehydrogenase [EC:1.5.99.8 1.5.1.12]	Amino Acid Metabolism	2	1
ko00340_Histidine metabolism					
K00013	hisD	histidinol dehydrogenase [EC:1.1.1.23]	Amino Acid Metabolism	1	1
K00128	E1.2.1.3	aldehyde dehydrogenase (NAD+) [EC:1.2.1.3]	Carbohydrate Metabolism - Metabolism of Other Amino Acids - Xenobiotics Biodegradation and Metabolism - Metabolism of Terpenoids and Polyketides - Amino Acid Metabolism - Lipid Metabolism	4	4
K00599	E2.1.1.-		Xenobiotics Biodegradation and Metabolism - Amino Acid Metabolism	55	3
K00817	hisC	histidinol-phosphate aminotransferase [EC:2.6.1.9]	Biosynthesis of Other Secondary Metabolites - Amino Acid Metabolism	6	3
K01468	E3.5.2.7, hutI	imidazolonepropionase [EC:3.5.2.7]	Amino Acid Metabolism	1	1
K01693	E4.2.1.19, hisB	imidazoleglycerol-phosphate dehydratase [EC:4.2.1.19]	Amino Acid Metabolism	1	1

K01814	E5.3.1.16, hisA	phosphoribosylformimino-5-aminoimidazole carboxamide ribotide isomerase [EC:5.3.1.16]	Amino Acid Metabolism	1	1
K02500	hisF	cyclase [EC:4.1.3.-]	Amino Acid Metabolism	2	1
K02501	hisH	glutamine amidotransferase [EC:2.4.2.-]	Amino Acid Metabolism	1	1

ko00350_Tyrosine metabolism

K00001	E1.1.1.1, adh	alcohol dehydrogenase [EC:1.1.1.1]	Carbohydrate Metabolism - Metabolism of Cofactors and Vitamins - Xenobiotics Biodegradation and Metabolism - Amino Acid Metabolism - Lipid Metabolism	1	1
K00121	frmA, ADH5, adhC	S-(hydroxymethyl)glutathione dehydrogenase / alcohol dehydrogenase [EC:1.1.1.284 1.1.1.1]	Carbohydrate Metabolism - Metabolism of Cofactors and Vitamins - Xenobiotics Biodegradation and Metabolism - Amino Acid Metabolism - Lipid Metabolism - Energy Metabolism	8	3
K00135	E1.2.1.16, gabD	succinate-semialdehyde dehydrogenase (NADP+) [EC:1.2.1.16]	Carbohydrate Metabolism - Amino Acid Metabolism	2	1
K00450	E1.13.11.4	gentisate 1,2-dioxygenase [EC:1.13.11.4]	Amino Acid Metabolism	1	1
K00599	E2.1.1.-		Xenobiotics Biodegradation and Metabolism - Amino Acid Metabolism	55	3
K00680	E2.3.1.-		Xenobiotics Biodegradation and Metabolism - Metabolism of Terpenoids and Polyketides - Amino Acid Metabolism	9	1
K00812	E2.6.1.1A, aspB	aspartate aminotransferase [EC:2.6.1.1]	Biosynthesis of Other Secondary Metabolites - Amino Acid Metabolism - Energy Metabolism	46	3
K00817	hisC	histidinol-phosphate aminotransferase [EC:2.6.1.9]	Biosynthesis of Other Secondary Metabolites - Amino Acid Metabolism	6	3
K02510	hpaI	2,4-dihydroxyhept-2-ene-1,7-dioic acid aldolase [EC:4.1.2.-]	Amino Acid Metabolism	2	2
K13953	adhP	alcohol dehydrogenase, propanol-preferring [EC:1.1.1.1]	Carbohydrate Metabolism - Metabolism of Cofactors and Vitamins - Xenobiotics Biodegradation and Metabolism - Amino Acid Metabolism - Lipid Metabolism	1	1

ko00360_Phenylalanine metabolism

K00285	dadA	D-amino-acid dehydrogenase [EC:1.4.99.1]	Amino Acid Metabolism - Energy Metabolism	19	4
K00529	hcaD	ferredoxin--NAD+ reductase [EC:1.18.1.3]	Xenobiotics Biodegradation and Metabolism - Amino Acid Metabolism - Lipid Metabolism	1	1
K00588	E2.1.1.104	caffeoyl-CoA O-methyltransferase [EC:2.1.1.104]	Biosynthesis of Other Secondary Metabolites - Amino Acid Metabolism	1	1
K00812	E2.6.1.1A, aspB	aspartate aminotransferase [EC:2.6.1.1]	Biosynthesis of Other Secondary Metabolites - Amino Acid Metabolism - Energy Metabolism	46	3
K00817	hisC	histidinol-phosphate aminotransferase [EC:2.6.1.9]	Biosynthesis of Other Secondary Metabolites - Amino Acid Metabolism	6	3
K01426	E3.5.1.4, amiE	amidase [EC:3.5.1.4]	Metabolism of Other Amino Acids - Xenobiotics Biodegradation and Metabolism - Amino Acid Metabolism	3	1
K01666	E4.1.3.39, mhpE	4-hydroxy 2-oxovalerate aldolase [EC:4.1.3.39]	Xenobiotics Biodegradation and Metabolism - Amino Acid Metabolism	35	3
K01912	E6.2.1.30, paaK	phenylacetate-CoA ligase [EC:6.2.1.30]	Amino Acid Metabolism	2	2
K02554	mhpD	2-keto-4-pentenoate hydratase [EC:4.2.1.80]	Xenobiotics Biodegradation and Metabolism - Amino Acid Metabolism	1	1
K03782	katG	catalase/peroxidase [EC:1.11.1.6 1.11.1.7]	Biosynthesis of Other Secondary Metabolites - Amino Acid Metabolism - Energy Metabolism	9	1
K05709	hcaA2, hcaF	small terminal subunit of phenylpropionate dioxygenase [EC:1.14.12.19]	Amino Acid Metabolism	1	1

ko00361_Chlorocyclohexane and chlorobenzene degradation

K01563	dhaA	haloalkane dehalogenase [EC:3.8.1.5]	Xenobiotics Biodegradation and Metabolism	1	1

ko00362_Benzoate degradation

K00626	E2.3.1.9, atoB	acetyl-CoA C-acetyltransferase [EC:2.3.1.9]	Carbohydrate Metabolism - Signal Transduction - Xenobiotics Biodegradation and Metabolism - Metabolism of Terpenoids and Polyketides - Amino Acid Metabolism - Lipid Metabolism - Energy Metabolism	4	3
K00680	E2.3.1.-		Xenobiotics Biodegradation and Metabolism - Metabolism of Terpenoids and Polyketides - Amino Acid Metabolism	9	1
K01666	E4.1.3.39, mhpE	4-hydroxy 2-oxovalerate aldolase [EC:4.1.3.39]	Xenobiotics Biodegradation and Metabolism - Amino Acid Metabolism	35	3
K01692	E4.2.1.17, paaG	enoyl-CoA hydratase [EC:4.2.1.17]	Carbohydrate Metabolism - Metabolism of Other Amino Acids - Xenobiotics Biodegradation and Metabolism - Metabolism of Terpenoids and Polyketides - Amino Acid Metabolism - Lipid Metabolism	33	3
K01726	E4.2.1.-		Carbohydrate Metabolism - Xenobiotics Biodegradation and Metabolism - Metabolism of Terpenoids and Polyketides	1	1
K02554	mhpD	2-keto-4-pentenoate hydratase [EC:4.2.1.80]	Xenobiotics Biodegradation and Metabolism - Amino Acid Metabolism	1	1
K04073	mhpF	acetaldehyde dehydrogenase [EC:1.2.1.10]	Carbohydrate Metabolism - Xenobiotics Biodegradation and Metabolism	43	2
K05783	benD	1,6-dihydroxycyclohexa-2,4-diene-1-carboxylate dehydrogenase [EC:1.3.1.25]	Xenobiotics Biodegradation and Metabolism	1	1

ko00363_Bisphenol degradation

K00100	E1.1.1.-		Carbohydrate Metabolism - Xenobiotics Biodegradation and Metabolism - Lipid Metabolism	1	1
K01726	E4.2.1.-		Carbohydrate Metabolism - Xenobiotics Biodegradation and Metabolism - Metabolism of Terpenoids and Polyketides	1	1

ko00364_Fluorobenzoate degradation

K05783	benD	1,6-dihydroxycyclohexa-2,4-diene-1-carboxylate dehydrogenase [EC:1.3.1.25]	Xenobiotics Biodegradation and Metabolism	1	1

ko00380_Tryptophan metabolism

K00128	E1.2.1.3	aldehyde dehydrogenase (NAD+) [EC:1.2.1.3]	Carbohydrate Metabolism - Metabolism of Other Amino Acids - Xenobiotics Biodegradation and Metabolism - Metabolism of Terpenoids and Polyketides - Amino Acid Metabolism - Lipid Metabolism	4	4
K00164	OGDH, sucA	2-oxoglutarate dehydrogenase E1 component [EC:1.2.4.2]	Carbohydrate Metabolism - Amino Acid Metabolism	2	1
K00626	E2.3.1.9, atoB	acetyl-CoA C-acetyltransferase [EC:2.3.1.9]	Carbohydrate Metabolism - Signal Transduction - Xenobiotics Biodegradation and Metabolism - Metabolism of Terpenoids and Polyketides - Amino Acid Metabolism - Lipid Metabolism - Energy Metabolism	4	3
K01426	E3.5.1.4, amiE	amidase [EC:3.5.1.4]	Metabolism of Other Amino Acids - Xenobiotics Biodegradation and Metabolism - Amino Acid Metabolism	3	1
K01501	E3.5.5.1	nitrilase [EC:3.5.5.1]	Metabolism of Other Amino Acids - Xenobiotics Biodegradation and Metabolism - Amino Acid Metabolism - Energy Metabolism	1	1
K01556	E3.7.1.3	kynureninase [EC:3.7.1.3]	Amino Acid Metabolism	1	1
K01667	E4.1.99.1, tnaA	tryptophanase [EC:4.1.99.1]	Amino Acid Metabolism - Energy Metabolism	1	1
K01692	E4.2.1.17, paaG	enoyl-CoA hydratase [EC:4.2.1.17]	Carbohydrate Metabolism - Metabolism of Other Amino Acids - Xenobiotics Biodegradation and Metabolism - Metabolism of Terpenoids and Polyketides - Amino Acid Metabolism - Lipid Metabolism	33	3
K01825	fadB	3-hydroxyacyl-CoA dehydrogenase / enoyl-CoA hydratase / 3-hydroxybutyryl-CoA epimerase / enoyl-CoA isomerase [EC:1.1.1.35 4.2.1.17 5.1.2.3 5.3.3.8]	Carbohydrate Metabolism - Metabolism of Other Amino Acids - Xenobiotics Biodegradation and Metabolism - Metabolism of Terpenoids and Polyketides - Amino Acid Metabolism - Lipid Metabolism - Energy Metabolism	1	1
K01867	WARS, trpS	tryptophanyl-tRNA synthetase [EC:6.1.1.2]	Translation - Amino Acid Metabolism	3	1
K03781	katE, CAT	catalase [EC:1.11.1.6]	Neurodegenerative Diseases - Transport and Catabolism - Amino Acid Metabolism - Energy Metabolism	1	1
K03782	katG	catalase/peroxidase [EC:1.11.1.6 1.11.1.7]	Biosynthesis of Other Secondary Metabolites - Amino Acid Metabolism - Energy Metabolism	9	1
K04103	E4.1.1.74, ipdC	indolepyruvate decarboxylase [EC:4.1.1.74]	Amino Acid Metabolism	1	1

ko00400_Phenylalanine, tyrosine and tryptophan biosynthesis

K00014	aroE	shikimate dehydrogenase [EC:1.1.1.25]	Amino Acid Metabolism	3	1
K00210	E1.3.1.12	prephenate dehydrogenase [EC:1.3.1.12]	Biosynthesis of Other Secondary Metabolites - Amino Acid Metabolism	1	1
K00812	E2.6.1.1A, aspB	aspartate aminotransferase [EC:2.6.1.1]	Biosynthesis of Other Secondary Metabolites - Amino Acid Metabolism - Energy Metabolism	46	3
K00817	hisC	histidinol-phosphate aminotransferase [EC:2.6.1.9]	Biosynthesis of Other Secondary Metabolites - Amino Acid Metabolism	6	3
K01657	trpE	anthranilate synthase component I [EC:4.1.3.27]	Amino Acid Metabolism	2	2
K01696	E4.2.1.20B, trpB	tryptophan synthase beta chain [EC:4.2.1.20]	Amino Acid Metabolism	1	1
K01735	aroB	3-dehydroquinate synthase [EC:4.2.3.4]	Amino Acid Metabolism	28	4
K03856	AROA2, aroA	3-deoxy-7-phosphoheptulonate synthase [EC:2.5.1.54]	Amino Acid Metabolism	3	3
K04516	AROA1, aroA	chorismate mutase [EC:5.4.99.5]	Amino Acid Metabolism	1	1
K04518	pheA2	prephenate dehydratase [EC:4.2.1.51]	Amino Acid Metabolism	1	1
K13853	aroG, aroA	3-deoxy-7-phosphoheptulonate synthase / chorismate mutase [EC:2.5.1.54 5.4.99.5]	Amino Acid Metabolism	1	1
K14170	pheA	chorismate mutase / prephenate dehydratase [EC:5.4.99.5 4.2.1.51]	Amino Acid Metabolism	1	1

ko00401_Novobiocin biosynthesis

K00210	E1.3.1.12	prephenate dehydrogenase [EC:1.3.1.12]	Biosynthesis of Other Secondary Metabolites - Amino Acid Metabolism	1	1
K00812	E2.6.1.1A, aspB	aspartate aminotransferase [EC:2.6.1.1]	Biosynthesis of Other Secondary Metabolites - Amino Acid Metabolism - Energy Metabolism	46	3
K00817	hisC	histidinol-phosphate aminotransferase [EC:2.6.1.9]	Biosynthesis of Other Secondary Metabolites - Amino Acid Metabolism	6	3

ko00410_beta-Alanine metabolism

K00128	E1.2.1.3	aldehyde dehydrogenase (NAD+) [EC:1.2.1.3]	Carbohydrate Metabolism - Metabolism of Other Amino Acids - Xenobiotics Biodegradation and Metabolism - Metabolism of Terpenoids and Polyketides - Amino Acid Metabolism - Lipid Metabolism	4	4

K00249	E1.3.99.3, ACADM, acd	acyl-CoA dehydrogenase [EC:1.3.99.3]	Carbohydrate Metabolism - Metabolism of Other Amino Acids - Endocrine System - Amino Acid Metabolism - Lipid Metabolism	1	1
K00797	E2.5.1.16, SRM, speE	spermidine synthase [EC:2.5.1.16]	Metabolism of Other Amino Acids - Amino Acid Metabolism	1	1
K00823	puuE	4-aminobutyrate aminotransferase [EC:2.6.1.19]	Carbohydrate Metabolism - Metabolism of Other Amino Acids - Amino Acid Metabolism	1	1
K01692	E4.2.1.17, paaG	enoyl-CoA hydratase [EC:4.2.1.17]	Carbohydrate Metabolism - Metabolism of Other Amino Acids - Xenobiotics Biodegradation and Metabolism - Metabolism of Terpenoids and Polyketides - Amino Acid Metabolism - Lipid Metabolism	33	3
K01825	fadB	3-hydroxyacyl-CoA dehydrogenase / enoyl-CoA hydratase / 3-hydroxybutyryl-CoA epimerase / enoyl-CoA isomerase [EC:1.1.1.35 4.2.1.17 5.1.2.3 5.3.3.8]	Carbohydrate Metabolism - Metabolism of Other Amino Acids - Xenobiotics Biodegradation and Metabolism - Metabolism of Terpenoids and Polyketides - Amino Acid Metabolism - Lipid Metabolism - Energy Metabolism	1	1
K07250	gabT	4-aminobutyrate aminotransferase / (S)-3-amino-2-methylpropionate transaminase [EC:2.6.1.19 2.6.1.22]	Carbohydrate Metabolism - Metabolism of Other Amino Acids - Amino Acid Metabolism	1	1
ko00440_Phosphonate and phosphinate metabolism					
K00968	E2.7.7.15, PCYT1	choline-phosphate cytidylyltransferase [EC:2.7.7.15]	Metabolism of Other Amino Acids - Lipid Metabolism	12	3
K01841	E5.4.2.9	phosphoenolpyruvate phosphomutase [EC:5.4.2.9]	Metabolism of Other Amino Acids	2	1
ko00450_Selenocompound metabolism					
K00384	E1.8.1.9, trxB	thioredoxin reductase (NADPH) [EC:1.8.1.9]	Metabolism of Other Amino Acids - Nucleotide Metabolism	4	2
K00548	E2.1.1.13, metH	5-methyltetrahydrofolate--homocysteine methyltransferase [EC:2.1.1.13]	Metabolism of Other Amino Acids - Metabolism of Cofactors and Vitamins - Amino Acid Metabolism	23	5
K00955	cysNC	bifunctional enzyme CysN/CysC [EC:2.7.7.4 2.7.1.25]	Metabolism of Other Amino Acids - Nucleotide Metabolism - Energy Metabolism	2	2
K00957	cysD	sulfate adenylyltransferase subunit 2 [EC:2.7.7.4]	Metabolism of Other Amino Acids - Nucleotide Metabolism - Energy Metabolism	2	2
K01760	metC	cystathionine beta-lyase [EC:4.4.1.8]	Metabolism of Other Amino Acids - Amino Acid Metabolism - Energy Metabolism	2	1
K01874	MARS, metG	methionyl-tRNA synthetase [EC:6.1.1.10]	Translation - Metabolism of Other Amino Acids - Amino Acid Metabolism	6	3
K11717	sufS	cysteine desulfurase / selenocysteine lyase [EC:2.8.1.7 4.4.1.16]	Metabolism of Other Amino Acids - Metabolism of Cofactors and Vitamins	1	1
ko00460_Cyanoamino acid metabolism					
K00600	E2.1.2.1, glyA	glycine hydroxymethyltransferase [EC:2.1.2.1]	Metabolism of Other Amino Acids - Metabolism of Cofactors and Vitamins - Amino Acid Metabolism - Energy Metabolism	25	4
K01424	E3.5.1.1, ansA, ansB	L-asparaginase [EC:3.5.1.1]	Metabolism of Other Amino Acids - Amino Acid Metabolism - Energy Metabolism	1	1
K01426	E3.5.1.4, amiE	amidase [EC:3.5.1.4]	Metabolism of Other Amino Acids - Xenobiotics Biodegradation and Metabolism - Amino Acid Metabolism	3	1
K01501	E3.5.5.1	nitrilase [EC:3.5.5.1]	Metabolism of Other Amino Acids - Xenobiotics Biodegradation and Metabolism - Amino Acid Metabolism - Energy Metabolism	1	1
K05349	bglX	beta-glucosidase [EC:3.2.1.21]	Carbohydrate Metabolism - Metabolism of Other Amino Acids - Biosynthesis of Other Secondary Metabolites	1	1
K05350	bglB	beta-glucosidase [EC:3.2.1.21]	Carbohydrate Metabolism - Metabolism of Other Amino Acids - Biosynthesis of Other Secondary Metabolites	4	2
ko00471_D-Glutamine and D-glutamate metabolism					
K00261	E1.4.1.3	glutamate dehydrogenase (NAD(P)+) [EC:1.4.1.3]	Metabolism of Other Amino Acids - Excretory System - Amino Acid Metabolism - Energy Metabolism	3	2
K01925	murD	UDP-N-acetylmuramoylalanine--D-glutamate ligase [EC:6.3.2.9]	Metabolism of Other Amino Acids - Glycan Biosynthesis and Metabolism	2	2
ko00472_D-Arginine and D-ornithine metabolism					
K00273	E1.4.3.3, DAO	D-amino-acid oxidase [EC:1.4.3.3]	Metabolism of Other Amino Acids - Biosynthesis of Other Secondary Metabolites - Transport and Catabolism - Amino Acid Metabolism	2	1
ko00473_D-Alanine metabolism					
K01921	ddl	D-alanine-D-alanine ligase [EC:6.3.2.4]	Metabolism of Other Amino Acids - Glycan Biosynthesis and Metabolism	2	2
ko00480_Glutathione metabolism					
K00033	E1.1.1.44, PGD, gnd	6-phosphogluconate dehydrogenase [EC:1.1.1.44]	Carbohydrate Metabolism - Metabolism of Other Amino Acids	20	3
K00036	G6PD, zwf	glucose-6-phosphate 1-dehydrogenase [EC:1.1.1.49]	Carbohydrate Metabolism - Metabolism of Other Amino Acids	22	2
K00432	E1.11.1.9	glutathione peroxidase [EC:1.11.1.9]	Metabolism of Other Amino Acids - Lipid Metabolism	2	2
K00797	E2.5.1.16, SRM, speE	spermidine synthase [EC:2.5.1.16]	Metabolism of Other Amino Acids - Amino Acid Metabolism	1	1
K01255	CARP, pepA	leucyl aminopeptidase [EC:3.4.11.1]	Metabolism of Other Amino Acids - Enzyme Families	3	1
K01581	E4.1.1.17, ODC1, speC, speF	ornithine decarboxylase [EC:4.1.1.17]	Metabolism of Other Amino Acids - Amino Acid Metabolism	1	1
K10807	RRM1	ribonucleoside-diphosphate reductase subunit M1 [EC:1.17.4.1]	Metabolism of Other Amino Acids - Nucleotide Metabolism - Replication and Repair	83	4
K10808	RRM2	ribonucleoside-diphosphate reductase subunit M2 [EC:1.17.4.1]	Metabolism of Other Amino Acids - Nucleotide Metabolism - Cell Growth and Death - Replication and Repair	67	5
ko00500_Starch and sucrose metabolism					
K00012	UGDH, ugd	UDPglucose 6-dehydrogenase [EC:1.1.1.22]	Carbohydrate Metabolism	91	5
K00688	E2.4.1.1, glgP, PYG	starch phosphorylase [EC:2.4.1.1]	Carbohydrate Metabolism - Endocrine System	9	3
K00697	E2.4.1.15, otsA	alpha,alpha-trehalose-phosphate synthase (UDP-forming) [EC:2.4.1.15]	Carbohydrate Metabolism - Glycan Biosynthesis and Metabolism	3	1
K00705	malQ	4-alpha-glucanotransferase [EC:2.4.1.25]	Carbohydrate Metabolism	1	1
K00845	glk	glucokinase [EC:2.7.1.2]	Carbohydrate Metabolism - Biosynthesis of Other Secondary Metabolites	2	2
K00847	E2.7.1.4, scrK	fructokinase [EC:2.7.1.4]	Carbohydrate Metabolism	1	1
K00978	rfbF	glucose-1-phosphate cytidylyltransferase [EC:2.7.7.33]	Carbohydrate Metabolism	6	1
K01176	E3.2.1.1, amyA, malS	alpha-amylase [EC:3.2.1.1]	Carbohydrate Metabolism - Digestive System	2	1
K01179	E3.2.1.4	endoglucanase [EC:3.2.1.4]	Carbohydrate Metabolism	11	2
K01187	E3.2.1.20, malZ	alpha-glucosidase [EC:3.2.1.20]	Carbohydrate Metabolism	1	1
K01193	E3.2.1.26, sacA	beta-fructofuranosidase [EC:3.2.1.26]	Carbohydrate Metabolism	1	1
K01225	E3.2.1.91	cellulose 1,4-beta-cellobiosidase [EC:3.2.1.91]	Carbohydrate Metabolism	7	2
K01810	GPI, pgi	glucose-6-phosphate isomerase [EC:5.3.1.9]	Carbohydrate Metabolism	2	2
K02791	PTS-Mal-EIIC, malX	PTS system, maltose and glucose-specific IIC component	Carbohydrate Metabolism - Membrane Transport	1	1
K05349	bglX	beta-glucosidase [EC:3.2.1.21]	Carbohydrate Metabolism - Metabolism of Other Amino Acids - Biosynthesis of Other Secondary Metabolites	1	1
K05350	bglB	beta-glucosidase [EC:3.2.1.21]	Carbohydrate Metabolism - Metabolism of Other Amino Acids - Biosynthesis of Other Secondary Metabolites	4	2
ko00510_N-Glycan biosynthesis					
K00721	DPM1	dolichol-phosphate mannosyltransferase [EC:2.4.1.83]	Glycan Biosynthesis and Metabolism	39	3
K00737	MGAT3	beta-1,4-mannosyl-glycoprotein beta-1,4-N-acetylglucosaminyltransferase [EC:2.4.1.144]	Glycan Biosynthesis and Metabolism	23	3
ko00511_Other glycan degradation					
K01190	lacZ	beta-galactosidase [EC:3.2.1.23]	Carbohydrate Metabolism - Glycan Biosynthesis and Metabolism - Lipid Metabolism	1	1
K01191	E3.2.1.24	alpha-mannosidase [EC:3.2.1.24]	Glycan Biosynthesis and Metabolism	2	1
K01206	E3.2.1.51, FUCA	alpha-L-fucosidase [EC:3.2.1.51]	Glycan Biosynthesis and Metabolism	1	1
K01227	E3.2.1.96	mannosyl-glycoprotein endo-beta-N-acetylglucosaminidase [EC:3.2.1.96]	Glycan Biosynthesis and Metabolism	8	2
K12373	HEXA_B	hexosaminidase [EC:3.2.1.52]	Carbohydrate Metabolism - Folding, Sorting and Degradation - Glycan Biosynthesis and Metabolism - Transport and Catabolism	1	1
ko00520_Amino sugar and nucleotide sugar metabolism					
K00012	UGDH, ugd	UDPglucose 6-dehydrogenase [EC:1.1.1.22]	Carbohydrate Metabolism	91	5
K00066	algD	GDP-mannose 6-dehydrogenase [EC:1.1.1.132]	Carbohydrate Metabolism - Signal Transduction	3	1
K00523	ascD, ddhD, rfbI	CDP-4-dehydro-6-deoxyglucose reductase [EC:1.17.1.1]	Carbohydrate Metabolism	7	2
K00790	murA	UDP-N-acetylglucosamine 1-carboxyvinyltransferase [EC:2.5.1.7]	Carbohydrate Metabolism - Glycan Biosynthesis and Metabolism	12	2
K00820	E2.6.1.16, glmS	glucosamine--fructose-6-phosphate aminotransferase (isomerizing) [EC:2.6.1.16]	Carbohydrate Metabolism - Enzyme Families - Amino Acid Metabolism	20	4
K00845	glk	glucokinase [EC:2.7.1.2]	Carbohydrate Metabolism - Biosynthesis of Other Secondary Metabolites	2	2
K00847	E2.7.1.4, scrK	fructokinase [EC:2.7.1.4]	Carbohydrate Metabolism	1	1
K00966	GMPP	mannose-1-phosphate guanylyltransferase [EC:2.7.7.13]	Carbohydrate Metabolism	4	2
K00971	E2.7.7.22, manC	mannose-1-phosphate guanylyltransferase [EC:2.7.7.22]	Carbohydrate Metabolism	12	4
K00978	rfbF	glucose-1-phosphate cytidylyltransferase [EC:2.7.7.33]	Carbohydrate Metabolism	6	1

K00983	E2.7.7.43, neuA, CMAS	N-acylneuraminate cytidylyltransferase [EC:2.7.7.43]	Carbohydrate Metabolism	11	5
K01183	E3.2.1.14	chitinase [EC:3.2.1.14]	Carbohydrate Metabolism	1	1
K01207	E3.2.1.52, nagZ	beta-N-acetylhexosaminidase [EC:3.2.1.52]	Carbohydrate Metabolism	1	1
K01209	E3.2.1.55, abfA	alpha-N-arabinofuranosidase [EC:3.2.1.55]	Carbohydrate Metabolism	1	1
K01654	E2.5.1.56, neuB	N-acetylneuraminate synthase [EC:2.5.1.56]	Carbohydrate Metabolism	126	8
K01709	rfbG	CDP-glucose 4,6-dehydratase [EC:4.2.1.45]	Carbohydrate Metabolism	6	3
K01711	E4.2.1.47, gmd	GDPmannose 4,6-dehydratase [EC:4.2.1.47]	Carbohydrate Metabolism	533	7
K01784	galE, GALE	UDP-glucose 4-epimerase [EC:5.1.3.2]	Carbohydrate Metabolism	175	8
K01791	wecB	UDP-N-acetylglucosamine 2-epimerase [EC:5.1.3.14]	Carbohydrate Metabolism - Glycan Biosynthesis and Metabolism	38	7
K01809	E5.3.1.8, manA	mannose-6-phosphate isomerase [EC:5.3.1.8]	Carbohydrate Metabolism	17	3
K01810	GPI, pgi	glucose-6-phosphate isomerase [EC:5.3.1.9]	Carbohydrate Metabolism	2	2
K01840	E5.4.2.8, manB	phosphomannomutase [EC:5.4.2.8]	Carbohydrate Metabolism	1	1
K02377	E1.1.1.271, fcl	GDP-L-fucose synthase [EC:1.1.1.271]	Carbohydrate Metabolism	251	7
K02472	wecC	UDP-N-acetyl-D-mannosaminuronic acid dehydrogenase [EC:1.1.1.-]	Carbohydrate Metabolism	1	1
K02473	E5.1.3.7, wbpP	UDP-N-acetylglucosamine 4-epimerase [EC:5.1.3.7]	Carbohydrate Metabolism	2	2
K02564	nagB, GNPDA	glucosamine-6-phosphate deaminase [EC:3.5.99.6]	Carbohydrate Metabolism	2	2
K02795	PTS-Man-EIIC, manY	PTS system, mannose-specific IIC component	Carbohydrate Metabolism - Membrane Transport	1	1
K03431	glmM	phosphoglucosamine mutase [EC:5.4.2.10]	Carbohydrate Metabolism	5	2
K04042	glmU	bifunctional UDP-N-acetylglucosamine pyrophosphorylase / Glucosamine-1-phosphate N-acetyltransferase [EC:2.7.7.23 2.3.1.157]	Carbohydrate Metabolism	2	2
K05304	NANS, SAS	sialic acid synthase [EC:2.5.1.56 2.5.1.57]	Carbohydrate Metabolism	1	1
K06118	E3.13.1.1, sqd1, sqdb	UDP-sulfoquinovose synthase [EC:3.13.1.1]	Carbohydrate Metabolism - Lipid Metabolism	2	2
K07106	murQ	N-acetylmuramic acid 6-phosphate etherase [EC:4.2.-.-]	Carbohydrate Metabolism	1	1
K07806	arnB, pmrH	UDP-4-amino-4-deoxy-L-arabinose-oxoglutarate aminotransferase [EC:2.6.1.87]	Carbohydrate Metabolism - Signal Transduction - Glycan Biosynthesis and Metabolism - Amino Acid Metabolism	3	2
K11528	glmU1	UDP-N-acetylglucosamine pyrophosphorylase [EC:2.7.7.23]	Carbohydrate Metabolism	1	1
K12373	HEXA_B	hexosaminidase [EC:3.2.1.52]	Carbohydrate Metabolism - Folding, Sorting and Degradation - Glycan Biosynthesis and Metabolism - Transport and Catabolism	1	1
K12410	npdA	NAD-dependent deacetylase [EC:3.5.1.-]	Carbohydrate Metabolism	4	1
K12452	rfbH	CDP-6-deoxy-D-xylo-4-hexulose-3-dehydrase	Carbohydrate Metabolism	329	6
K12454	rfbE	CDP-paratose 2-epimerase [EC:5.1.3.10]	Carbohydrate Metabolism	19	4
ko00521_Streptomycin biosynthesis					
K00067	rfbD	dTDP-4-dehydrorhamnose reductase [EC:1.1.1.133]	Biosynthesis of Other Secondary Metabolites - Metabolism of Terpenoids and Polyketides	39	4
K00845	glk	glucokinase [EC:2.7.1.2]	Carbohydrate Metabolism - Biosynthesis of Other Secondary Metabolites	2	2
K00973	E2.7.7.24, rfbA, rffH	glucose-1-phosphate thymidylyltransferase [EC:2.7.7.24]	Biosynthesis of Other Secondary Metabolites - Metabolism of Terpenoids and Polyketides	28	5
K01092	E3.1.3.25, IMPA, suhB	myo-inositol-1(or 4)-monophosphatase [EC:3.1.3.25]	Carbohydrate Metabolism - Signal Transduction - Biosynthesis of Other Secondary Metabolites	3	2
K01710	E4.2.1.46, rfbB, rffG	dTDP-glucose 4,6-dehydratase [EC:4.2.1.46]	Biosynthesis of Other Secondary Metabolites - Metabolism of Terpenoids and Polyketides	341	7
K01790	rfbC	dTDP-4-dehydrorhamnose 3,5-epimerase [EC:5.1.3.13]	Biosynthesis of Other Secondary Metabolites - Metabolism of Terpenoids and Polyketides	89	4
K01858	E5.5.1.4, INO1	myo-inositol-1-phosphate synthase [EC:5.5.1.4]	Carbohydrate Metabolism - Biosynthesis of Other Secondary Metabolites	1	1
K04340	strB1	scyllo-inosamine-4-phosphate amidinotransferase 1 [EC:2.1.4.2]	Biosynthesis of Other Secondary Metabolites	16	2
ko00523_Polyketide sugar unit biosynthesis					
K00067	rfbD	dTDP-4-dehydrorhamnose reductase [EC:1.1.1.133]	Biosynthesis of Other Secondary Metabolites - Metabolism of Terpenoids and Polyketides	39	4
K00973	E2.7.7.24, rfbA, rffH	glucose-1-phosphate thymidylyltransferase [EC:2.7.7.24]	Biosynthesis of Other Secondary Metabolites - Metabolism of Terpenoids and Polyketides	28	5
K01710	E4.2.1.46, rfbB, rffG	dTDP-glucose 4,6-dehydratase [EC:4.2.1.46]	Biosynthesis of Other Secondary Metabolites - Metabolism of Terpenoids and Polyketides	341	7
K01790	rfbC	dTDP-4-dehydrorhamnose 3,5-epimerase [EC:5.1.3.13]	Biosynthesis of Other Secondary Metabolites - Metabolism of Terpenoids and Polyketides	89	4
ko00524_Butirosin and neomycin biosynthesis					
K00845	glk	glucokinase [EC:2.7.1.2]	Carbohydrate Metabolism - Biosynthesis of Other Secondary Metabolites	2	2
ko00531_Glycosaminoglycan degradation					
K12373	HEXA_B	hexosaminidase [EC:3.2.1.52]	Carbohydrate Metabolism - Folding, Sorting and Degradation - Glycan Biosynthesis and Metabolism - Transport and Catabolism	1	1
ko00540_Lipopolysaccharide biosynthesis					
K00677	lpxA	UDP-N-acetylglucosamine acyltransferase [EC:2.3.1.129]	Glycan Biosynthesis and Metabolism	137	3
K00979	kdsB	3-deoxy-manno-octulosonate cytidylyltransferase (CMP-KDO synthetase) [EC:2.7.7.38]	Glycan Biosynthesis and Metabolism	4	2
K01627	kdsA	2-dehydro-3-deoxyphosphooctonate aldolase (KDO 8-P synthase) [EC:2.5.1.55]	Glycan Biosynthesis and Metabolism	24	5
K02535	lpxC	UDP-3-O-[3-hydroxymyristoyl] N-acetylglucosamine deacetylase [EC:3.5.1.-]	Glycan Biosynthesis and Metabolism	5	2
K02536	lpxD	UDP-3-O-[3-hydroxymyristoyl] glucosamine N-acyltransferase [EC:2.3.1.-]	Glycan Biosynthesis and Metabolism	12	2
K02841	waaC, rfaC	heptosyltransferase I [EC:2.4.-.-]	Glycan Biosynthesis and Metabolism	3	2
K02843	waaF, rfaF	heptosyltransferase II [EC:2.4.-.-]	Glycan Biosynthesis and Metabolism	18	2
K02849	waaQ, rfaQ	heptosyltransferase III [EC:2.4.-.-]	Glycan Biosynthesis and Metabolism	1	1
K03270	kdsC	3-deoxy-D-manno-octulosonate 8-phosphate phosphatase (KDO 8-P phosphatase) [EC:3.1.3.45]	Glycan Biosynthesis and Metabolism	6	1
K03271	gmhA	phosphoheptose isomerase [EC:5.-.-.-]	Glycan Biosynthesis and Metabolism	4	3
K03272	gmhC, hldE, waaE, rfaE	D-beta-D-heptose 7-phosphate kinase / D-beta-D-heptose 1-phosphate adenosyltransferase [EC:2.7.1.- 2.7.7.-]	Glycan Biosynthesis and Metabolism	71	4
K03273	gmhB	D-glycero-D-manno-heptose 1,7-bisphosphate phosphatase [EC:3.1.3.-]	Glycan Biosynthesis and Metabolism	1	1
K03274	rfaD	ADP-L-glycero-D-manno-heptose 6-epimerase [EC:5.1.3.20]	Glycan Biosynthesis and Metabolism	46	3
ko00550_Peptidoglycan biosynthesis					
K00790	murA	UDP-N-acetylglucosamine 1-carboxyvinyltransferase [EC:2.5.1.7]	Carbohydrate Metabolism - Glycan Biosynthesis and Metabolism	12	2
K01000	mraY	phospho-N-acetylmuramoyl-pentapeptide-transferase [EC:2.7.8.13]	Glycan Biosynthesis and Metabolism	2	1
K01921	ddl	D-alanine-D-alanine ligase [EC:6.3.2.4]	Metabolism of Other Amino Acids - Glycan Biosynthesis and Metabolism	2	2
K01925	murD	UDP-N-acetylmuramoylalanine--D-glutamate ligase [EC:6.3.2.9]	Metabolism of Other Amino Acids - Glycan Biosynthesis and Metabolism	2	2
K01929	murF	UDP-N-acetylmuramoylalanyl-D-glutamyl-2,6-diaminopimelate--D-alanyl-D-alanine ligase [EC:6.3.2.10]	Glycan Biosynthesis and Metabolism - Amino Acid Metabolism	1	1
K03587	ftsI	cell division protein FtsI (penicillin-binding protein 3) [EC:2.4.1.129]	Glycan Biosynthesis and Metabolism - Replication and Repair	1	1
K05366	mrcA	penicillin-binding protein 1A [EC:2.4.1.- 3.4.-.-]	Glycan Biosynthesis and Metabolism	1	1
K05367	pbpC	penicillin-binding protein 1C [EC:2.4.1.-]	Glycan Biosynthesis and Metabolism	2	1
K06153	E3.6.1.27, bacA	undecaprenyl-diphosphatase [EC:3.6.1.27]	Glycan Biosynthesis and Metabolism	1	1
K07260	vanY	D-alanyl-D-alanine carboxypeptidase [EC:3.4.16.4]	Enzyme Families - Glycan Biosynthesis and Metabolism	6	2
ko00561_Glycerolipid metabolism					
K00005	E1.1.1.6, gldA	glycerol dehydrogenase [EC:1.1.1.6]	Lipid Metabolism	1	1
K00128	E1.2.1.3	aldehyde dehydrogenase (NAD+) [EC:1.2.1.3]	Carbohydrate Metabolism - Metabolism of Other Amino Acids - Xenobiotics Biodegradation and Metabolism - Metabolism of Terpenoids and Polyketides - Amino Acid Metabolism - Lipid Metabolism	4	4
K00655	plsC	1-acyl-sn-glycerol-3-phosphate acyltransferase [EC:2.3.1.51]	Lipid Metabolism	8	2
K00864	E2.7.1.30, glpK	glycerol kinase [EC:2.7.1.30]	Environmental Adaptation - Endocrine System - Lipid Metabolism	3	3
K01046	E3.1.1.3	triacylglycerol lipase [EC:3.1.1.3]	Lipid Metabolism	7	3
K03429	ugtP	1,2-diacylglycerol 3-glucosyltransferase [EC:2.4.1.157]	Glycan Biosynthesis and Metabolism - Lipid Metabolism	8	1
K06118	E3.13.1.1, sqd1, sqdb	UDP-sulfoquinovose synthase [EC:3.13.1.1]	Carbohydrate Metabolism - Lipid Metabolism	2	2
ko00562_Inositol phosphate metabolism					
K00140	E1.2.1.27, mmsA, iolA	methylmalonate-semialdehyde dehydrogenase [EC:1.2.1.27]	Carbohydrate Metabolism - Amino Acid Metabolism	1	1
K01092	E3.1.3.25, IMPA, suhB	myo-inositol-1(or 4)-monophosphatase [EC:3.1.3.25]	Carbohydrate Metabolism - Signal Transduction - Biosynthesis of Other Secondary Metabolites	3	2
K01858	E5.5.1.4, INO1	myo-inositol-1-phosphate synthase [EC:5.5.1.4]	Carbohydrate Metabolism - Biosynthesis of Other Secondary Metabolites	1	1
K03338	iolC	5-dehydro-2-deoxygluconokinase [EC:2.7.1.92]	Carbohydrate Metabolism	1	1
ko00564_Glycerophospholipid metabolism					
K00057	gpsA	glycerol-3-phosphate dehydrogenase (NAD(P)+) [EC:1.1.1.94]	Lipid Metabolism	2	2

K00111	glpA, glpD	glycerol-3-phosphate dehydrogenase [EC:1.1.5.3]	Lipid Metabolism	1	1
K00655	plsC	1-acyl-sn-glycerol-3-phosphate acyltransferase [EC:2.3.1.51]	Lipid Metabolism	8	2
K00968	E2.7.7.15, PCYT1	choline-phosphate cytidylyltransferase [EC:2.7.7.15]	Metabolism of Other Amino Acids - Lipid Metabolism	12	3
K00980	tagD	glycerol-3-phosphate cytidylyltransferase [EC:2.7.7.39]	Lipid Metabolism	86	6
K00995	E2.7.8.5, pgsA	CDP-diacylglycerol--glycerol-3-phosphate 3-phosphatidyltransferase [EC:2.7.8.5]	Lipid Metabolism	1	1
K00998	E2.7.8.8, pssA	phosphatidylserine synthase [EC:2.7.8.8]	Amino Acid Metabolism - Lipid Metabolism	2	2
K01096	pgpB	phosphatidylglycerophosphatase B [EC:3.1.3.27]	Lipid Metabolism	1	1
K01613	E4.1.1.65, psd	phosphatidylserine decarboxylase [EC:4.1.1.65]	Lipid Metabolism	27	4
ko00590_Arachidonic acid metabolism					
K00432	E1.11.1.9	glutathione peroxidase [EC:1.11.1.9]	Metabolism of Other Amino Acids - Lipid Metabolism	2	2
ko00591_Linoleic acid metabolism					
K00100	E1.1.1.-		Carbohydrate Metabolism - Xenobiotics Biodegradation and Metabolism - Lipid Metabolism	1	1
ko00600_Sphingolipid metabolism					
K01190	lacZ	beta-galactosidase [EC:3.2.1.23]	Carbohydrate Metabolism - Glycan Biosynthesis and Metabolism - Lipid Metabolism	1	1
ko00603_Glycosphingolipid biosynthesis - globo series					
K12373	HEXA_B	hexosaminidase [EC:3.2.1.52]	Carbohydrate Metabolism - Folding, Sorting and Degradation - Glycan Biosynthesis and Metabolism - Transport and Catabolism	1	1
ko00604_Glycosphingolipid biosynthesis - ganglio series					
K12373	HEXA_B	hexosaminidase [EC:3.2.1.52]	Carbohydrate Metabolism - Folding, Sorting and Degradation - Glycan Biosynthesis and Metabolism - Transport and Catabolism	1	1
ko00620_Pyruvate metabolism					
K00024	mdh	malate dehydrogenase [EC:1.1.1.37]	Carbohydrate Metabolism - Energy Metabolism	3	3
K00027	E1.1.1.38, sfcA, maeA	malate dehydrogenase (oxaloacetate-decarboxylating) [EC:1.1.1.38]	Carbohydrate Metabolism - Signal Transduction	2	1
K00029	E1.1.1.40, maeB	malate dehydrogenase (oxaloacetate-decarboxylating)(NADP+) [EC:1.1.1.40]	Carbohydrate Metabolism - Energy Metabolism	2	1
K00101	E1.1.2.3, lldD	L-lactate dehydrogenase (cytochrome) [EC:1.1.2.3]	Carbohydrate Metabolism	1	1
K00128	E1.2.1.3	aldehyde dehydrogenase (NAD+) [EC:1.2.1.3]	Carbohydrate Metabolism - Metabolism of Other Amino Acids - Xenobiotics Biodegradation and Metabolism - Metabolism of Terpenoids and Polyketides - Amino Acid Metabolism - Lipid Metabolism	4	4
K00156	poxB	pyruvate dehydrogenase (quinone) [EC:1.2.5.1]	Carbohydrate Metabolism	3	2
K00161	PDHA, pdhA	pyruvate dehydrogenase E1 component subunit alpha [EC:1.2.4.1]	Carbohydrate Metabolism - Amino Acid Metabolism	57	3
K00162	PDHB, pdhB	pyruvate dehydrogenase E1 component subunit beta [EC:1.2.4.1]	Carbohydrate Metabolism - Amino Acid Metabolism	37	3
K00382	DLD, lpd, pdhD	dihydrolipoamide dehydrogenase [EC:1.8.1.4]	Carbohydrate Metabolism - Amino Acid Metabolism	1	1
K00626	E2.3.1.9, atoB	acetyl-CoA C-acetyltransferase [EC:2.3.1.9]	Carbohydrate Metabolism - Signal Transduction - Xenobiotics Biodegradation and Metabolism - Metabolism of Terpenoids and Polyketides - Amino Acid Metabolism - Lipid Metabolism - Energy Metabolism	4	3
K00627	DLAT, aceF, pdhC	pyruvate dehydrogenase E2 component (dihydrolipoamide acetyltransferase) [EC:2.3.1.12]	Carbohydrate Metabolism	1	1
K01006	ppdK	pyruvate,orthophosphate dikinase [EC:2.7.9.1]	Carbohydrate Metabolism - Energy Metabolism	1	1
K01007	pps, ppsA	pyruvate, water dikinase [EC:2.7.9.2]	Carbohydrate Metabolism - Energy Metabolism	107	4
K01595	ppc	phosphoenolpyruvate carboxylase [EC:4.1.1.31]	Carbohydrate Metabolism - Energy Metabolism	1	1
K01610	E4.1.1.49, pckA	phosphoenolpyruvate carboxykinase (ATP) [EC:4.1.1.49]	Carbohydrate Metabolism - Energy Metabolism	4	2
K01638	E2.3.3.9, aceB, glcB	malate synthase [EC:2.3.3.9]	Carbohydrate Metabolism	9	3
K01649	E2.3.3.13, leuA	2-isopropylmalate synthase [EC:2.3.3.13]	Carbohydrate Metabolism - Amino Acid Metabolism	1	1
K01734	E4.2.3.3, mgsA	methylglyoxal synthase [EC:4.2.3.3]	Carbohydrate Metabolism	30	3
K01759	E4.4.1.5, GLO1, gloA	lactoylglutathione lyase [EC:4.4.1.5]	Carbohydrate Metabolism - Signal Transduction	8	1
K01895	ACSS, acs	acetyl-CoA synthetase [EC:6.2.1.1]	Carbohydrate Metabolism - Lipid Metabolism - Energy Metabolism	1	1
K01962	accA	acetyl-CoA carboxylase carboxyl transferase subunit alpha [EC:6.4.1.2]	Carbohydrate Metabolism - Metabolism of Terpenoids and Polyketides - Lipid Metabolism - Energy Metabolism	1	1
K01963	accD	acetyl-CoA carboxylase carboxyl transferase subunit beta [EC:6.4.1.2]	Carbohydrate Metabolism - Metabolism of Terpenoids and Polyketides - Lipid Metabolism - Energy Metabolism	2	2
K03778	ldhA	D-lactate dehydrogenase [EC:1.1.1.28]	Carbohydrate Metabolism	1	1
K04073	mhpF	acetaldehyde dehydrogenase [EC:1.2.1.10]	Carbohydrate Metabolism - Xenobiotics Biodegradation and Metabolism	43	2
ko00621_Dioxin degradation					
K01666	E4.1.3.39, mhpE	4-hydroxy 2-oxovalerate aldolase [EC:4.1.3.39]	Xenobiotics Biodegradation and Metabolism - Amino Acid Metabolism	35	3
K02554	mhpD	2-keto-4-pentenoate hydratase [EC:4.2.1.80]	Xenobiotics Biodegradation and Metabolism - Amino Acid Metabolism	1	1
K04073	mhpF	acetaldehyde dehydrogenase [EC:1.2.1.10]	Carbohydrate Metabolism - Xenobiotics Biodegradation and Metabolism	43	2
ko00622_Xylene degradation					
K01666	E4.1.3.39, mhpE	4-hydroxy 2-oxovalerate aldolase [EC:4.1.3.39]	Xenobiotics Biodegradation and Metabolism - Amino Acid Metabolism	35	3
K02554	mhpD	2-keto-4-pentenoate hydratase [EC:4.2.1.80]	Xenobiotics Biodegradation and Metabolism - Amino Acid Metabolism	1	1
K04073	mhpF	acetaldehyde dehydrogenase [EC:1.2.1.10]	Carbohydrate Metabolism - Xenobiotics Biodegradation and Metabolism	43	2
ko00623_Toluene degradation					
K00239	sdhA	succinate dehydrogenase flavoprotein subunit [EC:1.3.99.1]	Carbohydrate Metabolism - Xenobiotics Biodegradation and Metabolism - Energy Metabolism	2	2
K00240	sdhB	succinate dehydrogenase iron-sulfur protein [EC:1.3.99.1]	Carbohydrate Metabolism - Xenobiotics Biodegradation and Metabolism - Energy Metabolism	18	1
ko00624_Polycyclic aromatic hydrocarbon degradation					
K00599	E2.1.1.-		Xenobiotics Biodegradation and Metabolism - Amino Acid Metabolism	55	3
ko00625_Chloroalkane and chloroalkene degradation					
K00001	E1.1.1.1, adh	alcohol dehydrogenase [EC:1.1.1.1]	Carbohydrate Metabolism - Metabolism of Cofactors and Vitamins - Xenobiotics Biodegradation and Metabolism - Amino Acid Metabolism - Lipid Metabolism	1	1
K00100	E1.1.1.-		Carbohydrate Metabolism - Xenobiotics Biodegradation and Metabolism - Lipid Metabolism	1	1
K00121	frmA, ADH5, adhC	S-(hydroxymethyl)glutathione dehydrogenase / alcohol dehydrogenase [EC:1.1.1.284 1.1.1.1]	Carbohydrate Metabolism - Metabolism of Cofactors and Vitamins - Xenobiotics Biodegradation and Metabolism - Amino Acid Metabolism - Lipid Metabolism - Energy Metabolism	8	3
K00128	E1.2.1.3	aldehyde dehydrogenase (NAD+) [EC:1.2.1.3]	Carbohydrate Metabolism - Metabolism of Other Amino Acids - Xenobiotics Biodegradation and Metabolism - Metabolism of Terpenoids and Polyketides - Amino Acid Metabolism - Lipid Metabolism	4	4
K01563	dhaA	haloalkane dehalogenase [EC:3.8.1.5]	Xenobiotics Biodegradation and Metabolism	1	1
K13953	adhP	alcohol dehydrogenase, propanol-preferring [EC:1.1.1.1]	Carbohydrate Metabolism - Metabolism of Cofactors and Vitamins - Xenobiotics Biodegradation and Metabolism - Amino Acid Metabolism - Lipid Metabolism	1	1
ko00626_Naphthalene degradation					
K00001	E1.1.1.1, adh	alcohol dehydrogenase [EC:1.1.1.1]	Carbohydrate Metabolism - Metabolism of Cofactors and Vitamins - Xenobiotics Biodegradation and Metabolism - Amino Acid Metabolism - Lipid Metabolism	1	1
K00121	frmA, ADH5, adhC	S-(hydroxymethyl)glutathione dehydrogenase / alcohol dehydrogenase [EC:1.1.1.284 1.1.1.1]	Carbohydrate Metabolism - Metabolism of Cofactors and Vitamins - Xenobiotics Biodegradation and Metabolism - Amino Acid Metabolism - Lipid Metabolism - Energy Metabolism	8	3
K00257	E1.3.99.-		Xenobiotics Biodegradation and Metabolism - Metabolism of Terpenoids and Polyketides	2	2
K00680	E2.3.1.-		Xenobiotics Biodegradation and Metabolism - Metabolism of Terpenoids and Polyketides - Amino Acid Metabolism	9	1
K01726	E4.2.1.-		Carbohydrate Metabolism - Xenobiotics Biodegradation and Metabolism - Metabolism of Terpenoids and Polyketides	1	1
K13953	adhP	alcohol dehydrogenase, propanol-preferring [EC:1.1.1.1]	Carbohydrate Metabolism - Metabolism of Cofactors and Vitamins - Xenobiotics Biodegradation and Metabolism - Amino Acid Metabolism - Lipid Metabolism	1	1
ko00627_Aminobenzoate degradation					
K00680	E2.3.1.-		Xenobiotics Biodegradation and Metabolism - Metabolism of Terpenoids and Polyketides - Amino Acid Metabolism	9	1
K01426	E3.5.1.4, amiE	amidase [EC:3.5.1.4]	Metabolism of Other Amino Acids - Xenobiotics Biodegradation and Metabolism - Amino Acid Metabolism	3	1
K01501	E3.5.5.1	nitrilase [EC:3.5.5.1]	Metabolism of Other Amino Acids - Xenobiotics Biodegradation and Metabolism - Amino Acid Metabolism - Energy Metabolism	1	1

	K01692	E4.2.1.17, paaG	enoyl-CoA hydratase [EC:4.2.1.17]	Carbohydrate Metabolism - Metabolism of Other Amino Acids - Xenobiotics Biodegradation and Metabolism - Metabolism of Terpenoids and Polyketides - Amino Acid Metabolism - Lipid Metabolism	33	3
	K09474	phoN	acid phosphatase (class A) [EC:3.1.3.2]	Signal Transduction - Metabolism of Cofactors and Vitamins - Xenobiotics Biodegradation and Metabolism	8	2
ko00630_Glyoxylate and dicarboxylate metabolism						
	K00023	E1.1.1.36, phbB	acetoacetyl-CoA reductase [EC:1.1.1.36]	Carbohydrate Metabolism	3	2
	K00024	mdh	malate dehydrogenase [EC:1.1.1.37]	Carbohydrate Metabolism - Energy Metabolism	3	3
	K00050	E1.1.1.81, ttuD	hydroxypyruvate reductase [EC:1.1.1.81]	Carbohydrate Metabolism - Amino Acid Metabolism	1	1
	K00104	glcD	glycolate oxidase [EC:1.1.3.15]	Carbohydrate Metabolism	1	1
	K00123	E1.2.1.2A	formate dehydrogenase, alpha subunit [EC:1.2.1.2]	Carbohydrate Metabolism - Energy Metabolism	2	2
	K00626	E2.3.1.9, atoB	acetyl-CoA C-acetyltransferase [EC:2.3.1.9]	Carbohydrate Metabolism - Signal Transduction - Xenobiotics Biodegradation and Metabolism - Metabolism of Terpenoids and Polyketides - Amino Acid Metabolism - Lipid Metabolism - Energy Metabolism	4	3
	K01091	E3.1.3.18, gph	phosphoglycolate phosphatase [EC:3.1.3.18]	Carbohydrate Metabolism	1	1
	K01433	purU	formyltetrahydrofolate deformylase [EC:3.5.1.10]	Carbohydrate Metabolism - Metabolism of Cofactors and Vitamins	1	1
	K01601	rbcL	ribulose-bisphosphate carboxylase large chain [EC:4.1.1.39]	Carbohydrate Metabolism - Energy Metabolism	6	3
	K01637	E4.1.3.1, aceA	isocitrate lyase [EC:4.1.3.1]	Carbohydrate Metabolism	9	3
	K01638	E2.3.3.9, aceB, glcB	malate synthase [EC:2.3.3.9]	Carbohydrate Metabolism	9	3
	K01681	ACO, acnA	aconitate hydratase 1 [EC:4.2.1.3]	Carbohydrate Metabolism - Energy Metabolism	4	3
	K01847	MUT	methylmalonyl-CoA mutase [EC:5.4.99.2]	Carbohydrate Metabolism - Amino Acid Metabolism - Energy Metabolism	2	2
	K11472	glcE	glycolate oxidase FAD binding subunit	Carbohydrate Metabolism	2	1
ko00633_Nitrotoluene degradation						
	K06281	E1.12.99.6L	hydrogenase large subunit [EC:1.12.99.6]	Xenobiotics Biodegradation and Metabolism	1	1
	K11180	dsrA	sulfite reductase, dissimilatory-type alpha subunit [EC:1.8.99.3]	Xenobiotics Biodegradation and Metabolism	2	1
ko00640_Propanoate metabolism						
	K00128	E1.2.1.3	aldehyde dehydrogenase (NAD+) [EC:1.2.1.3]	Carbohydrate Metabolism - Metabolism of Other Amino Acids - Xenobiotics Biodegradation and Metabolism - Metabolism of Terpenoids and Polyketides - Amino Acid Metabolism - Lipid Metabolism	4	4
	K00140	E1.2.1.27, mmsA, iolA	methylmalonate-semialdehyde dehydrogenase [EC:1.2.1.27]	Carbohydrate Metabolism - Amino Acid Metabolism	1	1
	K00249	E1.3.99.3, ACADM, acd	acyl-CoA dehydrogenase [EC:1.3.99.3]	Carbohydrate Metabolism - Metabolism of Other Amino Acids - Endocrine System - Amino Acid Metabolism - Lipid Metabolism	1	1
	K00626	E2.3.1.9, atoB	acetyl-CoA C-acetyltransferase [EC:2.3.1.9]	Carbohydrate Metabolism - Signal Transduction - Xenobiotics Biodegradation and Metabolism - Metabolism of Terpenoids and Polyketides - Amino Acid Metabolism - Lipid Metabolism - Energy Metabolism	4	3
	K00823	puuE	4-aminobutyrate aminotransferase [EC:2.6.1.19]	Carbohydrate Metabolism - Metabolism of Other Amino Acids - Amino Acid Metabolism	1	1
	K01604	mmdA	methylmalonyl-CoA decarboxylase alpha chain [EC:4.1.1.41]	Carbohydrate Metabolism	1	1
	K01659	E2.3.3.5, prpC	2-methylcitrate synthase [EC:2.3.3.5]	Carbohydrate Metabolism	1	1
	K01692	E4.2.1.17, paaG	enoyl-CoA hydratase [EC:4.2.1.17]	Carbohydrate Metabolism - Metabolism of Other Amino Acids - Xenobiotics Biodegradation and Metabolism - Metabolism of Terpenoids and Polyketides - Amino Acid Metabolism - Lipid Metabolism	33	3
	K01825	fadB	3-hydroxyacyl-CoA dehydrogenase / enoyl-CoA hydratase / 3-hydroxybutyryl-CoA epimerase / enoyl-CoA isomerase [EC:1.1.1.35 4.2.1.17 5.1.2.3 5.3.3.8]	Carbohydrate Metabolism - Metabolism of Other Amino Acids - Xenobiotics Biodegradation and Metabolism - Metabolism of Terpenoids and Polyketides - Amino Acid Metabolism - Lipid Metabolism - Energy Metabolism	1	1
	K01847	MUT	methylmalonyl-CoA mutase [EC:5.4.99.2]	Carbohydrate Metabolism - Amino Acid Metabolism - Energy Metabolism	2	2
	K01848	E5.4.99.2A, mcmA1	methylmalonyl-CoA mutase, N-terminal domain [EC:5.4.99.2]	Carbohydrate Metabolism - Amino Acid Metabolism - Energy Metabolism	1	1
	K01895	ACSS, acs	acetyl-CoA synthetase [EC:6.2.1.1]	Carbohydrate Metabolism - Lipid Metabolism - Energy Metabolism	1	1
	K01962	accA	acetyl-CoA carboxylase carboxyl transferase subunit alpha [EC:6.4.1.2]	Carbohydrate Metabolism - Metabolism of Terpenoids and Polyketides - Lipid Metabolism - Energy Metabolism	1	1
	K01963	accD	acetyl-CoA carboxylase carboxyl transferase subunit beta [EC:6.4.1.2]	Carbohydrate Metabolism - Metabolism of Terpenoids and Polyketides - Lipid Metabolism - Energy Metabolism	2	2
	K07250	gabT	4-aminobutyrate aminotransferase / (S)-3-amino-2-methylpropionate transaminase [EC:2.6.1.19 2.6.1.22]	Carbohydrate Metabolism - Metabolism of Other Amino Acids - Amino Acid Metabolism	1	1
ko00642_Ethylbenzene degradation						
	K00529	hcaD	ferredoxin--NAD+ reductase [EC:1.18.1.3]	Xenobiotics Biodegradation and Metabolism - Amino Acid Metabolism - Lipid Metabolism	1	1
	K00680	E2.3.1.-		Xenobiotics Biodegradation and Metabolism - Metabolism of Terpenoids and Polyketides - Amino Acid Metabolism	9	1
ko00643_Styrene degradation						
	K01426	E3.5.1.4, amiE	amidase [EC:3.5.1.4]	Metabolism of Other Amino Acids - Xenobiotics Biodegradation and Metabolism - Amino Acid Metabolism	3	1
	K01501	E3.5.5.1	nitrilase [EC:3.5.5.1]	Metabolism of Other Amino Acids - Xenobiotics Biodegradation and Metabolism - Amino Acid Metabolism - Energy Metabolism	1	1
ko00650_Butanoate metabolism						
	K00023	E1.1.1.36, phbB	acetoacetyl-CoA reductase [EC:1.1.1.36]	Carbohydrate Metabolism	3	2
	K00100	E1.1.1.-		Carbohydrate Metabolism - Xenobiotics Biodegradation and Metabolism - Lipid Metabolism	1	1
	K00135	E1.2.1.16, gabD	succinate-semialdehyde dehydrogenase (NADP+) [EC:1.2.1.16]	Carbohydrate Metabolism - Amino Acid Metabolism	2	1
	K00161	PDHA, pdhA	pyruvate dehydrogenase E1 component subunit alpha [EC:1.2.4.1]	Carbohydrate Metabolism - Amino Acid Metabolism	57	3
	K00162	PDHB, pdhB	pyruvate dehydrogenase E1 component subunit beta [EC:1.2.4.1]	Carbohydrate Metabolism - Amino Acid Metabolism	37	3
	K00239	sdhA	succinate dehydrogenase flavoprotein subunit [EC:1.3.99.1]	Carbohydrate Metabolism - Xenobiotics Biodegradation and Metabolism - Energy Metabolism	2	2
	K00240	sdhB	succinate dehydrogenase iron-sulfur protein [EC:1.3.99.1]	Carbohydrate Metabolism - Xenobiotics Biodegradation and Metabolism - Energy Metabolism	18	1
	K00626	E2.3.1.9, atoB	acetyl-CoA C-acetyltransferase [EC:2.3.1.9]	Carbohydrate Metabolism - Signal Transduction - Xenobiotics Biodegradation and Metabolism - Metabolism of Terpenoids and Polyketides - Amino Acid Metabolism - Lipid Metabolism - Energy Metabolism	4	3
	K00823	puuE	4-aminobutyrate aminotransferase [EC:2.6.1.19]	Carbohydrate Metabolism - Metabolism of Other Amino Acids - Amino Acid Metabolism	1	1
	K01640	E4.1.3.4, HMGCL, hmgL	hydroxymethylglutaryl-CoA lyase [EC:4.1.3.4]	Carbohydrate Metabolism - Transport and Catabolism - Metabolism of Terpenoids and Polyketides - Amino Acid Metabolism - Lipid Metabolism	18	1
	K01652	E2.2.1.6L, ilvB, ilvG, ilvI	acetolactate synthase I/II/III large subunit [EC:2.2.1.6]	Carbohydrate Metabolism - Metabolism of Cofactors and Vitamins - Amino Acid Metabolism	300	5
	K01692	E4.2.1.17, paaG	enoyl-CoA hydratase [EC:4.2.1.17]	Carbohydrate Metabolism - Metabolism of Other Amino Acids - Xenobiotics Biodegradation and Metabolism - Metabolism of Terpenoids and Polyketides - Amino Acid Metabolism - Lipid Metabolism	33	3
	K01726	E4.2.1.-		Carbohydrate Metabolism - Xenobiotics Biodegradation and Metabolism - Metabolism of Terpenoids and Polyketides	1	1
	K01825	fadB	3-hydroxyacyl-CoA dehydrogenase / enoyl-CoA hydratase / 3-hydroxybutyryl-CoA epimerase / enoyl-CoA isomerase [EC:1.1.1.35 4.2.1.17 5.1.2.3 5.3.3.8]	Carbohydrate Metabolism - Metabolism of Other Amino Acids - Xenobiotics Biodegradation and Metabolism - Metabolism of Terpenoids and Polyketides - Amino Acid Metabolism - Lipid Metabolism - Energy Metabolism	1	1
	K03821	phbC, phaC	polyhydroxyalkanoate synthase [EC:2.3.1.-]	Carbohydrate Metabolism	1	1
	K04073	mhpF	acetaldehyde dehydrogenase [EC:1.2.1.10]	Carbohydrate Metabolism - Xenobiotics Biodegradation and Metabolism	43	2
	K07250	gabT	4-aminobutyrate aminotransferase / (S)-3-amino-2-methylpropionate transaminase [EC:2.6.1.19 2.6.1.22]	Carbohydrate Metabolism - Metabolism of Other Amino Acids - Amino Acid Metabolism	1	1
ko00660_C5-Branched dibasic acid metabolism						
	K01652	E2.2.1.6L, ilvB, ilvG, ilvI	acetolactate synthase I/II/III large subunit [EC:2.2.1.6]	Carbohydrate Metabolism - Metabolism of Cofactors and Vitamins - Amino Acid Metabolism	300	5
	K01703	leuC	3-isopropylmalate/(R)-2-methylmalate dehydratase large subunit [EC:4.2.1.33 4.2.1.35]	Carbohydrate Metabolism - Amino Acid Metabolism	2	2
	K01704	leuD	3-isopropylmalate/(R)-2-methylmalate dehydratase small subunit [EC:4.2.1.33 4.2.1.35]	Carbohydrate Metabolism - Amino Acid Metabolism	1	1
ko00670_One carbon pool by folate						
	K00287	folA	dihydrofolate reductase [EC:1.5.1.3]	Metabolism of Cofactors and Vitamins	4	3
	K00297	E1.5.1.20, metF	methylenetetrahydrofolate reductase (NADPH) [EC:1.5.1.20]	Metabolism of Cofactors and Vitamins - Energy Metabolism	1	1
	K00548	E2.1.1.13, metH	5-methyltetrahydrofolate--homocysteine methyltransferase [EC:2.1.1.13]	Metabolism of Other Amino Acids - Metabolism of Cofactors and Vitamins - Amino Acid Metabolism	23	5
	K00560	E2.1.1.45, thyA	thymidylate synthase [EC:2.1.1.45]	Metabolism of Cofactors and Vitamins - Nucleotide Metabolism	528	9
	K00600	E2.1.2.1, glyA	glycine hydroxymethyltransferase [EC:2.1.2.1]	Metabolism of Other Amino Acids - Metabolism of Cofactors and Vitamins - Amino Acid Metabolism - Energy Metabolism	25	4
	K00604	MTFMT, fmt	methionyl-tRNA formyltransferase [EC:2.1.2.9]	Translation - Metabolism of Cofactors and Vitamins	50	5
	K00605	E2.1.2.10, gcvT	aminomethyltransferase [EC:2.1.2.10]	Metabolism of Cofactors and Vitamins - Amino Acid Metabolism - Energy Metabolism	2	2
	K01433	purU	formyltetrahydrofolate deformylase [EC:3.5.1.10]	Carbohydrate Metabolism - Metabolism of Cofactors and Vitamins	1	1
	K01938	fhs	formate--tetrahydrofolate ligase [EC:6.3.4.3]	Metabolism of Cofactors and Vitamins - Energy Metabolism	1	1

K03465	E2.1.1.148, thyX, thy1	thymidylate synthase (FAD) [EC:2.1.1.148]	Metabolism of Cofactors and Vitamins - Nucleotide Metabolism	1150	7
K11175	purN	phosphoribosylglycinamide formyltransferase 1 [EC:2.1.2.2]	Metabolism of Cofactors and Vitamins - Nucleotide Metabolism	12	2
ko00680_Methane metabolism					
K00024	mdh	malate dehydrogenase [EC:1.1.1.37]	Carbohydrate Metabolism - Energy Metabolism	3	3
K00058	serA, PHGDH	D-3-phosphoglycerate dehydrogenase [EC:1.1.1.95]	Amino Acid Metabolism - Energy Metabolism	2	1
K00121	frmA, ADH5, adhC	S-(hydroxymethyl)glutathione dehydrogenase / alcohol dehydrogenase [EC:1.1.1.284 1.1.1.1]	Carbohydrate Metabolism - Metabolism of Cofactors and Vitamins - Xenobiotics Biodegradation and Metabolism - Amino Acid Metabolism - Lipid Metabolism - Energy Metabolism	8	3
K00123	E1.2.1.2A	formate dehydrogenase, alpha subunit [EC:1.2.1.2]	Carbohydrate Metabolism - Energy Metabolism	2	2
K00201	E1.2.99.5B, fwdB, fmdB	formylmethanofuran dehydrogenase subunit B [EC:1.2.99.5]	Energy Metabolism	1	1
K00297	E1.5.1.20, metF	methylenetetrahydrofolate reductase (NADPH) [EC:1.5.1.20]	Metabolism of Cofactors and Vitamins - Energy Metabolism	1	1
K00600	E2.1.2.1, glyA	glycine hydroxymethyltransferase [EC:2.1.2.1]	Metabolism of Other Amino Acids - Metabolism of Cofactors and Vitamins - Amino Acid Metabolism - Energy Metabolism	25	4
K00831	serC, PSAT1	phosphoserine aminotransferase [EC:2.6.1.52]	Metabolism of Cofactors and Vitamins - Amino Acid Metabolism - Energy Metabolism	2	1
K01007	pps, ppsA	pyruvate, water dikinase [EC:2.7.9.2]	Carbohydrate Metabolism - Energy Metabolism	107	4
K01079	serB, PSPH	phosphoserine phosphatase [EC:3.1.3.3]	Amino Acid Metabolism - Energy Metabolism	4	3
K01595	ppc	phosphoenolpyruvate carboxylase [EC:4.1.1.31]	Carbohydrate Metabolism - Energy Metabolism	1	1
K01624	FBA, fbaA	fructose-bisphosphate aldolase, class II [EC:4.1.2.13]	Carbohydrate Metabolism - Energy Metabolism	2	1
K01895	ACSS, acs	acetyl-CoA synthetase [EC:6.2.1.1]	Carbohydrate Metabolism - Lipid Metabolism - Energy Metabolism	1	1
K02118	ATPVB, ntpB	V-type H+-transporting ATPase subunit B [EC:3.6.3.14]	Energy Metabolism	1	1
K02203	thrH	phosphoserine / homoserine phosphotransferase [EC:3.1.3.3 2.7.1.39]	Amino Acid Metabolism - Energy Metabolism	1	1
K03781	katE, CAT	catalase [EC:1.11.1.6]	Neurodegenerative Diseases - Transport and Catabolism - Amino Acid Metabolism - Energy Metabolism	1	1
K03782	katG	catalase/peroxidase [EC:1.11.1.6 1.11.1.7]	Biosynthesis of Other Secondary Metabolites - Amino Acid Metabolism - Energy Metabolism	9	1
K14940	cofF	gamma-F420-2:alpha-L-glutamate ligase [EC:6.3.2.32]	Energy Metabolism	1	1
ko00710_Carbon fixation in photosynthetic organisms					
K00024	mdh	malate dehydrogenase [EC:1.1.1.37]	Carbohydrate Metabolism - Energy Metabolism	3	3
K00029	E1.1.1.40, maeB	malate dehydrogenase (oxaloacetate-decarboxylating)(NADP+) [EC:1.1.1.40]	Carbohydrate Metabolism - Energy Metabolism	2	1
K00615	E2.2.1.1, tktA, tktB	transketolase [EC:2.2.1.1]	Carbohydrate Metabolism - Metabolism of Terpenoids and Polyketides - Energy Metabolism	59	3
K00812	E2.6.1.1A, aspB	aspartate aminotransferase [EC:2.6.1.1]	Biosynthesis of Other Secondary Metabolites - Amino Acid Metabolism - Energy Metabolism	46	3
K00927	PGK, pgk	phosphoglycerate kinase [EC:2.7.2.3]	Carbohydrate Metabolism - Energy Metabolism	2	1
K01006	ppdK	pyruvate,orthophosphate dikinase [EC:2.7.9.1]	Carbohydrate Metabolism - Energy Metabolism	1	1
K01595	ppc	phosphoenolpyruvate carboxylase [EC:4.1.1.31]	Carbohydrate Metabolism - Energy Metabolism	1	1
K01601	rbcL	ribulose-bisphosphate carboxylase large chain [EC:4.1.1.39]	Carbohydrate Metabolism - Energy Metabolism	6	3
K01610	E4.1.1.49, pckA	phosphoenolpyruvate carboxykinase (ATP) [EC:4.1.1.49]	Carbohydrate Metabolism - Energy Metabolism	4	2
K01623	ALDO, fbaB	fructose-bisphosphate aldolase, class I [EC:4.1.2.13]	Carbohydrate Metabolism - Energy Metabolism	12	1
K01624	FBA, fbaA	fructose-bisphosphate aldolase, class II [EC:4.1.2.13]	Carbohydrate Metabolism - Energy Metabolism	2	1
K01783	rpe, RPE	ribulose-phosphate 3-epimerase [EC:5.1.3.1]	Carbohydrate Metabolism - Energy Metabolism	1	1
K01808	E5.3.1.6B, rpiB	ribose 5-phosphate isomerase B [EC:5.3.1.6]	Carbohydrate Metabolism - Energy Metabolism	10	3
K04041	fbp3	fructose-1,6-bisphosphatase III [EC:3.1.3.11]	Carbohydrate Metabolism - Energy Metabolism	1	1
ko00720_Carbon fixation pathways in prokaryotes					
K00024	mdh	malate dehydrogenase [EC:1.1.1.37]	Carbohydrate Metabolism - Energy Metabolism	3	3
K00174	korA	2-oxoglutarate ferredoxin oxidoreductase subunit alpha [EC:1.2.7.3]	Carbohydrate Metabolism - Energy Metabolism	1	1
K00175	korB	2-oxoglutarate ferredoxin oxidoreductase subunit beta [EC:1.2.7.3]	Carbohydrate Metabolism - Energy Metabolism	2	2
K00239	sdhA	succinate dehydrogenase flavoprotein subunit [EC:1.3.99.1]	Carbohydrate Metabolism - Xenobiotics Biodegradation and Metabolism - Energy Metabolism	2	2
K00240	sdhB	succinate dehydrogenase iron-sulfur protein [EC:1.3.99.1]	Carbohydrate Metabolism - Xenobiotics Biodegradation and Metabolism - Energy Metabolism	18	1
K00297	E1.5.1.20, metF	methylenetetrahydrofolate reductase (NADPH) [EC:1.5.1.20]	Metabolism of Cofactors and Vitamins - Energy Metabolism	1	1
K00626	E2.3.1.9, atoB	acetyl-CoA C-acetyltransferase [EC:2.3.1.9]	Carbohydrate Metabolism - Signal Transduction - Xenobiotics Biodegradation and Metabolism - Metabolism of Terpenoids and Polyketides - Amino Acid Metabolism - Lipid Metabolism - Energy Metabolism	4	3
K01007	pps, ppsA	pyruvate, water dikinase [EC:2.7.9.2]	Carbohydrate Metabolism - Energy Metabolism	107	4
K01595	ppc	phosphoenolpyruvate carboxylase [EC:4.1.1.31]	Carbohydrate Metabolism - Energy Metabolism	1	1
K01681	ACO, acnA	aconitate hydratase 1 [EC:4.2.1.3]	Carbohydrate Metabolism - Energy Metabolism	4	3
K01825	fadB	3-hydroxyacyl-CoA dehydrogenase / enoyl-CoA hydratase / 3-hydroxybutyryl-CoA epimerase / enoyl-CoA isomerase [EC:1.1.1.35 4.2.1.17 5.1.2.3 5.3.3.8]	Carbohydrate Metabolism - Metabolism of Other Amino Acids - Xenobiotics Biodegradation and Metabolism - Metabolism of Terpenoids and Polyketides - Amino Acid Metabolism - Lipid Metabolism - Energy Metabolism	1	1
K01847	MUT	methylmalonyl-CoA mutase [EC:5.4.99.2]	Carbohydrate Metabolism - Amino Acid Metabolism - Energy Metabolism	2	2
K01848	E5.4.99.2A, mcmA1	methylmalonyl-CoA mutase, N-terminal domain [EC:5.4.99.2]	Carbohydrate Metabolism - Amino Acid Metabolism - Energy Metabolism	1	1
K01895	ACSS, acs	acetyl-CoA synthetase [EC:6.2.1.1]	Carbohydrate Metabolism - Lipid Metabolism - Energy Metabolism	1	1
K01938	fhs	formate--tetrahydrofolate ligase [EC:6.3.4.3]	Metabolism of Cofactors and Vitamins - Energy Metabolism	1	1
K01962	accA	acetyl-CoA carboxylase carboxyl transferase subunit alpha [EC:6.4.1.2]	Carbohydrate Metabolism - Metabolism of Terpenoids and Polyketides - Lipid Metabolism - Energy Metabolism	1	1
K01963	accD	acetyl-CoA carboxylase carboxyl transferase subunit beta [EC:6.4.1.2]	Carbohydrate Metabolism - Metabolism of Terpenoids and Polyketides - Lipid Metabolism - Energy Metabolism	2	2
ko00730_Thiamine metabolism					
K00788	thiE	thiamine-phosphate pyrophosphorylase [EC:2.5.1.3]	Metabolism of Cofactors and Vitamins	88	1
K03707	tenA	thiaminase (transcriptional activator TenA) [EC:3.5.99.2]	Transcription - Metabolism of Cofactors and Vitamins	2	1
K04487	iscS, NFS1	cysteine desulfurase [EC:2.8.1.7]	Folding, Sorting and Degradation - Metabolism of Cofactors and Vitamins	4	3
K11717	sufS	cysteine desulfurase / selenocysteine lyase [EC:2.8.1.7 4.4.1.16]	Metabolism of Other Amino Acids - Metabolism of Cofactors and Vitamins	1	1
ko00740_Riboflavin metabolism					
K01497	ribA, RIB1	GTP cyclohydrolase II [EC:3.5.4.25]	Metabolism of Cofactors and Vitamins	1	1
K09474	phoN	acid phosphatase (class A) [EC:3.1.3.2]	Signal Transduction - Metabolism of Cofactors and Vitamins - Xenobiotics Biodegradation and Metabolism	8	2
K11753	ribF	riboflavin kinase / FMN adenylyltransferase [EC:2.7.1.26 2.7.7.2]	Metabolism of Cofactors and Vitamins	3	2
K14652	ribBA	3,4-dihydroxy 2-butanone 4-phosphate synthase / GTP cyclohydrolase II [EC:4.1.99.12 3.5.4.25]	Metabolism of Cofactors and Vitamins	4	2
K14656	ribL	FAD synthetase	Metabolism of Cofactors and Vitamins	27	3
ko00750_Vitamin B6 metabolism					
K00275	pdxH, PNPO	pyridoxamine 5'-phosphate oxidase [EC:1.4.3.5]	Metabolism of Cofactors and Vitamins	1	1
K00831	serC, PSAT1	phosphoserine aminotransferase [EC:2.6.1.52]	Metabolism of Cofactors and Vitamins - Amino Acid Metabolism - Energy Metabolism	2	1
ko00760_Nicotinate and nicotinamide metabolism					
K00278	nadB	L-aspartate oxidase [EC:1.4.3.16]	Metabolism of Cofactors and Vitamins - Amino Acid Metabolism	1	1
K00325	pntB	NAD(P) transhydrogenase subunit beta [EC:1.6.1.2]	Metabolism of Cofactors and Vitamins	1	1
K00763	E2.4.2.11, pncB	nicotinate phosphoribosyltransferase [EC:2.4.2.11]	Metabolism of Cofactors and Vitamins	11	3
K00969	nadD	nicotinate-nucleotide adenylyltransferase [EC:2.7.7.18]	Metabolism of Cofactors and Vitamins	5	2
K01081	E3.1.3.5	5'-nucleotidase [EC:3.1.3.5]	Signaling Molecules and Interaction - Metabolism of Cofactors and Vitamins - Nucleotide Metabolism	3	3
K01916	nadE	NAD+ synthase [EC:6.3.1.5]	Metabolism of Cofactors and Vitamins - Energy Metabolism	80	5
K03462	E2.4.2.12, PBEF1	nicotinamide phosphoribosyltransferase [EC:2.4.2.12]	Metabolism of Cofactors and Vitamins	2	2
K03517	nadA	quinolinate synthase [EC:2.5.1.72]	Metabolism of Cofactors and Vitamins	3	3
K03783	punA	purine-nucleoside phosphorylase [EC:2.4.2.1]	Metabolism of Cofactors and Vitamins - Nucleotide Metabolism	1	1
K03784	deoD	purine-nucleoside phosphorylase [EC:2.4.2.1]	Metabolism of Cofactors and Vitamins - Nucleotide Metabolism	2	2
K08281	pncA	nicotinamidase/pyrazinamidase [EC:3.5.1.19 3.5.1.-]	Metabolism of Cofactors and Vitamins	1	1
K13522	K13522, nadM	bifunctional NMN adenylyltransferase/nudix hydrolase [EC:2.7.7.1 3.6.1.-]	Metabolism of Cofactors and Vitamins	9	5
ko00770_Pantothenate and CoA biosynthesis					
K00053	ilvC	ketol-acid reductoisomerase [EC:1.1.1.86]	Metabolism of Cofactors and Vitamins - Amino Acid Metabolism	2	2
K00606	panB	3-methyl-2-oxobutanoate hydroxymethyltransferase [EC:2.1.2.11]	Metabolism of Cofactors and Vitamins	26	4
K00826	E2.6.1.42, ilvE	branched-chain amino acid aminotransferase [EC:2.6.1.42]	Metabolism of Cofactors and Vitamins - Amino Acid Metabolism	3	2
K00954	E2.7.7.3A, coaD, kdtB	pantetheine-phosphate adenylyltransferase [EC:2.7.7.3]	Metabolism of Cofactors and Vitamins	9	2
K01652	E2.2.1.6L, ilvB, ilvG, ilvI	acetolactate synthase I/II/III large subunit [EC:2.2.1.6]	Carbohydrate Metabolism - Metabolism of Cofactors and Vitamins - Amino Acid Metabolism	300	5
K02201	E2.7.7.3B	pantetheine-phosphate adenylyltransferase [EC:2.7.7.3]	Metabolism of Cofactors and Vitamins	4	2

ko00780_Biotin metabolism					
K00652	bioF	8-amino-7-oxononanoate synthase [EC:2.3.1.47]	Metabolism of Cofactors and Vitamins - Amino Acid Metabolism	1	1
K01423	E3.4.-.-		Metabolism of Cofactors and Vitamins - Amino Acid Metabolism	7	4
ko00790_Folate biosynthesis					
K00287	folA	dihydrofolate reductase [EC:1.5.1.3]	Metabolism of Cofactors and Vitamins	4	3
K00796	folP	dihydropteroate synthase [EC:2.5.1.15]	Metabolism of Cofactors and Vitamins	1	1
K01495	E3.5.4.16, folE	GTP cyclohydrolase I [EC:3.5.4.16]	Metabolism of Cofactors and Vitamins	495	8
K01737	E4.2.3.12, ptpS	6-pyruvoyl tetrahydrobiopterin synthase [EC:4.2.3.12]	Metabolism of Cofactors and Vitamins	127	5
K03342	pabBC	para-aminobenzoate synthetase / 4-amino-4-deoxychorismate lyase [EC:2.6.1.85 4.1.3.38]	Metabolism of Cofactors and Vitamins - Amino Acid Metabolism	1	1
K03639	MOCS1, moaA	molybdenum cofactor biosynthesis protein	Folding, Sorting and Degradation - Metabolism of Cofactors and Vitamins	1	1
K04071	E1.1.1.220	6-pyruvoyltetrahydropterin 2'-reductase [EC:1.1.1.220]	Metabolism of Cofactors and Vitamins	4	1
ko00791_Atrazine degradation					
K01428	ureC	urease subunit alpha [EC:3.5.1.5]	Infectious Diseases - Nucleotide Metabolism - Xenobiotics Biodegradation and Metabolism - Amino Acid Metabolism	2	1
K01429	ureB	urease subunit beta [EC:3.5.1.5]	Nucleotide Metabolism - Xenobiotics Biodegradation and Metabolism - Amino Acid Metabolism	1	1
ko00830_Retinol metabolism					
K00001	E1.1.1.1, adh	alcohol dehydrogenase [EC:1.1.1.1]	Carbohydrate Metabolism - Metabolism of Cofactors and Vitamins - Xenobiotics Biodegradation and Metabolism - Amino Acid Metabolism - Lipid Metabolism	1	1
K00121	frmA, ADH5, adhC	S-(hydroxymethyl)glutathione dehydrogenase / alcohol dehydrogenase [EC:1.1.1.284 1.1.1.1]	Carbohydrate Metabolism - Metabolism of Cofactors and Vitamins - Xenobiotics Biodegradation and Metabolism - Amino Acid Metabolism - Lipid Metabolism - Energy Metabolism	8	3
K13953	adhP	alcohol dehydrogenase, propanol-preferring [EC:1.1.1.1]	Carbohydrate Metabolism - Metabolism of Cofactors and Vitamins - Xenobiotics Biodegradation and Metabolism - Amino Acid Metabolism - Lipid Metabolism	1	1
ko00860_Porphyrin and chlorophyll metabolism					
K00228	hemF, CPOX	coproporphyrinogen III oxidase [EC:1.3.3.3]	Metabolism of Cofactors and Vitamins	4	2
K00510	HMOX, hmuO, ho	heme oxygenase [EC:1.14.99.3]	Digestive System - Metabolism of Cofactors and Vitamins	2	1
K00643	E2.3.1.37, ALAS	5-aminolevulinate synthase [EC:2.3.1.37]	Metabolism of Cofactors and Vitamins - Amino Acid Metabolism	7	4
K00768	E2.4.2.21, cobU, cobT	nicotinate-nucleotide--dimethylbenzimidazole phosphoribosyltransferase [EC:2.4.2.21]	Metabolism of Cofactors and Vitamins	1	1
K00798	E2.5.1.17, cobO, btuR	cob(I)alamin adenosyltransferase [EC:2.5.1.17]	Metabolism of Cofactors and Vitamins	2	1
K01599	hemE, UROD	uroporphyrinogen decarboxylase [EC:4.1.1.37]	Metabolism of Cofactors and Vitamins	2	1
K01749	hemC, HMBS	hydroxymethylbilane synthase [EC:2.5.1.61]	Metabolism of Cofactors and Vitamins	3	1
K01772	hemH, FECH	ferrochelatase [EC:4.99.1.1]	Metabolism of Cofactors and Vitamins	1	1
K01845	hemL	glutamate-1-semialdehyde 2,1-aminomutase [EC:5.4.3.8]	Metabolism of Cofactors and Vitamins - Amino Acid Metabolism	7	4
K01885	EARS, gltX	glutamyl-tRNA synthetase [EC:6.1.1.17]	Translation - Metabolism of Cofactors and Vitamins - Amino Acid Metabolism	1	1
K02217	ftnA, ftn	ferritin [EC:1.16.3.1]	Metabolism of Cofactors and Vitamins	36	2
K02230	cobN	cobaltochelatase CobN [EC:6.6.1.2]	Metabolism of Cofactors and Vitamins	1	1
K02232	E6.3.5.10, cobQ, cbiP	adenosylcobyric acid synthase [EC:6.3.5.10]	Metabolism of Cofactors and Vitamins	1	1
K02301	cyoE	protoheme IX farnesyltransferase [EC:2.5.1.-]	Metabolism of Cofactors and Vitamins - Metabolism of Terpenoids and Polyketides - Energy Metabolism	2	2
K02495	hemN, hemZ	oxygen-independent coproporphyrinogen III oxidase [EC:1.3.99.22]	Metabolism of Cofactors and Vitamins	3	3
K03404	chlD, bchD	magnesium chelatase subunit D [EC:6.6.1.1]	Metabolism of Cofactors and Vitamins	1	1
K03795	cbiX	sirohydrochlorin cobaltochelatase [EC:4.99.1.3]	Metabolism of Cofactors and Vitamins	1	1
K04034	bchE	anaerobic magnesium-protoporphyrin IX monomethyl ester cyclase [EC:4.-.-.-]	Metabolism of Cofactors and Vitamins	1	1
K05371	pcyA	phycocyanobilin:ferredoxin oxidoreductase [EC:1.3.7.5]	Metabolism of Cofactors and Vitamins	4	2
K09882	cobS	cobaltochelatase CobS [EC:6.6.1.2]	Metabolism of Cofactors and Vitamins	39	2
ko00900_Terpenoid backbone biosynthesis					
K00054	E1.1.1.88	hydroxymethylglutaryl-CoA reductase [EC:1.1.1.88]	Metabolism of Terpenoids and Polyketides	1	1
K00099	dxr	1-deoxy-D-xylulose-5-phosphate reductoisomerase [EC:1.1.1.267]	Metabolism of Terpenoids and Polyketides	1	1
K00626	E2.3.1.9, atoB	acetyl-CoA C-acetyltransferase [EC:2.3.1.9]	Carbohydrate Metabolism - Signal Transduction - Xenobiotics Biodegradation and Metabolism - Metabolism of Terpenoids and Polyketides - Amino Acid Metabolism - Lipid Metabolism - Energy Metabolism	4	3
K00795	ispA	farnesyl diphosphate synthase [EC:2.5.1.1 2.5.1.10]	Metabolism of Terpenoids and Polyketides	1	1
K00806	uppS	undecaprenyl diphosphate synthase [EC:2.5.1.31]	Metabolism of Terpenoids and Polyketides	6	1
K00869	E2.7.1.36, MVK, mvaK1	mevalonate kinase [EC:2.7.1.36]	Transport and Catabolism - Metabolism of Terpenoids and Polyketides	1	1
K00938	E2.7.4.2, mvaK2	phosphomevalonate kinase [EC:2.7.4.2]	Metabolism of Terpenoids and Polyketides	1	1
K01662	dxs	1-deoxy-D-xylulose-5-phosphate synthase [EC:2.2.1.7]	Metabolism of Terpenoids and Polyketides	2	2
K01770	ispF	2-C-methyl-D-erythritol 2,4-cyclodiphosphate synthase [EC:4.6.1.12]	Metabolism of Terpenoids and Polyketides	3	1
K02523	ispB	octaprenyl-diphosphate synthase [EC:2.5.1.90]	Metabolism of Terpenoids and Polyketides	1	1
K03526	E1.17.7.1, gcpE, ispG	(E)-4-hydroxy-3-methylbut-2-enyl-diphosphate synthase [EC:1.17.7.1]	Metabolism of Terpenoids and Polyketides	4	3
K03527	E1.17.1.2, lytB, ispH	4-hydroxy-3-methylbut-2-enyl diphosphate reductase [EC:1.17.1.2]	Metabolism of Terpenoids and Polyketides	2	2
ko00903_Limonene and pinene degradation					
K00128	E1.2.1.3	aldehyde dehydrogenase (NAD+) [EC:1.2.1.3]	Carbohydrate Metabolism - Metabolism of Other Amino Acids - Xenobiotics Biodegradation and Metabolism - Metabolism of Terpenoids and Polyketides - Amino Acid Metabolism - Lipid Metabolism	4	4
K00680	E2.3.1.-		Xenobiotics Biodegradation and Metabolism - Metabolism of Terpenoids and Polyketides - Amino Acid Metabolism	9	1
K01076	E3.1.2.-		Metabolism of Terpenoids and Polyketides - Lipid Metabolism	1	1
K01692	E4.2.1.17, paaG	enoyl-CoA hydratase [EC:4.2.1.17]	Carbohydrate Metabolism - Metabolism of Other Amino Acids - Xenobiotics Biodegradation and Metabolism - Metabolism of Terpenoids and Polyketides - Amino Acid Metabolism - Lipid Metabolism	33	3
K01726	E4.2.1.-		Carbohydrate Metabolism - Xenobiotics Biodegradation and Metabolism - Metabolism of Terpenoids and Polyketides	1	1
K01825	fadB	3-hydroxyacyl-CoA dehydrogenase / enoyl-CoA hydratase / 3-hydroxybutyryl-CoA epimerase / enoyl-CoA isomerase [EC:1.1.1.35 4.2.1.17 5.1.2.3 5.3.3.8]	Carbohydrate Metabolism - Metabolism of Other Amino Acids - Xenobiotics Biodegradation and Metabolism - Metabolism of Terpenoids and Polyketides - Amino Acid Metabolism - Lipid Metabolism - Energy Metabolism	1	1
ko00906_Carotenoid biosynthesis					
K02291	crtB	phytoene synthase [EC:2.5.1.32]	Metabolism of Terpenoids and Polyketides	1	1
K10027	crtI	phytoene dehydrogenase [EC:1.14.99.-]	Metabolism of Terpenoids and Polyketides	4	2
ko00910_Nitrogen metabolism					
K00261	E1.4.1.3	glutamate dehydrogenase (NAD(P)+) [EC:1.4.1.3]	Metabolism of Other Amino Acids - Excretory System - Amino Acid Metabolism - Energy Metabolism	3	2
K00265	gltB	glutamate synthase (NADPH/NADH) large chain [EC:1.4.1.13 1.4.1.14]	Amino Acid Metabolism - Energy Metabolism	3	3
K00266	gltD	glutamate synthase (NADPH/NADH) small chain [EC:1.4.1.13 1.4.1.14]	Amino Acid Metabolism - Energy Metabolism	1	1
K00285	dadA	D-amino-acid dehydrogenase [EC:1.4.99.1]	Amino Acid Metabolism - Energy Metabolism	19	4
K00362	E1.7.1.4L, nirB	nitrite reductase (NAD(P)H) large subunit [EC:1.7.1.4]	Energy Metabolism	1	1
K00368	E1.7.2.1	nitrite reductase (NO-forming) [EC:1.7.2.1]	Energy Metabolism	1	1
K00370	narG	nitrate reductase 1, alpha subunit [EC:1.7.99.4]	Signal Transduction - Energy Metabolism	1	1
K00372	E1.7.99.4C	nitrate reductase catalytic subunit [EC:1.7.99.4]	Energy Metabolism	1	1
K00459	E1.13.12.16	nitronate monooxygenase [EC:1.13.12.16]	Energy Metabolism	1	1
K00605	E2.1.2.10, gcvT	aminomethyltransferase [EC:2.1.2.10]	Metabolism of Cofactors and Vitamins - Amino Acid Metabolism - Energy Metabolism	2	2
K01424	E3.5.1.1, ansA, ansB	L-asparaginase [EC:3.5.1.1]	Metabolism of Other Amino Acids - Amino Acid Metabolism - Energy Metabolism	1	1
K01501	E3.5.5.1	nitrilase [EC:3.5.5.1]	Metabolism of Other Amino Acids - Xenobiotics Biodegradation and Metabolism - Amino Acid Metabolism - Energy Metabolism	1	1
K01667	E4.1.99.1, tnaA	tryptophanase [EC:4.1.99.1]	Amino Acid Metabolism - Energy Metabolism	1	1
K01673	cynT, can	carbonic anhydrase [EC:4.2.1.1]	Energy Metabolism	2	2
K01760	metC	cystathionine beta-lyase [EC:4.4.1.8]	Metabolism of Other Amino Acids - Amino Acid Metabolism - Energy Metabolism	2	1
K01915	E6.3.1.2, glnA	glutamine synthetase [EC:6.3.1.2]	Signal Transduction - Nervous System - Amino Acid Metabolism - Energy Metabolism	113	7
K01916	nadE	NAD+ synthase [EC:6.3.1.5]	Metabolism of Cofactors and Vitamins - Energy Metabolism	80	5
K01953	E6.3.5.4, asnB	asparagine synthase (glutamine-hydrolysing) [EC:6.3.5.4]	Enzyme Families - Amino Acid Metabolism - Energy Metabolism	14	2
K02592	nifN	nitrogenase molybdenum-iron protein NifN	Energy Metabolism	1	1
K04748	norQ	nitric-oxide reductase NorQ protein [EC:1.7.99.7]	Energy Metabolism	15	2

ko00920_Sulfur metabolism					
K00640	E2.3.1.30, cysE	serine O-acetyltransferase [EC:2.3.1.30]	Amino Acid Metabolism - Energy Metabolism	2	2
K00641	E2.3.1.31, metX	homoserine O-acetyltransferase [EC:2.3.1.31]	Amino Acid Metabolism - Energy Metabolism	1	1
K00860	cysC	adenylylsulfate kinase [EC:2.7.1.25]	Nucleotide Metabolism - Energy Metabolism	165	4
K00955	cysNC	bifunctional enzyme CysN/CysC [EC:2.7.7.4 2.7.1.25]	Metabolism of Other Amino Acids - Nucleotide Metabolism - Energy Metabolism	2	2
K00957	cysD	sulfate adenylyltransferase subunit 2 [EC:2.7.7.4]	Metabolism of Other Amino Acids - Nucleotide Metabolism - Energy Metabolism	2	2
K01738	cysK	cysteine synthase A [EC:2.5.1.47]	Amino Acid Metabolism - Energy Metabolism	30	5
K01760	metC	cystathionine beta-lyase [EC:4.4.1.8]	Metabolism of Other Amino Acids - Amino Acid Metabolism - Energy Metabolism	2	1
K12339	cysM	cysteine synthase B [EC:2.5.1.47]	Amino Acid Metabolism - Energy Metabolism	5	2
ko00930_Caprolactam degradation					
K01692	E4.2.1.17, paaG	enoyl-CoA hydratase [EC:4.2.1.17]	Carbohydrate Metabolism - Metabolism of Other Amino Acids - Xenobiotics Biodegradation and Metabolism - Metabolism of Terpenoids and Polyketides - Amino Acid Metabolism - Lipid Metabolism	33	3
K01825	fadB	3-hydroxyacyl-CoA dehydrogenase / enoyl-CoA hydratase / 3-hydroxybutyryl-CoA epimerase / enoyl-CoA isomerase [EC:1.1.1.35 4.2.1.17 5.1.2.3 5.3.3.8]	Carbohydrate Metabolism - Metabolism of Other Amino Acids - Xenobiotics Biodegradation and Metabolism - Metabolism of Terpenoids and Polyketides - Amino Acid Metabolism - Lipid Metabolism - Energy Metabolism	1	1
ko00940_Phenylpropanoid biosynthesis					
K00588	E2.1.1.104	caffeoyl-CoA O-methyltransferase [EC:2.1.1.104]	Biosynthesis of Other Secondary Metabolites - Amino Acid Metabolism	1	1
K03782	katG	catalase/peroxidase [EC:1.11.1.6 1.11.1.7]	Biosynthesis of Other Secondary Metabolites - Amino Acid Metabolism - Energy Metabolism	9	1
K05349	bglX	beta-glucosidase [EC:3.2.1.21]	Carbohydrate Metabolism - Metabolism of Other Amino Acids - Biosynthesis of Other Secondary Metabolites	1	1
K05350	bglB	beta-glucosidase [EC:3.2.1.21]	Carbohydrate Metabolism - Metabolism of Other Amino Acids - Biosynthesis of Other Secondary Metabolites	4	2
ko00941_Flavonoid biosynthesis					
K00091	E1.1.1.219	dihydroflavonol-4-reductase [EC:1.1.1.219]	Biosynthesis of Other Secondary Metabolites	1	1
K00588	E2.1.1.104	caffeoyl-CoA O-methyltransferase [EC:2.1.1.104]	Biosynthesis of Other Secondary Metabolites - Amino Acid Metabolism	1	1
ko00945_Stilbenoid, diarylheptanoid and gingerol biosynthesis					
K00588	E2.1.1.104	caffeoyl-CoA O-methyltransferase [EC:2.1.1.104]	Biosynthesis of Other Secondary Metabolites - Amino Acid Metabolism	1	1
ko00950_Isoquinoline alkaloid biosynthesis					
K00812	E2.6.1.1A, aspB	aspartate aminotransferase [EC:2.6.1.1]	Biosynthesis of Other Secondary Metabolites - Amino Acid Metabolism - Energy Metabolism	46	3
ko00960_Tropane, piperidine and pyridine alkaloid biosynthesis					
K00808	hss	homospermidine synthase [EC:2.5.1.44]	Biosynthesis of Other Secondary Metabolites	1	1
K00812	E2.6.1.1A, aspB	aspartate aminotransferase [EC:2.6.1.1]	Biosynthesis of Other Secondary Metabolites - Amino Acid Metabolism - Energy Metabolism	46	3
K00817	hisC	histidinol-phosphate aminotransferase [EC:2.6.1.9]	Biosynthesis of Other Secondary Metabolites - Amino Acid Metabolism	6	3
ko00970_Aminoacyl-tRNA biosynthesis					
K00604	MTFMT, fmt	methionyl-tRNA formyltransferase [EC:2.1.2.9]	Translation - Metabolism of Cofactors and Vitamins	50	5
K01866	YARS, tyrS	tyrosyl-tRNA synthetase [EC:6.1.1.1]	Translation - Amino Acid Metabolism	1	1
K01867	WARS, trpS	tryptophanyl-tRNA synthetase [EC:6.1.1.2]	Translation - Amino Acid Metabolism	3	1
K01869	LARS, leuS	leucyl-tRNA synthetase [EC:6.1.1.4]	Translation - Amino Acid Metabolism	2	2
K01870	IARS, ileS	isoleucyl-tRNA synthetase [EC:6.1.1.5]	Translation - Amino Acid Metabolism	3	2
K01872	AARS, alaS	alanyl-tRNA synthetase [EC:6.1.1.7]	Translation - Amino Acid Metabolism	7	4
K01873	VARS, valS	valyl-tRNA synthetase [EC:6.1.1.9]	Translation - Amino Acid Metabolism	3	2
K01874	MARS, metG	methionyl-tRNA synthetase [EC:6.1.1.10]	Translation - Metabolism of Other Amino Acids - Amino Acid Metabolism	6	3
K01875	SARS, serS	seryl-tRNA synthetase [EC:6.1.1.11]	Translation - Amino Acid Metabolism	3	2
K01876	DARS, aspS	aspartyl-tRNA synthetase [EC:6.1.1.12]	Translation - Amino Acid Metabolism	1	1
K01881	PARS, proS	prolyl-tRNA synthetase [EC:6.1.1.15]	Translation - Amino Acid Metabolism	2	1
K01883	CARS, cysS	cysteinyl-tRNA synthetase [EC:6.1.1.16]	Translation - Amino Acid Metabolism	2	2
K01885	EARS, gltX	glutamyl-tRNA synthetase [EC:6.1.1.17]	Translation - Metabolism of Cofactors and Vitamins - Amino Acid Metabolism	1	1
K01886	QARS, glnS	glutaminyl-tRNA synthetase [EC:6.1.1.18]	Translation - Amino Acid Metabolism	3	1
K01887	RARS, argS	arginyl-tRNA synthetase [EC:6.1.1.19]	Translation - Amino Acid Metabolism	2	1
K01890	FARSB, pheT	phenylalanyl-tRNA synthetase beta chain [EC:6.1.1.20]	Translation - Amino Acid Metabolism	2	1
K01892	HARS, hisS	histidyl-tRNA synthetase [EC:6.1.1.21]	Translation - Amino Acid Metabolism	1	1
K01893	NARS, asnS	asparaginyl-tRNA synthetase [EC:6.1.1.22]	Translation - Amino Acid Metabolism	1	1
K02433	gatA	aspartyl-tRNA(Asn)/glutamyl-tRNA (Gln) amidotransferase subunit A [EC:6.3.5.6 6.3.5.7]	Translation	1	1
K04567	KARS, lysS	lysyl-tRNA synthetase, class II [EC:6.1.1.6]	Translation - Amino Acid Metabolism	18	2
K09482	gatD	glutamyl-tRNA(Gln) amidotransferase subunit D [EC:6.3.5.7]	Translation	1	1
ko00980_Metabolism of xenobiotics by cytochrome P450					
K00001	E1.1.1.1, adh	alcohol dehydrogenase [EC:1.1.1.1]	Carbohydrate Metabolism - Metabolism of Cofactors and Vitamins - Xenobiotics Biodegradation and Metabolism - Amino Acid Metabolism - Lipid Metabolism	1	1
K00121	frmA, ADH5, adhC	S-(hydroxymethyl)glutathione dehydrogenase / alcohol dehydrogenase [EC:1.1.1.284 1.1.1.1]	Carbohydrate Metabolism - Metabolism of Cofactors and Vitamins - Xenobiotics Biodegradation and Metabolism - Amino Acid Metabolism - Lipid Metabolism - Energy Metabolism	8	3
K13953	adhP	alcohol dehydrogenase, propanol-preferring [EC:1.1.1.1]	Carbohydrate Metabolism - Metabolism of Cofactors and Vitamins - Xenobiotics Biodegradation and Metabolism - Amino Acid Metabolism - Lipid Metabolism	1	1
ko00982_Drug metabolism - cytochrome P450					
K00001	E1.1.1.1, adh	alcohol dehydrogenase [EC:1.1.1.1]	Carbohydrate Metabolism - Metabolism of Cofactors and Vitamins - Xenobiotics Biodegradation and Metabolism - Amino Acid Metabolism - Lipid Metabolism	1	1
K00121	frmA, ADH5, adhC	S-(hydroxymethyl)glutathione dehydrogenase / alcohol dehydrogenase [EC:1.1.1.284 1.1.1.1]	Carbohydrate Metabolism - Metabolism of Cofactors and Vitamins - Xenobiotics Biodegradation and Metabolism - Amino Acid Metabolism - Lipid Metabolism - Energy Metabolism	8	3
K13953	adhP	alcohol dehydrogenase, propanol-preferring [EC:1.1.1.1]	Carbohydrate Metabolism - Metabolism of Cofactors and Vitamins - Xenobiotics Biodegradation and Metabolism - Amino Acid Metabolism - Lipid Metabolism	1	1
ko00983_Drug metabolism - other enzymes					
K00088	E1.1.1.205, guaB	IMP dehydrogenase [EC:1.1.1.205]	Nucleotide Metabolism - Xenobiotics Biodegradation and Metabolism	57	4
K00760	E2.4.2.8, hpt	hypoxanthine phosphoribosyltransferase [EC:2.4.2.8]	Nucleotide Metabolism - Xenobiotics Biodegradation and Metabolism	62	4
K00857	E2.7.1.21, tdk	thymidine kinase [EC:2.7.1.21]	Nucleotide Metabolism - Xenobiotics Biodegradation and Metabolism	13	3
K01951	E6.3.5.2, guaA	GMP synthase (glutamine-hydrolysing) [EC:6.3.5.2]	Enzyme Families - Nucleotide Metabolism - Xenobiotics Biodegradation and Metabolism	1	1
K13421	UMPS	uridine monophosphate synthetase [EC:2.4.2.10 4.1.1.23]	Nucleotide Metabolism - Xenobiotics Biodegradation and Metabolism	1	1
ko01040_Biosynthesis of unsaturated fatty acids					
K00059	fabG	3-oxoacyl-[acyl-carrier protein] reductase [EC:1.1.1.100]	Lipid Metabolism	10	4
K00507	SCD, desC	stearoyl-CoA desaturase (delta-9 desaturase) [EC:1.14.19.1]	Endocrine System - Lipid Metabolism	13	2
K01076	E3.1.2.-		Metabolism of Terpenoids and Polyketides - Lipid Metabolism	1	1
K01825	fadB	3-hydroxyacyl-CoA dehydrogenase / enoyl-CoA hydratase / 3-hydroxybutyryl-CoA epimerase / enoyl-CoA isomerase [EC:1.1.1.35 4.2.1.17 5.1.2.3 5.3.3.8]	Carbohydrate Metabolism - Metabolism of Other Amino Acids - Xenobiotics Biodegradation and Metabolism - Metabolism of Terpenoids and Polyketides - Amino Acid Metabolism - Lipid Metabolism - Energy Metabolism	1	1
K10806	yciA	acyl-CoA thioesterase YciA [EC:3.1.2.-]	Lipid Metabolism	6	2
ko01051_Biosynthesis of ansamycins					
K00615	E2.2.1.1, tktA, tktB	transketolase [EC:2.2.1.1]	Carbohydrate Metabolism - Metabolism of Terpenoids and Polyketides - Energy Metabolism	59	3
ko01053_Biosynthesis of siderophore group nonribosomal peptides					
K04789	mbtE	mycobactin peptide synthetase MbtE	Metabolism of Terpenoids and Polyketides	2	1
K12240	pchF	pyochelin synthetase	Metabolism of Terpenoids and Polyketides	4	1
ko01055_Biosynthesis of vancomycin group antibiotics					
K01710	E4.2.1.46, rfbB, rffG	dTDP-glucose 4,6-dehydratase [EC:4.2.1.46]	Biosynthesis of Other Secondary Metabolites - Metabolism of Terpenoids and Polyketides	341	7
ko02010_ABC transporters					

K01995	livG	branched-chain amino acid transport system ATP-binding protein	Membrane Transport	1	1
K01996	livF	branched-chain amino acid transport system ATP-binding protein	Membrane Transport	3	2
K01997	livH	branched-chain amino acid transport system permease protein	Membrane Transport	1	1
K01998	livM	branched-chain amino acid transport system permease protein	Membrane Transport	5	3
K01999	livK	branched-chain amino acid transport system substrate-binding protein	Membrane Transport	2	2
K02001	proW	glycine betaine/proline transport system permease protein	Membrane Transport	9	3
K02010	afuC, fbpC	iron(III) transport system ATP-binding protein [EC:3.6.3.30]	Membrane Transport	1	1
K02011	afuB, fbpB	iron(III) transport system permease protein	Membrane Transport	2	1
K02013	ABC.FEV.A	iron complex transport system ATP-binding protein [EC:3.6.3.34]	Membrane Transport	1	1
K02032	ABC.PE.A1	peptide/nickel transport system ATP-binding protein	Membrane Transport	3	2
K02033	ABC.PE.P	peptide/nickel transport system permease protein	Membrane Transport	2	2
K02034	ABC.PE.P1	peptide/nickel transport system permease protein	Membrane Transport	3	3
K02035	ABC.PE.S	peptide/nickel transport system substrate-binding protein	Membrane Transport	8	4
K02040	pstS	phosphate transport system substrate-binding protein	Signal Transduction - Infectious Diseases - Membrane Transport	57	4
K02042	phnE	phosphonate transport system permease protein	Membrane Transport	1	1
K02046	cysU	sulfate transport system permease protein	Membrane Transport	1	1
K02049	ABC.SN.A, ssuB, tauB	sulfonate/nitrate/taurine transport system ATP-binding protein	Membrane Transport	4	3
K02050	ABC.SN.P, ssuC, tauC	sulfonate/nitrate/taurine transport system permease protein	Membrane Transport	3	2
K02072	ABC.MET.P, metI	D-methionine transport system permease protein	Membrane Transport	1	1
K02073	ABC.MET.S, metQ	D-methionine transport system substrate-binding protein	Membrane Transport	1	1
K02194	ccmB	heme exporter protein B	Membrane Transport	1	1
K02195	ccmC	heme exporter protein C	Membrane Transport	1	1
K05685	macB	macrolide transport system ATP-binding/permease protein [EC:3.6.3.-]	Membrane Transport	1	1
K05814	ugpA	sn-glycerol 3-phosphate transport system permease protein	Membrane Transport	1	1
K06857	ABC.TG.A	putative tungstate transport system ATP-binding protein	Membrane Transport	1	1
K06861	lptB	lipopolysaccharide export system ATP-binding protein [EC:3.6.3.-]	Membrane Transport	1	1
K09686	ABC-2.AB.P	antibiotic transport system permease protein	Membrane Transport	4	1
K09687	ABC-2.AB.A	antibiotic transport system ATP-binding protein	Membrane Transport	1	1
K09691	ABC-2.LPSE.A	lipopolysaccharide transport system ATP-binding protein	Membrane Transport	4	2
K09695	nodI	lipooligosaccharide transport system ATP-binding protein	Membrane Transport	1	1
K09808	ABC.LPT.P, lolC, lolE	lipoprotein-releasing system permease protein	Membrane Transport	1	1
K10005	gluB	glutamate transport system substrate-binding protein	Membrane Transport	1	1
K10107	ABC-2.CPSE.P1	capsular polysaccharide transport system permease protein	Membrane Transport	1	1
K10112	msmX, msmK	maltose/maltodextrin transport system ATP-binding protein	Membrane Transport	1	1
K11952	cmpC	bicarbonate transport system ATP-binding protein [EC:3.6.3.-]	Membrane Transport	2	1
K11962	urtD	urea transport system ATP-binding protein	Membrane Transport	1	1
ko02020_Two-component system					
K00027	E1.1.1.38, sfcA, maeA	malate dehydrogenase (oxaloacetate-decarboxylating) [EC:1.1.1.38]	Carbohydrate Metabolism - Signal Transduction	2	1
K00066	algD	GDP-mannose 6-dehydrogenase [EC:1.1.1.132]	Carbohydrate Metabolism - Signal Transduction	3	1
K00370	narG	nitrate reductase 1, alpha subunit [EC:1.7.99.4]	Signal Transduction - Energy Metabolism	1	1
K00575	cheR	chemotaxis protein methyltransferase CheR [EC:2.1.1.80]	Cell Motility - Signal Transduction	1	1
K00626	E2.3.1.9, atoB	acetyl-CoA C-acetyltransferase [EC:2.3.1.9]	Carbohydrate Metabolism - Signal Transduction - Xenobiotics Biodegradation and Metabolism - Metabolism of Terpenoids and Polyketides - Amino Acid Metabolism - Lipid Metabolism - Energy Metabolism	4	3
K00990	glnD	[protein-PII] uridylyltransferase [EC:2.7.7.59]	Signal Transduction	1	1
K01467	E3.5.2.6, ampC, penP	beta-lactamase [EC:3.5.2.6]	Signal Transduction - Biosynthesis of Other Secondary Metabolites	1	1
K01546	kdpA	K+-transporting ATPase ATPase A chain [EC:3.6.3.12]	Signal Transduction	1	1
K01915	E6.3.1.2, glnA	glutamine synthetase [EC:6.3.1.2]	Signal Transduction - Nervous System - Amino Acid Metabolism - Energy Metabolism	113	7
K02040	pstS	phosphate transport system substrate-binding protein	Signal Transduction - Infectious Diseases - Membrane Transport	57	4
K02313	dnaA	chromosomal replication initiator protein	Signal Transduction - Cell Growth and Death - Replication and Repair	470	2
K02405	fliA	RNA polymerase sigma factor for flagellar operon FliA	Cell Motility - Transcription - Signal Transduction - Infectious Diseases	7	2
K02489	pleC1	two-component system, cell cycle sensor kinase and response regulator [EC:2.7.13.3]	Signal Transduction - Enzyme Families	2	1
K02650	pilA	type IV pilus assembly protein PilA	Cell Motility - Signal Transduction - Membrane Transport	5	1
K03092	SIG54, rpoN	RNA polymerase sigma-54 factor	Transcription - Signal Transduction - Infectious Diseases	8	1
K03406	mcp	methyl-accepting chemotaxis protein	Cell Motility - Signal Transduction	74	4
K03407	cheA	two-component system, chemotaxis family, sensor kinase CheA [EC:2.7.13.3]	Cell Motility - Signal Transduction - Enzyme Families	2	2
K03413	cheY	two-component system, chemotaxis family, response regulator CheY	Cell Motility - Signal Transduction	2	2
K03563	csrA	carbon storage regulator	Signal Transduction	5	3
K04751	glnB	nitrogen regulatory protein P-II 1	Signal Transduction	1	1
K06596	chpA	chemosensory pili system protein ChpA (sensor histidine kinase/response regulator)	Cell Motility - Signal Transduction - Enzyme Families	1	1
K07636	phoR	two-component system, OmpR family, phosphate regulon sensor histidine kinase PhoR [EC:2.7.13.3]	Signal Transduction - Enzyme Families	1	1
K07639	rstB	two-component system, OmpR family, sensor histidine kinase RstB [EC:2.7.13.3]	Signal Transduction - Enzyme Families	25	1
K07645	qseC	two-component system, OmpR family, sensor histidine kinase QseC [EC:2.7.13.3]	Signal Transduction - Enzyme Families	7	2
K07649	tctE	two-component system, OmpR family, sensor histidine kinase TctE [EC:2.7.13.3]	Signal Transduction - Enzyme Families	1	1
K07665	cusR	two-component system, OmpR family, copper resistance phosphate regulon response regulator CusR	Signal Transduction	1	1
K07673	narX	two-component system, NarL family, nitrate/nitrite sensor histidine kinase NarX [EC:2.7.13.3]	Signal Transduction - Enzyme Families	1	1
K07714	atoC	two-component system, NtrC family, response regulator AtoC	Signal Transduction	2	2
K07774	tctD	two-component system, OmpR family, response regulator TctD	Signal Transduction	1	1
K07782	sdiA	LuxR family transcriptional regulator	Transcription - Signal Transduction	1	1
K07787	cusA	Cu(I)/Ag(I) efflux system membrane protein CusA	Signal Transduction	2	1
K07788	mdtB	RND superfamily, multidrug transport protein MdtB	Signal Transduction	1	1
K07794	tctB	putative tricarboxylic transport membrane protein	Signal Transduction	1	1
K07799	mdtA	putative multidrug efflux transporter MdtA	Signal Transduction	2	2
K07806	arnB, pmrH	UDP-4-amino-4-deoxy-L-arabinose-oxoglutarate aminotransferase [EC:2.6.1.87]	Carbohydrate Metabolism - Signal Transduction - Glycan Biosynthesis and Metabolism - Amino Acid Metabolism	3	2
K08359	ttrC	tetrathionate reductase subunit C	Signal Transduction	1	1
K09474	phoN	acid phosphatase (class A) [EC:3.1.3.2]	Signal Transduction - Metabolism of Cofactors and Vitamins - Xenobiotics Biodegradation and Metabolism	8	2
K10126	dctD	two-component system, NtrC family, C4-dicarboxylate transport response regulator DctD	Signal Transduction	1	1
K10682	saeR	two-component system, OmpR family, response regulator SaeR	Signal Transduction	1	1
K11331	nrsC	cation efflux system protein involved in nickel and cobalt tolerance [EC:3.2.1.17]	Signal Transduction	24	2
ko02030_Bacterial chemotaxis					
K00575	cheR	chemotaxis protein methyltransferase CheR [EC:2.1.1.80]	Cell Motility - Signal Transduction	1	1
K02557	motB	chemotaxis protein MotB	Cell Motility	1	1
K03406	mcp	methyl-accepting chemotaxis protein	Cell Motility - Signal Transduction	74	4
K03407	cheA	two-component system, chemotaxis family, sensor kinase CheA [EC:2.7.13.3]	Cell Motility - Signal Transduction - Enzyme Families	2	2
K03413	cheY	two-component system, chemotaxis family, response regulator CheY	Cell Motility - Signal Transduction	2	2
K03414	cheZ	chemotaxis protein CheZ	Cell Motility	1	1
ko02040_Flagellar assembly					
K02389	flgD	flagellar basal-body rod modification protein FlgD	Cell Motility	1	1
K02391	flgF	flagellar basal-body rod protein FlgF	Cell Motility	1	1
K02393	flgH	flagellar L-ring protein precursor FlgH	Cell Motility	8	2
K02396	flgK	flagellar hook-associated protein 1 FlgK	Cell Motility	1	1
K02400	flhA	flagellar biosynthesis protein FlhA	Cell Motility - Membrane Transport	1	1
K02407	fliD	flagellar hook-associated protein 2	Cell Motility	2	2
K02413	fliJ	flagellar FliJ protein	Cell Motility	1	1
K02557	motB	chemotaxis protein MotB	Cell Motility	1	1

ko02060_Phosphotransferase system (PTS)

K02761	PTS-Cel-EIIC, celB	PTS system, cellobiose-specific IIC component	Membrane Transport	2	1
K02770	PTS-Fru-EIIC, fruA	PTS system, fructose-specific IIC component	Carbohydrate Metabolism - Membrane Transport	1	1
K02791	PTS-Mal-EIIC, malX	PTS system, maltose and glucose-specific IIC component	Carbohydrate Metabolism - Membrane Transport	1	1
K02795	PTS-Man-EIIC, manY	PTS system, mannose-specific IIC component	Carbohydrate Metabolism - Membrane Transport	1	1

ko03010_Ribosome

K02877	RP-L15e, RPL15	large subunit ribosomal protein L15e	Translation	1	1
K02890	RP-L22, rplV	large subunit ribosomal protein L22	Translation	1	1
K02896	RP-L24e, RPL24	large subunit ribosomal protein L24e	Translation	1	1
K02912	RP-L32e, RPL32	large subunit ribosomal protein L32e	Translation	1	1
K02913	RP-L33, rpmG	large subunit ribosomal protein L33	Translation	1	1
K02935	RP-L7, rplL	large subunit ribosomal protein L7/L12	Translation	2	1
K02936	RP-L7Ae, RPL7A	large subunit ribosomal protein L7Ae	Translation	3	1
K02945	RP-S1, rpsA	small subunit ribosomal protein S1	Translation	17	3
K02946	RP-S10, rpsJ	small subunit ribosomal protein S10	Translation	1	1
K02948	RP-S11, rpsK	small subunit ribosomal protein S11	Translation	1	1
K02965	RP-S19, rpsS	small subunit ribosomal protein S19	Translation	1	1
K02968	RP-S20, rpsT	small subunit ribosomal protein S20	Translation	4	2
K02970	RP-S21, rpsU	small subunit ribosomal protein S21	Translation	18	4
K02986	RP-S4, rpsD	small subunit ribosomal protein S4	Translation	2	2
K02995	RP-S8e, RPS8	small subunit ribosomal protein S8e	Translation	1	1

ko03013_RNA transport

K00974	cca	tRNA nucleotidyltransferase (CCA-adding enzyme) [EC:2.7.7.72 3.1.3.- 3.1.4.-]	Translation	15	6

ko03018_RNA degradation

K00962	pnp, PNPT1	polyribonucleotide nucleotidyltransferase [EC:2.7.7.8]	Folding, Sorting and Degradation - Nucleotide Metabolism	1	1
K00970	pcnB	poly(A) polymerase [EC:2.7.7.19]	Folding, Sorting and Degradation	7	4
K04043	dnaK	molecular chaperone DnaK	Folding, Sorting and Degradation - Infectious Diseases	109	6
K04077	groEL, HSPD1	chaperonin GroEL	Metabolic Diseases - Folding, Sorting and Degradation - Infectious Diseases	1783	8
K05592	deaD	ATP-dependent RNA helicase DeaD [EC:3.6.4.13]	Folding, Sorting and Degradation - Translation	1	1
K08300	rne	ribonuclease E [EC:3.1.26.12]	Folding, Sorting and Degradation - Translation	6	2
K12573	rnr, vacB	ribonuclease R [EC:3.1.-.-]	Folding, Sorting and Degradation	3	3

ko03020_RNA polymerase

K02405	fliA	RNA polymerase sigma factor for flagellar operon FliA	Cell Motility - Transcription - Signal Transduction - Infectious Diseases	7	2
K03040	rpoA	DNA-directed RNA polymerase subunit alpha [EC:2.7.7.6]	Transcription - Nucleotide Metabolism - Replication and Repair	40	7
K03041	rpoA1	DNA-directed RNA polymerase subunit A' [EC:2.7.7.6]	Transcription - Nucleotide Metabolism	5	2
K03042	rpoA2	DNA-directed RNA polymerase subunit A" [EC:2.7.7.6]	Transcription - Nucleotide Metabolism	6	1
K03043	rpoB	DNA-directed RNA polymerase subunit beta [EC:2.7.7.6]	Transcription - Nucleotide Metabolism - Replication and Repair	5	5
K03044	rpoB1	DNA-directed RNA polymerase subunit B' [EC:2.7.7.6]	Transcription - Nucleotide Metabolism	4	3
K03045	rpoB2	DNA-directed RNA polymerase subunit B" [EC:2.7.7.6]	Transcription - Nucleotide Metabolism	2	1
K03046	rpoC	DNA-directed RNA polymerase subunit beta' [EC:2.7.7.6]	Transcription - Nucleotide Metabolism - Replication and Repair	7	4
K03053	rpoH	DNA-directed RNA polymerase subunit H [EC:2.7.7.6]	Transcription - Nucleotide Metabolism	1	1
K03056	rpoL	DNA-directed RNA polymerase subunit L [EC:2.7.7.6]	Transcription - Nucleotide Metabolism	2	1
K03060	rpoZ	DNA-directed RNA polymerase subunit omega [EC:2.7.7.6]	Transcription - Nucleotide Metabolism - Replication and Repair	1	1
K03086	SIG1, rpoD	RNA polymerase primary sigma factor	Transcription	265	6
K03087	SIG2, rpoS	RNA polymerase nonessential primary-like sigma factor	Transcription - Infectious Diseases	30	3
K03088	SIG3.2, rpoE	RNA polymerase sigma-70 factor, ECF subfamily	Transcription	20	3
K03089	SIG3.3.1, rpoH	RNA polymerase sigma-32 factor	Transcription	2	1
K03090	SIG3.3.2, sigB	RNA polymerase sigma-B factor	Transcription	2	2
K03091	SIG3.4	RNA polymerase sporulation-specific sigma factor	Transcription	2	2
K03092	SIG54, rpoN	RNA polymerase sigma-54 factor	Transcription - Signal Transduction - Infectious Diseases	8	1
K03093	SIGMA70	RNA polymerase sigma factor	Transcription	1	1
K13798	K13798, rpoB	DNA-directed RNA polymerase subunit B [EC:2.7.7.6]	Transcription - Nucleotide Metabolism	3	2

ko03022_Basal transcription factors

K03120	TBP, tbp	transcription initiation factor TFIID TATA-box-binding protein	Transcription - Neurodegenerative Diseases	3	1
K03124	TFIIB, GTF2B, SUA7, tfb	transcription initiation factor TFIIB	Transcription	11	3
K10843	ERCC3, XPB	DNA excision repair protein ERCC-3 [EC:3.6.4.12]	Transcription - Replication and Repair	4	2

ko03030_DNA replication

K01972	E6.5.1.2, ligA, ligB	DNA ligase (NAD+) [EC:6.5.1.2]	Replication and Repair	10	2
K02314	dnaB	replicative DNA helicase [EC:3.6.4.12]	Cell Growth and Death - Replication and Repair	1499	9
K02316	dnaG	DNA primase [EC:2.7.7.-]	Replication and Repair	169	6
K02335	DPO1, polA	DNA polymerase I [EC:2.7.7.7]	Nucleotide Metabolism - Replication and Repair	921	8
K02337	DPO3A1, dnaE	DNA polymerase III subunit alpha [EC:2.7.7.7]	Nucleotide Metabolism - Replication and Repair	178	7
K02338	DPO3B, dnaN	DNA polymerase III subunit beta [EC:2.7.7.7]	Nucleotide Metabolism - Replication and Repair	18	2
K02340	DPO3D1, holA	DNA polymerase III subunit delta [EC:2.7.7.7]	Nucleotide Metabolism - Replication and Repair	1	1
K02341	DPO3D2, holB	DNA polymerase III subunit delta' [EC:2.7.7.7]	Nucleotide Metabolism - Replication and Repair	1	1
K02342	DPO3E, dnaQ	DNA polymerase III subunit epsilon [EC:2.7.7.7]	Nucleotide Metabolism - Replication and Repair	16	3
K02343	DPO3G, dnaX	DNA polymerase III subunit gamma/tau [EC:2.7.7.7]	Nucleotide Metabolism - Replication and Repair	13	3
K03111	ssb	single-strand DNA-binding protein	Replication and Repair	222	8
K03469	E3.1.26.4A, RNASEH1, rnhA	ribonuclease HI [EC:3.1.26.4]	Replication and Repair	3	2
K03470	rnhB	ribonuclease HII [EC:3.1.26.4]	Replication and Repair	3	3
K03763	DPO3A2, polC	DNA polymerase III subunit alpha, Gram-positive type [EC:2.7.7.7]	Nucleotide Metabolism - Replication and Repair	9	3
K10747	LIG1	DNA ligase 1 [EC:6.5.1.1]	Replication and Repair	1	1

ko03050_Proteasome

K03420	psmR	proteasome regulatory subunit	Folding, Sorting and Degradation	1	1
K03433	psmB, prcB	proteasome beta subunit [EC:3.4.25.1]	Folding, Sorting and Degradation - Enzyme Families	7	2

ko03060_Protein export

K03070	secA	preprotein translocase subunit SecA	Folding, Sorting and Degradation - Membrane Transport	3	3
K03073	secE	preprotein translocase subunit SecE	Folding, Sorting and Degradation - Membrane Transport	1	1
K03106	SRP54, ffh	signal recognition particle subunit SRP54	Folding, Sorting and Degradation - Membrane Transport	1	1
K03110	ftsY	fused signal recognition particle receptor	Folding, Sorting and Degradation - Membrane Transport	2	2
K03217	yidC, spoIIIJ, OXA1	preprotein translocase subunit YidC	Folding, Sorting and Degradation - Membrane Transport	1	1

ko03070_Bacterial secretion system

K02453	gspD	general secretion pathway protein D	Membrane Transport	3	2
K03070	secA	preprotein translocase subunit SecA	Folding, Sorting and Degradation - Membrane Transport	3	3
K03073	secE	preprotein translocase subunit SecE	Folding, Sorting and Degradation - Membrane Transport	1	1
K03106	SRP54, ffh	signal recognition particle subunit SRP54	Folding, Sorting and Degradation - Membrane Transport	1	1
K03110	ftsY	fused signal recognition particle receptor	Folding, Sorting and Degradation - Membrane Transport	2	2
K03196	virB11	type IV secretion system protein VirB11	Membrane Transport	2	1
K03217	yidC, spoIIIJ, OXA1	preprotein translocase subunit YidC	Folding, Sorting and Degradation - Membrane Transport	1	1
K03223	yscL	type III secretion protein SctL	Membrane Transport	1	1
K11891	impL, vasK, icmF	type VI secretion system protein ImpL	Membrane Transport	2	2
K11904	vgrG	type VI secretion system secreted protein VgrG	Membrane Transport	5	2
K11907	vasG, clpV	type VI secretion system protein VasG	Membrane Transport	4	2

ko03320_PPAR signaling pathway

K00249	E1.3.99.3, ACADM, acd	acyl-CoA dehydrogenase [EC:1.3.99.3]	Carbohydrate Metabolism - Metabolism of Other Amino Acids - Endocrine System - Amino Acid Metabolism - Lipid Metabolism	1	1
K00507	SCD, desC	stearoyl-CoA desaturase (delta-9 desaturase) [EC:1.14.19.1]	Endocrine System - Lipid Metabolism	13	2

K00864	E2.7.1.30, glpK	glycerol kinase [EC:2.7.1.30]	Environmental Adaptation - Endocrine System - Lipid Metabolism	3	3
K01897	ACSL, fadD	long-chain acyl-CoA synthetase [EC:6.2.1.3]	Endocrine System - Transport and Catabolism - Lipid Metabolism	4	2
ko03410_Base excision repair					
K01971	E6.5.1.1, lig	DNA ligase (ATP) [EC:6.5.1.1]	Replication and Repair	71	5
K01972	E6.5.1.2, ligA, ligB	DNA ligase (NAD+) [EC:6.5.1.2]	Replication and Repair	10	2
K02335	DPO1, polA	DNA polymerase I [EC:2.7.7.7]	Nucleotide Metabolism - Replication and Repair	921	8
K03575	mutY	A/G-specific adenine glycosylase [EC:3.2.2.-]	Replication and Repair	4	2
K03648	UNG, UDG	uracil-DNA glycosylase [EC:3.2.2.-]	Immune System Diseases - Replication and Repair	119	3
K07462	recJ	single-stranded-DNA-specific exonuclease [EC:3.1.-.-]	Replication and Repair	4	2
K10563	mutM, fpg	formamidopyrimidine-DNA glycosylase [EC:3.2.2.23 4.2.99.18]	Replication and Repair	2	1
K10747	LIG1	DNA ligase 1 [EC:6.5.1.1]	Replication and Repair	1	1
K10773	NTH	endonuclease III [EC:4.2.99.18]	Replication and Repair	3	2
ko03420_Nucleotide excision repair					
K01971	E6.5.1.1, lig	DNA ligase (ATP) [EC:6.5.1.1]	Replication and Repair	71	5
K01972	E6.5.1.2, ligA, ligB	DNA ligase (NAD+) [EC:6.5.1.2]	Replication and Repair	10	2
K02335	DPO1, polA	DNA polymerase I [EC:2.7.7.7]	Nucleotide Metabolism - Replication and Repair	921	8
K03657	uvrD, pcrA	DNA helicase II / ATP-dependent DNA helicase PcrA [EC:3.6.4.12]	Replication and Repair	110	3
K03701	uvrA	excinuclease ABC subunit A	Replication and Repair	5	2
K03702	uvrB	excinuclease ABC subunit B	Replication and Repair	5	2
K03703	uvrC	excinuclease ABC subunit C	Replication and Repair	78	2
K03723	mfd	transcription-repair coupling factor (superfamily II helicase) [EC:3.6.4.-]	Replication and Repair	2	1
K10747	LIG1	DNA ligase 1 [EC:6.5.1.1]	Replication and Repair	1	1
K10843	ERCC3, XPB	DNA excision repair protein ERCC-3 [EC:3.6.4.12]	Transcription - Replication and Repair	4	2
ko03430_Mismatch repair					
K01141	E3.1.11.1, sbcB	exodeoxyribonuclease I [EC:3.1.11.1]	Replication and Repair	1	1
K01971	E6.5.1.1, lig	DNA ligase (ATP) [EC:6.5.1.1]	Replication and Repair	71	5
K01972	E6.5.1.2, ligA, ligB	DNA ligase (NAD+) [EC:6.5.1.2]	Replication and Repair	10	2
K02337	DPO3A1, dnaE	DNA polymerase III subunit alpha [EC:2.7.7.7]	Nucleotide Metabolism - Replication and Repair	178	7
K02338	DPO3B, dnaN	DNA polymerase III subunit beta [EC:2.7.7.7]	Nucleotide Metabolism - Replication and Repair	18	2
K02340	DPO3D1, holA	DNA polymerase III subunit delta [EC:2.7.7.7]	Nucleotide Metabolism - Replication and Repair	1	1
K02341	DPO3D2, holB	DNA polymerase III subunit delta' [EC:2.7.7.7]	Nucleotide Metabolism - Replication and Repair	1	1
K02342	DPO3E, dnaQ	DNA polymerase III subunit epsilon [EC:2.7.7.7]	Nucleotide Metabolism - Replication and Repair	16	3
K02343	DPO3G, dnaX	DNA polymerase III subunit gamma/tau [EC:2.7.7.7]	Nucleotide Metabolism - Replication and Repair	13	3
K03111	ssb	single-strand DNA-binding protein	Replication and Repair	222	8
K03555	mutS	DNA mismatch repair protein MutS	Replication and Repair	3	3
K03572	mutL	DNA mismatch repair protein MutL	Replication and Repair	3	1
K03601	xseA	exodeoxyribonuclease VII large subunit [EC:3.1.11.6]	Replication and Repair	1	1
K03657	uvrD, pcrA	DNA helicase II / ATP-dependent DNA helicase PcrA [EC:3.6.4.12]	Replication and Repair	110	3
K03763	DPO3A2, polC	DNA polymerase III subunit alpha, Gram-positive type [EC:2.7.7.7]	Nucleotide Metabolism - Replication and Repair	9	3
K06223	dam	DNA adenine methylase [EC:2.1.1.72]	Replication and Repair	127	8
K07456	mutS2	DNA mismatch repair protein MutS2	Replication and Repair	1	1
K07462	recJ	single-stranded-DNA-specific exonuclease [EC:3.1.-.-]	Replication and Repair	4	2
K10747	LIG1	DNA ligase 1 [EC:6.5.1.1]	Replication and Repair	1	1
K10857	exoX	exodeoxyribonuclease X [EC:3.1.11.-]	Replication and Repair	1	1
ko03440_Homologous recombination					
K01159	ruvC	crossover junction endodeoxyribonuclease RuvC [EC:3.1.22.4]	Replication and Repair	76	2
K02335	DPO1, polA	DNA polymerase I [EC:2.7.7.7]	Nucleotide Metabolism - Replication and Repair	921	8
K02337	DPO3A1, dnaE	DNA polymerase III subunit alpha [EC:2.7.7.7]	Nucleotide Metabolism - Replication and Repair	178	7
K02338	DPO3B, dnaN	DNA polymerase III subunit beta [EC:2.7.7.7]	Nucleotide Metabolism - Replication and Repair	18	2
K02340	DPO3D1, holA	DNA polymerase III subunit delta [EC:2.7.7.7]	Nucleotide Metabolism - Replication and Repair	1	1
K02341	DPO3D2, holB	DNA polymerase III subunit delta' [EC:2.7.7.7]	Nucleotide Metabolism - Replication and Repair	1	1
K02342	DPO3E, dnaQ	DNA polymerase III subunit epsilon [EC:2.7.7.7]	Nucleotide Metabolism - Replication and Repair	16	3
K02343	DPO3G, dnaX	DNA polymerase III subunit gamma/tau [EC:2.7.7.7]	Nucleotide Metabolism - Replication and Repair	13	3
K03111	ssb	single-strand DNA-binding protein	Replication and Repair	222	8
K03551	ruvB	holliday junction DNA helicase RuvB	Replication and Repair	4	2
K03553	recA	recombination protein RecA	Replication and Repair	335	6
K03581	recD	exodeoxyribonuclease V alpha subunit [EC:3.1.11.5]	Replication and Repair	6	1
K03582	recB	exodeoxyribonuclease V beta subunit [EC:3.1.11.5]	Replication and Repair	2	2
K03629	recF	DNA replication and repair protein RecF	Replication and Repair	1	1
K03655	recG	ATP-dependent DNA helicase RecG [EC:3.6.4.12]	Replication and Repair	11	3
K03763	DPO3A2, polC	DNA polymerase III subunit alpha, Gram-positive type [EC:2.7.7.7]	Nucleotide Metabolism - Replication and Repair	9	3
K04066	priA	primosomal protein N' (replication factor Y) (superfamily II helicase) [EC:3.6.4.-]	Replication and Repair	1	1
K07462	recJ	single-stranded-DNA-specific exonuclease [EC:3.1.-.-]	Replication and Repair	4	2
ko03450_Non-homologous end-joining					
K01971	E6.5.1.1, lig	DNA ligase (ATP) [EC:6.5.1.1]	Replication and Repair	71	5
ko04011_MAPK signaling pathway - yeast					
K01759	E4.4.1.5, GLO1, gloA	lactoylglutathione lyase [EC:4.4.1.5]	Carbohydrate Metabolism - Signal Transduction	8	1
ko04070_Phosphatidylinositol signaling system					
K01092	E3.1.3.25, IMPA, suhB	myo-inositol-1(or 4)-monophosphatase [EC:3.1.3.25]	Carbohydrate Metabolism - Signal Transduction - Biosynthesis of Other Secondary Metabolites	3	2
ko04112_Cell cycle - Caulobacter					
K01338	E3.4.21.53, lon	ATP-dependent Lon protease [EC:3.4.21.53]	Enzyme Families - Cell Growth and Death	5	3
K01358	clpP, CLPP	ATP-dependent Clp protease, protease subunit [EC:3.4.21.92]	Enzyme Families - Cell Growth and Death	163	6
K02313	dnaA	chromosomal replication initiator protein	Signal Transduction - Cell Growth and Death - Replication and Repair	470	2
K02314	dnaB	replicative DNA helicase [EC:3.6.4.12]	Cell Growth and Death - Replication and Repair	1499	9
K03544	clpX, CLPX	ATP-dependent Clp protease ATP-binding subunit ClpX	Folding, Sorting and Degradation - Cell Growth and Death	25	6
K03588	ftsW, spoVE	cell division protein FtsW	Cell Growth and Death - Replication and Repair	1	1
K03590	ftsA	cell division protein FtsA	Cell Growth and Death - Replication and Repair	1	1
K13581	ccrM	modification methylase [EC:2.1.1.72]	Cell Growth and Death	18	6
ko04113_Meiosis - yeast					
K01768	E4.6.1.1	adenylate cyclase [EC:4.6.1.1]	Nucleotide Metabolism - Cell Growth and Death	61	5
ko04115_p53 signaling pathway					
K10808	RRM2	ribonucleoside-diphosphate reductase subunit M2 [EC:1.17.4.1]	Metabolism of Other Amino Acids - Nucleotide Metabolism - Cell Growth and Death - Replication and Repair	67	5
ko04122_Sulfur relay system					
K00566	mnmA, trmU, TRMU	tRNA-specific 2-thiouridylase [EC:2.8.1.-]	Folding, Sorting and Degradation	4	3
K03154	thiS	sulfur carrier protein	Folding, Sorting and Degradation	1	1
K03639	MOCS1, moaA	molybdenum cofactor biosynthesis protein	Folding, Sorting and Degradation - Metabolism of Cofactors and Vitamins	1	1
K04487	iscS, NFS1	cysteine desulfurase [EC:2.8.1.7]	Folding, Sorting and Degradation - Metabolism of Cofactors and Vitamins	4	3
K11179	tusE, dsrC	tRNA 2-thiouridine synthesizing protein E [EC:2.8.1.-]	Folding, Sorting and Degradation	1	1
ko04141_Protein processing in endoplasmic reticulum					
K04079	htpG, HSP90A	molecular chaperone HtpG	Cancers - Environmental Adaptation - Immune System - Folding, Sorting and Degradation - Endocrine System	2	2
K13993	HSP20	HSP20 family protein	Folding, Sorting and Degradation	10	2

ko04142_Lysosome					
K12373	HEXA_B	hexosaminidase [EC:3.2.1.52]	Carbohydrate Metabolism - Folding, Sorting and Degradation - Glycan Biosynthesis and Metabolism - Transport and Catabolism	1	1
ko04146_Peroxisome					
K00273	E1.4.3.3, DAO	D-amino-acid oxidase [EC:1.4.3.3]	Metabolism of Other Amino Acids - Biosynthesis of Other Secondary Metabolites - Transport and Catabolism - Amino Acid Metabolism	2	1
K00869	E2.7.1.36, MVK, mvaK1	mevalonate kinase [EC:2.7.1.36]	Transport and Catabolism - Metabolism of Terpenoids and Polyketides	1	1
K01640	E4.1.3.4, HMGCL, hmgL	hydroxymethylglutaryl-CoA lyase [EC:4.1.3.4]	Carbohydrate Metabolism - Transport and Catabolism - Metabolism of Terpenoids and Polyketides - Amino Acid Metabolism - Lipid Metabolism	18	1
K01897	ACSL, fadD	long-chain acyl-CoA synthetase [EC:6.2.1.3]	Endocrine System - Transport and Catabolism - Lipid Metabolism	4	2
K03781	katE, CAT	catalase [EC:1.11.1.6]	Neurodegenerative Diseases - Transport and Catabolism - Amino Acid Metabolism - Energy Metabolism	1	1
K04564	E1.15.1.1A, sodA, sodB, SOD2	superoxide dismutase, Fe-Mn family [EC:1.15.1.1]	Neurodegenerative Diseases - Transport and Catabolism	126	5
K04565	E1.15.1.1C, sodC, SOD1	Cu/Zn superoxide dismutase [EC:1.15.1.1]	Neurodegenerative Diseases - Transport and Catabolism	1	1
ko04210_Apoptosis					
K01173	E3.1.30.-	endonuclease [EC:3.1.30.-]	Cell Growth and Death	9	2
ko04260_Cardiac muscle contraction					
K00412	CYTB, petB	ubiquinol-cytochrome c reductase cytochrome b subunit [EC:1.10.2.2]	Circulatory System - Neurodegenerative Diseases - Energy Metabolism	7	2
ko04510_Focal adhesion					
K06236	COL1AS	collagen, type I/II/III/V/XI, alpha	Digestive System - Signaling Molecules and Interaction - Infectious Diseases - Cell Communication	15	3
K06237	COL4A	collagen, type IV, alpha	Cancers - Digestive System - Signaling Molecules and Interaction - Infectious Diseases - Cell Communication	7	2
ko04512_ECM-receptor interaction					
K06236	COL1AS	collagen, type I/II/III/V/XI, alpha	Digestive System - Signaling Molecules and Interaction - Infectious Diseases - Cell Communication	15	3
K06237	COL4A	collagen, type IV, alpha	Cancers - Digestive System - Signaling Molecules and Interaction - Infectious Diseases - Cell Communication	7	2
ko04610_Complement and coagulation cascades					
K01344	PROC	protein C (activated) [EC:3.4.21.69]	Immune System - Enzyme Families	1	1
ko04612_Antigen processing and presentation					
K04079	htpG, HSP90A	molecular chaperone HtpG	Cancers - Environmental Adaptation - Immune System - Folding, Sorting and Degradation - Endocrine System	2	2
ko04621_NOD-like receptor signaling pathway					
K04079	htpG, HSP90A	molecular chaperone HtpG	Cancers - Environmental Adaptation - Immune System - Folding, Sorting and Degradation - Endocrine System	2	2
ko04626_Plant-pathogen interaction					
K00864	E2.7.1.30, glpK	glycerol kinase [EC:2.7.1.30]	Environmental Adaptation - Endocrine System - Lipid Metabolism	3	3
K02358	EF-TU, tufA	elongation factor EF-Tu [EC:3.6.5.3]	Environmental Adaptation - Translation	2	2
K04079	htpG, HSP90A	molecular chaperone HtpG	Cancers - Environmental Adaptation - Immune System - Folding, Sorting and Degradation - Endocrine System	2	2
K13472	raxST	sulfotransferase	Environmental Adaptation	199	5
ko04724_Glutamatergic synapse					
K01915	E6.3.1.2, glnA	glutamine synthetase [EC:6.3.1.2]	Signal Transduction - Nervous System - Amino Acid Metabolism - Energy Metabolism	113	7
ko04910_Insulin signaling pathway					
K00688	E2.4.1.1, glgP, PYG	starch phosphorylase [EC:2.4.1.1]	Carbohydrate Metabolism - Endocrine System	9	3
ko04914_Progesterone-mediated oocyte maturation					
K04079	htpG, HSP90A	molecular chaperone HtpG	Cancers - Environmental Adaptation - Immune System - Folding, Sorting and Degradation - Endocrine System	2	2
ko04920_Adipocytokine signaling pathway					
K01897	ACSL, fadD	long-chain acyl-CoA synthetase [EC:6.2.1.3]	Endocrine System - Transport and Catabolism - Lipid Metabolism	4	2
ko04940_Type I diabetes mellitus					
K04077	groEL, HSPD1	chaperonin GroEL	Metabolic Diseases - Folding, Sorting and Degradation - Infectious Diseases	1783	8
ko04964_Proximal tubule bicarbonate reclamation					
K00261	E1.4.1.3	glutamate dehydrogenase (NAD(P)+) [EC:1.4.1.3]	Metabolism of Other Amino Acids - Excretory System - Amino Acid Metabolism - Energy Metabolism	3	2
ko04973_Carbohydrate digestion and absorption					
K01176	E3.2.1.1, amyA, malS	alpha-amylase [EC:3.2.1.1]	Carbohydrate Metabolism - Digestive System	2	1
ko04974_Protein digestion and absorption					
K06236	COL1AS	collagen, type I/II/III/V/XI, alpha	Digestive System - Signaling Molecules and Interaction - Infectious Diseases - Cell Communication	15	3
K06237	COL4A	collagen, type IV, alpha	Cancers - Digestive System - Signaling Molecules and Interaction - Infectious Diseases - Cell Communication	7	2
ko04978_Mineral absorption					
K00510	HMOX, hmuO, ho	heme oxygenase [EC:1.14.99.3]	Digestive System - Metabolism of Cofactors and Vitamins	2	1
ko05010_Alzheimer's disease					
K00134	GAPDH, gapA	glyceraldehyde 3-phosphate dehydrogenase [EC:1.2.1.12]	Carbohydrate Metabolism - Neurodegenerative Diseases	3	2
K00412	CYTB, petB	ubiquinol-cytochrome c reductase cytochrome b subunit [EC:1.10.2.2]	Circulatory System - Neurodegenerative Diseases - Energy Metabolism	7	2
ko05012_Parkinson's disease					
K00412	CYTB, petB	ubiquinol-cytochrome c reductase cytochrome b subunit [EC:1.10.2.2]	Circulatory System - Neurodegenerative Diseases - Energy Metabolism	7	2
ko05014_Amyotrophic lateral sclerosis (ALS)					
K03781	katE, CAT	catalase [EC:1.11.1.6]	Neurodegenerative Diseases - Transport and Catabolism - Amino Acid Metabolism - Energy Metabolism	1	1
K04565	E1.15.1.1C, sodC, SOD1	Cu/Zn superoxide dismutase [EC:1.15.1.1]	Neurodegenerative Diseases - Transport and Catabolism	1	1
ko05016_Huntington's disease					
K00412	CYTB, petB	ubiquinol-cytochrome c reductase cytochrome b subunit [EC:1.10.2.2]	Circulatory System - Neurodegenerative Diseases - Energy Metabolism	7	2
K03120	TBP, tbp	transcription initiation factor TFIID TATA-box-binding protein	Transcription - Neurodegenerative Diseases	3	1

K04564	E1.15.1.1A, sodA, sodB, SOD2	superoxide dismutase, Fe-Mn family [EC:1.15.1.1]	Neurodegenerative Diseases - Transport and Catabolism	126	5
K04565	E1.15.1.1C, sodC, SOD1	Cu/Zn superoxide dismutase [EC:1.15.1.1]	Neurodegenerative Diseases - Transport and Catabolism	1	1
ko05020_Prion diseases					
K04565	E1.15.1.1C, sodC, SOD1	Cu/Zn superoxide dismutase [EC:1.15.1.1]	Neurodegenerative Diseases - Transport and Catabolism	1	1
ko05100_Bacterial invasion of epithelial cells					
K13730	inlA	internalin A	Infectious Diseases	2	2
K13732	fnbA	fibronectin-binding protein A	Infectious Diseases	2	2
K13733	fnbB	fibronectin-binding protein B	Infectious Diseases	1	1
ko05110_Vibrio cholerae infection					
K08604	E3.4.24.25	vibriolysin [EC:3.4.24.25]	Enzyme Families - Infectious Diseases	1	1
ko05111_Vibrio cholerae pathogenic cycle					
K02405	fliA	RNA polymerase sigma factor for flagellar operon FliA	Cell Motility - Transcription - Signal Transduction - Infectious Diseases	7	2
K03087	SIG2, rpoS	RNA polymerase nonessential primary-like sigma factor	Transcription - Infectious Diseases	30	3
K03092	SIG54, rpoN	RNA polymerase sigma-54 factor	Transcription - Signal Transduction - Infectious Diseases	8	1
K07173	luxS	S-ribosylhomocysteine lyase [EC:4.4.1.21]	Infectious Diseases - Amino Acid Metabolism	1	1
K08604	E3.4.24.25	vibriolysin [EC:3.4.24.25]	Enzyme Families - Infectious Diseases	1	1
K08720	ompU	outer membrane protein OmpU	Infectious Diseases	2	1
ko05120_Epithelial cell signaling in Helicobacter pylori infection					
K01428	ureC	urease subunit alpha [EC:3.5.1.5]	Infectious Diseases - Nucleotide Metabolism - Xenobiotics Biodegradation and Metabolism - Amino Acid Metabolism	2	1
ko05142_Chagas disease (American trypanosomiasis)					
K01354	ptrB	oligopeptidase B [EC:3.4.21.83]	Enzyme Families - Infectious Diseases	2	2
ko05143_African trypanosomiasis					
K01354	ptrB	oligopeptidase B [EC:3.4.21.83]	Enzyme Families - Infectious Diseases	2	2
ko05146_Amoebiasis					
K01476	E3.5.3.1, rocF, arg	arginase [EC:3.5.3.1]	Infectious Diseases - Amino Acid Metabolism	11	1
K06236	COL1AS	collagen, type I/II/III/V/XI, alpha	Digestive System - Signaling Molecules and Interaction - Infectious Diseases - Cell Communication	15	3
K06237	COL4A	collagen, type IV, alpha	Cancers - Digestive System - Signaling Molecules and Interaction - Infectious Diseases - Cell Communication	7	2
ko05150_Staphylococcus aureus infection					
K14195	sasG	surface protein G	Infectious Diseases	8	2
K14205	mprF, fmtC	phosphatidylglycerol lysyltransferase [EC:2.3.2.3]	Infectious Diseases	3	1
ko05152_Tuberculosis					
K02040	pstS	phosphate transport system substrate-binding protein	Signal Transduction - Infectious Diseases - Membrane Transport	57	4
K04043	dnaK	molecular chaperone DnaK	Folding, Sorting and Degradation - Infectious Diseases	109	6
K04077	groEL, HSPD1	chaperonin GroEL	Metabolic Diseases - Folding, Sorting and Degradation - Infectious Diseases	1783	8
K14952	namH	UDP-MurNAc hydroxylase	Infectious Diseases	1	1
ko05200_Pathways in cancer					
K04079	htpG, HSP90A	molecular chaperone HtpG	Cancers - Environmental Adaptation - Immune System - Folding, Sorting and Degradation - Endocrine System	2	2
K06237	COL4A	collagen, type IV, alpha	Cancers - Digestive System - Signaling Molecules and Interaction - Infectious Diseases - Cell Communication	7	2
ko05215_Prostate cancer					
K04079	htpG, HSP90A	molecular chaperone HtpG	Cancers - Environmental Adaptation - Immune System - Folding, Sorting and Degradation - Endocrine System	2	2
ko05222_Small cell lung cancer					
K06237	COL4A	collagen, type IV, alpha	Cancers - Digestive System - Signaling Molecules and Interaction - Infectious Diseases - Cell Communication	7	2
ko05322_Systemic lupus erythematosus					
K11089	TROVE2, SSA2	60 kDa SS-A/Ro ribonucleoprotein	Immune System Diseases	5	4
ko05340_Primary immunodeficiency					
K03648	UNG, UDG	uracil-DNA glycosylase [EC:3.2.2.-]	Immune System Diseases - Replication and Repair	119	3
no pathway					
K00046	idnO	gluconate 5-dehydrogenase [EC:1.1.1.69]	Metabolism	1	1
K00096	E1.1.1.261	glycerol-1-phosphate dehydrogenase [NAD(P)] [EC:1.1.1.261]	Metabolism	1	1
K00098	idnD	L-idonate 5-dehydrogenase [EC:1.1.1.264]	Metabolism	1	1
K00153	E1.1.1.306	S-(hydroxymethyl)mycothiol dehydrogenase [EC:1.1.1.306]	Metabolism	1	1
K00344	E1.6.5.5, qor	NADPH2:quinone reductase [EC:1.6.5.5]	Metabolism	2	2
K00346	nqrA	Na+-transporting NADH:ubiquinone oxidoreductase subunit A [EC:1.6.5.-]	Metabolism	1	1
K00355	E1.6.5.2, NQO1	NAD(P)H dehydrogenase (quinone) [EC:1.6.5.2]	Metabolism	1	1
K00428	E1.11.1.5	cytochrome c peroxidase [EC:1.11.1.5]	Cellular Processes and Signaling	2	2
K00435	E1.11.1.-	peroxiredoxin [EC:1.11.1.-]	Genetic Information Processing	6	4
K00518	E1.15.1.1	superoxide dismutase [EC:1.15.1.1]	Cellular Processes and Signaling	2	1
K00528	E1.18.1.2, fpr	ferredoxin--NADP+ reductase [EC:1.18.1.2]	Metabolism	1	1
K00540	E1.-.-.-		Metabolism	5	2
K00561	ermC, ermA	23S rRNA (adenine2085-N6)-dimethyltransferase [EC:2.1.1.184]	Genetic Information Processing	1	1
K00567	E2.1.1.63, MGMT, ogt	methylated-DNA-[protein]-cysteine S-methyltransferase [EC:2.1.1.63]	Replication and Repair	2	1
K00571	E2.1.1.72	site-specific DNA-methyltransferase (adenine-specific) [EC:2.1.1.72]	Genetic Information Processing	212	8
K00573	E2.1.1.77, pcm	protein-L-isoaspartate(D-aspartate) O-methyltransferase [EC:2.1.1.77]	Genetic Information Processing	1	1
K00590	E2.1.1.113	site-specific DNA-methyltransferase (cytosine-N4-specific) [EC:2.1.1.113]	Genetic Information Processing	52	4
K00612	E2.1.3.-	carbamoyltransferase [EC:2.1.3.-]	Genetic Information Processing	221	7
K00633	E2.3.1.18, lacA	galactoside O-acetyltransferase [EC:2.3.1.18]	Metabolism	18	3
K00638	E2.3.1.28, cat	chloramphenicol O-acetyltransferase [EC:2.3.1.28]	Metabolism	14	3
K00661	E2.3.1.79, maa	maltose O-acetyltransferase [EC:2.3.1.79]	Metabolism	6	4
K00666	K00666	fatty-acyl-CoA synthase [EC:6.2.1.-]	Lipid Metabolism	1	1
K00685	ATE1, ate1	arginine-tRNA-protein transferase [EC:2.3.2.8]	Genetic Information Processing	1	1
K00809	E2.5.1.46, dys1	deoxyhypusine synthase [EC:2.5.1.46]	Genetic Information Processing	3	3
K00837	E2.6.1.-		Metabolism	4	3
K00870	E2.7.1.37	protein kinase [EC:2.7.1.37]	Cellular Processes and Signaling	1	1
K00906	aceK	isocitrate dehydrogenase kinase/phosphatase [EC:2.7.11.5 3.1.3.-]	Cellular Processes and Signaling	2	1
K00924	E2.7.1.-		Cellular Processes and Signaling	1	1
K00960	E2.7.7.6	DNA-directed RNA polymerase [EC:2.7.7.6]	Genetic Information Processing	2	1
K00961	E2.7.7.7	DNA polymerase [EC:2.7.7.7]	Genetic Information Processing	4	2
K00996	E2.7.8.6, rfbP	undecaprenyl-phosphate galactose phosphotransferase [EC:2.7.8.6]	Glycan Biosynthesis and Metabolism	1	1
K01043	E2.-.-.-		Metabolism	1	1
K01090	E3.1.3.16	protein phosphatase [EC:3.1.3.16]	Metabolism	4	1

K01104	E3.1.3.48	protein-tyrosine phosphatase [EC:3.1.3.48]	Cellular Processes and Signaling	2	1
K01133	E3.1.6.6, betC	choline-sulfatase [EC:3.1.6.6]	Cellular Processes and Signaling	2	2
K01138	K01138	uncharacterized sulfatase [EC:3.1.6.-]	Cellular Processes and Signaling	1	1
K01143	E3.1.11.3	exodeoxyribonuclease (lambda-induced) [EC:3.1.11.3]	Genetic Information Processing	6	3
K01144	E3.1.11.5	exodeoxyribonuclease V [EC:3.1.11.5]	Genetic Information Processing	3	2
K01153	hsdR	type I restriction enzyme, R subunit [EC:3.1.21.3]	Genetic Information Processing	67	3
K01154	hsdS	type I restriction enzyme, S subunit [EC:3.1.21.3]	Genetic Information Processing	15	5
K01155	E3.1.21.4	type II restriction enzyme [EC:3.1.21.4]	Genetic Information Processing	12	3
K01156	res	type III restriction enzyme [EC:3.1.21.5]	Genetic Information Processing	11	2
K01160	rusA	crossover junction endodeoxyribonuclease RusA [EC:3.1.22.4]	Replication and Repair	20	3
K01161	E3.1.25.1	deoxyribonuclease (pyrimidine dimer) [EC:3.1.25.1]	Metabolism	1	1
K01174	E3.1.31.1, nuc	micrococcal nuclease [EC:3.1.31.1]	Genetic Information Processing	13	2
K01175	E3.1.-.-		Metabolism	2	2
K01181	E3.2.1.8, xynA	endo-1,4-beta-xylanase [EC:3.2.1.8]	Metabolism	21	2
K01185	E3.2.1.17	lysozyme [EC:3.2.1.17]	Metabolism	3297	7
K01200	E3.2.1.41	pullulanase [EC:3.2.1.41]	Metabolism	1	1
K01219	E3.2.1.81	agarase [EC:3.2.1.81]	Metabolism	1	1
K01236	E3.2.1.141	maltooligosyltrehalose trehalohydrolase [EC:3.2.1.141]	Metabolism	1	1
K01238	E3.2.1.-		Metabolism	26	2
K01265	E3.4.11.18, map	methionyl aminopeptidase [EC:3.4.11.18]	Enzyme Families	4	2
K01269	E3.4.11.-	aminopeptidase [EC:3.4.11.-]	Metabolism	1	1
K01284	dcp	peptidyl-dipeptidase Dcp [EC:3.4.15.5]	Enzyme Families	1	1
K01297	ldcA	muramoyltetrapeptide carboxypeptidase [EC:3.4.17.13]	Enzyme Families	1	1
K01299	E3.4.17.19	carboxypeptidase Taq [EC:3.4.17.19]	Enzyme Families	3	2
K01308	E3.4.19.11	g-D-glutamyl-meso-diaminopimelate peptidase [EC:3.4.19.11]	Enzyme Families	1	1
K01317	ACR	acrosin [EC:3.4.21.10]	Enzyme Families	1	1
K01322	E3.4.21.26, PREP	prolyl oligopeptidase [EC:3.4.21.26]	Enzyme Families	1	1
K01356	lexA	repressor LexA [EC:3.4.21.88]	Enzyme Families - Replication and Repair	8	5
K01361	E3.4.21.96	lactocepin [EC:3.4.21.96]	Folding, Sorting and Degradation - Enzyme Families	2	2
K01362	E3.4.21.-		Metabolism	30	2
K01407	ptr	protease III [EC:3.4.24.55]	Enzyme Families	1	1
K01409	E3.4.24.57, gcp	O-sialoglycoprotein endopeptidase [EC:3.4.24.57]	Enzyme Families	1	1
K01417	E3.4.24.-		Metabolism	3	2
K01419	hslV, clpQ	ATP-dependent HslUV protease, peptidase subunit HslV [EC:3.4.25.-]	Enzyme Families	1	1
K01422	E3.4.99.-		Cellular Processes and Signaling	1	1
K01446	E3.5.1.28	N-acetylmuramoyl-L-alanine amidase [EC:3.5.1.28]	Cellular Processes and Signaling	10	1
K01447	E3.5.1.28A, cwlA, xlyA, xlyB	N-acetylmuramoyl-L-alanine amidase [EC:3.5.1.28]	Cellular Processes and Signaling	159	4
K01448	E3.5.1.28B, amiA, amiB, amiC	N-acetylmuramoyl-L-alanine amidase [EC:3.5.1.28]	Replication and Repair	257	3
K01449	E3.5.1.28C, cwlJ, sleB	N-acetylmuramoyl-L-alanine amidase [EC:3.5.1.28]	Cellular Processes and Signaling	3	2
K01462	PDF, def	peptide deformylase [EC:3.5.1.88]	Metabolism	23	6
K01529	E3.6.1.-		Metabolism	1	1
K01533	E3.6.3.4, ATP7, copA	Cu2+-exporting ATPase [EC:3.6.3.4]	Metabolism	3	2
K01537	E3.6.3.8	Ca2+-transporting ATPase [EC:3.6.3.8]	Metabolism	2	2
K01551	E3.6.3.16, arsA	arsenite-transporting ATPase [EC:3.6.3.16]	Cellular Processes and Signaling	4	1
K01669	E4.1.99.3, phrB	deoxyribodipyrimidine photo-lyase [EC:4.1.99.3]	Replication and Repair	1	1
K01724	E4.2.1.96, PCBD, phhB	4a-hydroxytetrahydrobiopterin dehydratase [EC:4.2.1.96]	Metabolism	7	2
K01795	E5.1.3.-		Metabolism	20	6
K01802	E5.2.1.8	peptidylprolyl isomerase [EC:5.2.1.8]	Genetic Information Processing	10	3
K01854	glf	UDP-galactopyranose mutase [EC:5.4.99.9]	Cellular Processes and Signaling	123	5
K01989	ABC.X4.S	putative ABC transport system substrate-binding protein	Membrane Transport	1	1
K01990	ABC-2.A	ABC-2 type transport system ATP-binding protein	Membrane Transport	9	2
K01991	ABC-2.OM, wza	polysaccharide export outer membrane protein	Cellular Processes and Signaling	2	2
K02003	ABC.CD.A		Membrane Transport	3	2
K02004	ABC.CD.P		Membrane Transport	4	2
K02014	ABC.FEV.OM	iron complex outermembrane recepter protein	Cellular Processes and Signaling	6	3
K02023	ABC.MS.A	multiple sugar transport system ATP-binding protein	Membrane Transport	1	1
K02025	ABC.MS.P	multiple sugar transport system permease protein	Membrane Transport	2	2
K02026	ABC.MS.P1	multiple sugar transport system permease protein	Membrane Transport	3	3
K02027	ABC.MS.S	multiple sugar transport system substrate-binding protein	Membrane Transport	3	1
K02055	ABC.SP.S	putative spermidine/putrescine transport system substrate-binding protein	Membrane Transport	1	1
K02058	ABC.SS.S	simple sugar transport system substrate-binding protein	Membrane Transport	4	2
K02065	ABC.X1.A	putative ABC transport system ATP-binding protein	Membrane Transport	1	1
K02066	ABC.X1.P	putative ABC transport system permease protein	Membrane Transport	1	1
K02078	acpP	acyl carrier protein	Metabolism	2	1
K02198	ccmF	cytochrome c-type biogenesis protein CcmF	Cellular Processes and Signaling	1	1
K02238	comEC	competence protein ComEC	Membrane Transport	2	2
K02283	cpaF, tadA	pilus assembly protein CpaF	Cell Motility - Membrane Transport	1	1
K02315	dnaC	DNA replication protein DnaC	Replication and Repair	20	1
K02334	dpo	DNA polymerase bacteriophage-type [EC:2.7.7.7]	Genetic Information Processing	190	7
K02336	DPO2, polB	DNA polymerase II [EC:2.7.7.7]	Replication and Repair	27	2
K02346	DPO4, dinB	DNA polymerase IV [EC:2.7.7.7]	Replication and Repair	1	1
K02355	EF-G, fusA	elongation factor EF-G [EC:3.6.5.3]	Translation	2	2
K02395	flgJ	flagellar protein FlgJ	Cell Motility	47	3
K02404	flhF	flagellar biosynthesis protein FlhF	Cell Motility	1	1
K02437	gcvH	glycine cleavage system H protein	Metabolism	1	1
K02440	GLPF	glycerol uptake facilitator protein	Signaling Molecules and Interaction	1	1
K02469	gyrA	DNA gyrase subunit A [EC:5.99.1.3]	Replication and Repair	18	3
K02470	gyrB	DNA gyrase subunit B [EC:5.99.1.3]	Replication and Repair	15	3
K02481	K02481	two-component system, NtrC family, response regulator	Signal Transduction	1	1
K02493	hemK	methyltransferase [EC:2.1.1.-]	Genetic Information Processing	3	2
K02503	hit	Hit-like protein involved in cell-cycle regulation	Poorly Characterized	1	1
K02518	IF-1, infA	translation initiation factor IF-1	Translation	4	4
K02519	IF-2, infB	translation initiation factor IF-2	Translation	3	2
K02520	IF-3, infC	translation initiation factor IF-3	Translation	6	2
K02528	ksgA	16S rRNA (adenine1518-N6/adenine1519-N6)-dimethyltransferase [EC:2.1.1.182]	Translation	2	1
K02598	nirC	nitrite transporter NirC	Cellular Processes and Signaling	2	1
K02601	nusG	transcriptional antiterminator NusG	Translation	6	2
K02609	paaA	phenylacetic acid degradation protein	Poorly Characterized	11	2
K02622	parE	topoisomerase IV subunit B [EC:5.99.1.-]	Replication and Repair	4	4
K02666	pilQ	type IV pilus assembly protein PilQ	Cell Motility - Membrane Transport	2	1
K02674	pilY1	type IV pilus assembly protein PilY1	Cell Motility - Membrane Transport	2	1
K02687	prmA	ribosomal protein L11 methyltransferase [EC:2.1.1.-]	Genetic Information Processing	1	1
K02715	psbN	PsbN protein	Poorly Characterized	2	1
K02742	sprT	SprT protein	Poorly Characterized	1	1
K02805	rffA, wecE	lipopolysaccharide biosynthesis protein	Amino Acid Metabolism	5	3
K02835	RF-1, prfA	peptide chain release factor RF-1	Translation	1	1
K02837	RF-3, prfC	peptide chain release factor RF-3	Translation	1	1
K03168	topA	DNA topoisomerase I [EC:5.99.1.2]	Replication and Repair	10	3
K03207	wcaH	colanic acid biosynthesis protein WcaH [EC:3.6.1.-]	Metabolism	3	3
K03281	TC.ClC	chloride channel protein, ClC family	Cellular Processes and Signaling	2	1
K03284	TC.MIT	metal ion transporter, MIT family	Cellular Processes and Signaling	8	3
K03286	TC.OOP	OmpA-OmpF porin, OOP family	Cellular Processes and Signaling	3	2
K03292	TC.GPH	glycoside/pentoside/hexuronide:cation symporter, GPH family	Cellular Processes and Signaling	1	1
K03293	TC.AAT	amino acid transporter, AAT family	Cellular Processes and Signaling	1	1
K03294	TC.APA	basic amino acid/polyamine antiporter, APA family	Cellular Processes and Signaling	1	1
K03295	TC.CDF	cation efflux system protein, CDF family	Cellular Processes and Signaling	1	1
K03296	TC.HAE1	hydrophobic/amphiphilic exporter-1 (mainly G- bacteria), HAE1 family	Cellular Processes and Signaling	1	1
K03298	TC.DME	drug/metabolite transporter, DME family	Cellular Processes and Signaling	1	1
K03299	TC.GNTP	gluconate:H+ symporter, GntP family	Cellular Processes and Signaling	1	1
K03303	TC.LCTP	lactate transporter, LctP family	Cellular Processes and Signaling	1	1

K03307	TC.SSS	solute:Na+ symporter, SSS family	Cellular Processes and Signaling	1	1
K03320	TC.AMT	ammonium transporter, Amt family	Cellular Processes and Signaling	2	2
K03328	TC.PST	polysaccharide transporter, PST family	Cellular Processes and Signaling	1	1
K03386	E1.11.1.15, PRDX, ahpC	peroxiredoxin (alkyl hydroperoxide reductase subunit C) [EC:1.11.1.15]	Genetic Information Processing	6	5
K03427	hsdM	type I restriction enzyme M protein [EC:2.1.1.72]	Genetic Information Processing	29	5
K03438	mraW	S-adenosyl-methyltransferase [EC:2.1.1.-]	Cellular Processes and Signaling	2	2
K03439	E2.1.1.33	tRNA (guanine-N7-)-methyltransferase [EC:2.1.1.33]	Metabolism	7	2
K03457	TC.NCS1	nucleobase:cation symporter-1, NCS1 family	Cellular Processes and Signaling	4	1
K03466	ftsK, spoIIIE	DNA segregation ATPase FtsK/SpoIIIE, S-DNA-T family	Replication and Repair	15	3
K03495	gidA	glucose inhibited division protein A	Replication and Repair	1	1
K03496	parA, soj	chromosome partitioning protein	Cell Motility - Replication and Repair	80	5
K03497	parB, spo0J	chromosome partitioning protein, ParB family	Cell Motility - Transcription - Replication and Repair	25	7
K03502	DPO5C, umuC	DNA polymerase V	Replication and Repair	6	2
K03503	DPO5D, umuD	DNA polymerase V [EC:3.4.21.-]	Enzyme Families - Replication and Repair	4	2
K03521	fixA, etfB	electron transfer flavoprotein beta subunit	Metabolism	1	1
K03522	fixB, etfA	electron transfer flavoprotein alpha subunit	Metabolism	2	2
K03523	bioY	putative biotin biosynthesis protein BioY	Poorly Characterized	1	1
K03529	smc	chromosome segregation protein	Replication and Repair	7	1
K03530	hupB	DNA-binding protein HU-beta	Replication and Repair	3	2
K03545	tig	trigger factor	Genetic Information Processing	9	4
K03546	sbcC	exonuclease SbcC	Replication and Repair	55	5
K03547	sbcD	exonuclease SbcD	Replication and Repair	4	2
K03561	exbB	biopolymer transport protein ExbB	Cellular Processes and Signaling	1	1
K03574	MUTT, NUDT1, MTH1	7,8-dihydro-8-oxoguanine triphosphatase [EC:3.6.1.-]	Replication and Repair	12	2
K03578	hrpA	ATP-dependent helicase HrpA [EC:3.6.4.13]	Genetic Information Processing	1	1
K03580	hepA	ATP-dependent helicase HepA [EC:3.6.4.-]	Genetic Information Processing	6	1
K03585	acrA	membrane fusion protein	Replication and Repair	1	1
K03596	lepA	GTP-binding protein LepA	Cellular Processes and Signaling	2	2
K03606	wcaJ	putative colanic acid biosysnthesis UDP-glucose lipid carrier transferase	Cellular Processes and Signaling	1	1
K03624	greA	transcription elongation factor GreA	Genetic Information Processing	1	1
K03625	nusB	N utilization substance protein B	Translation	1	1
K03630	radC	DNA repair protein RadC	Genetic Information Processing	21	5
K03641	tolB	TolB protein	Cellular Processes and Signaling	1	1
K03642	rlpA	rare lipoprotein A	Cellular Processes and Signaling	87	2
K03643	lptE, rlpB	LPS-assembly lipoprotein	Cellular Processes and Signaling	1	1
K03646	tolA	colicin import membrane protein	Cellular Processes and Signaling	5	2
K03656	rep	ATP-dependent DNA helicase Rep [EC:3.6.4.12]	Replication and Repair	1	1
K03664	smpB	SsrA-binding protein	Genetic Information Processing	40	2
K03671	trxA	thioredoxin 1	Folding, Sorting and Degradation	136	4
K03672	trxC	thioredoxin 2 [EC:1.8.1.8]	Folding, Sorting and Degradation	10	2
K03676	grxC, GLRX, GLRX2	glutaredoxin 3	Folding, Sorting and Degradation	42	6
K03684	rnd	ribonuclease D [EC:3.1.13.5]	Genetic Information Processing	5	2
K03686	dnaJ	molecular chaperone DnaJ	Folding, Sorting and Degradation	65	4
K03694	clpA	ATP-dependent Clp protease ATP-binding subunit ClpA	Folding, Sorting and Degradation	26	5
K03695	clpB	ATP-dependent Clp protease ATP-binding subunit ClpB	Folding, Sorting and Degradation	45	6
K03696	clpC	ATP-dependent Clp protease ATP-binding subunit ClpC	Folding, Sorting and Degradation	342	8
K03697	clpE	ATP-dependent Clp protease ATP-binding subunit ClpE	Folding, Sorting and Degradation	7	3
K03704	cspA	cold shock protein (beta-ribbon, CspA family)	Transcription	2	2
K03711	fur	Fur family transcriptional regulator, ferric uptake regulator	Transcription	2	1
K03716	splB	spore photoproduct lyase [EC:4.1.99.-]	Genetic Information Processing	6	2
K03721	tyrR	transcriptional regulator of aroF, aroG, tyrA and aromatic amino acid transport	Transcription	3	1
K03724	lhr	ATP-dependent helicase Lhr and Lhr-like helicase [EC:3.6.4.-]	Replication and Repair	1	1
K03726	helS	helicase [EC:3.6.4.-]	Genetic Information Processing	3	1
K03727	helY	ATP-dependent RNA helicase HelY [EC:3.6.4.-]	Genetic Information Processing	1	1
K03733	xerC	integrase/recombinase XerC	Replication and Repair	25	2
K03743	K03743		Poorly Characterized	4	4
K03744	lemA	LemA protein	Poorly Characterized	1	1
K03750	moeA	molybdopterin biosynthesis protein MoeA	Metabolism	3	2
K03762	proP	MFS transporter, MHS family, proline/betaine transporter	Membrane Transport	1	1
K03767	PPIA	peptidyl-prolyl cis-trans isomerase A (cyclophilin A) [EC:5.2.1.8]	Folding, Sorting and Degradation	2	2
K03768	PPIB, ppiB	peptidyl-prolyl cis-trans isomerase B (cyclophilin B) [EC:5.2.1.8]	Folding, Sorting and Degradation	24	2
K03771	surA	peptidyl-prolyl cis-trans isomerase SurA [EC:5.2.1.8]	Folding, Sorting and Degradation	1	1
K03774	slpA	FKBP-type peptidyl-prolyl cis-trans isomerase SlpA [EC:5.2.1.8]	Folding, Sorting and Degradation	7	3
K03775	slyD	FKBP-type peptidyl-prolyl cis-trans isomerase SlyD [EC:5.2.1.8]	Folding, Sorting and Degradation	7	1
K03789	rimI	ribosomal-protein-alanine N-acetyltransferase [EC:2.3.1.128]	Translation	3	2
K03791	K03791	putative chitinase	Poorly Characterized	1162	6
K03796	bax	Bax protein	Poorly Characterized	45	3
K03797	E3.4.21.102, prc, ctpA	carboxyl-terminal processing protease [EC:3.4.21.102]	Enzyme Families	1	1
K03798	ftsH, hflB	cell division protease FtsH [EC:3.4.24.-]	Folding, Sorting and Degradation - Enzyme Families	3	2
K03802	cphA	cyanophycin synthetase [EC:6.-.-.-]	Cellular Processes and Signaling	4	1
K03806	ampD	AmpD protein	Cellular Processes and Signaling	2	1
K03818	wcaF	putative colanic acid biosynthesis acetyltransferase WcaF [EC:2.3.1.-]	Metabolism	1	1
K03832	tonB	periplasmic protein TonB	Cellular Processes and Signaling	3	2
K03839	fldA	flavodoxin I	Metabolism	6	1
K03840	fldB	flavodoxin II	Metabolism	1	1
K03924	moxR	MoxR-like ATPase [EC:3.6.3.-]	Metabolism	16	4
K03977	engA	GTP-binding protein	Translation	3	1
K03979	obg	GTP-binding protein	Translation	2	1
K04024	eutJ	ethanolamine utilization protein EutJ	Metabolism	1	1
K04047	dps	starvation-inducible DNA-binding protein	Replication and Repair	66	2
K04068	nrdG	anaerobic ribonucleoside-triphosphate reductase activating protein [EC:1.97.1.4]	Genetic Information Processing	4	2
K04075	tilS, mesJ	tRNA(Ile)-lysidine synthase [EC:6.3.4.-]	Genetic Information Processing	2	2
K04078	groES, HSPE1	chaperonin GroES	Folding, Sorting and Degradation	308	4
K04080	ibpA	molecular chaperone IbpA	Folding, Sorting and Degradation	78	6
K04090	E1.2.7.8	indolepyruvate ferredoxin oxidoreductase [EC:1.2.7.8]	Metabolism	1	1
K04091	ssuD	alkanesulfonate monooxygenase [EC:1.14.14.5]	Metabolism	3	2
K04095	fic	cell filamentation protein	Replication and Repair	1	1
K04096	smf	DNA processing protein	Genetic Information Processing	1	1
K04338	csgF	curli production assembly/transport component CsgF	Membrane Transport	1	1
K04483	radA	DNA repair protein RadA	Replication and Repair	1	1
K04485	sms, radA	DNA repair protein RadA/Sms	Replication and Repair	110	2
K04488	iscU, nifU	nitrogen fixation protein NifU and related proteins	Metabolism	97	5
K04651	hypA	hydrogenase nickel incorporation protein HypA	Genetic Information Processing	1	1
K04652	hypB	hydrogenase nickel incorporation protein HypB	Genetic Information Processing	1	1
K04654	hypD	hydrogenase expression/formation protein HypD	Genetic Information Processing	5	1
K04744	lptD, imp, ostA	LPS-assembly protein	Cellular Processes and Signaling	1	1
K04750	phnB	PhnB protein	Poorly Characterized	1	1
K04756	ahpD	alkyl hydroperoxide reductase subunit D	Poorly Characterized	1	1
K04758	feoA	ferrous iron transport protein A	Cellular Processes and Signaling	1	1
K04759	feoB	ferrous iron transport protein B	Cellular Processes and Signaling	1	1
K04763	xerD	integrase/recombinase XerD	Replication and Repair	58	6
K04768	acuC	acetoin utilization protein AcuC	Metabolism	4	2
K04773	sppA	protease IV [EC:3.4.21.-]	Enzyme Families	10	2
K04800	rfcL	replication factor C large subunit	Replication and Repair	3	2
K04801	rfcS	replication factor C small subunit	Replication and Repair	456	7
K05303	E2.1.1.101	macrocin O-methyltransferase [EC:2.1.1.101]	Metabolism	7	2
K05337	fer	ferredoxin	Metabolism	9	3
K05516	cbpA	curved DNA-binding protein	Folding, Sorting and Degradation - Replication and Repair	27	5
K05520	pfpI	protease I [EC:3.2.-.-]	Enzyme Families	10	2
K05521	draG	ADP-ribosylglycohydrolase [EC:3.2.-.-]	Genetic Information Processing	8	2
K05524	fdxA	ferredoxin	Metabolism	12	7

K05548	benK	MFS transporter, AAHS family, benzoate transport protein	Membrane Transport	1	1
K05559	phaA	multicomponent K+:H+ antiporter subunit A	Cellular Processes and Signaling	1	1
K05786	rarD	chloramphenicol-sensitive protein RarD	Cellular Processes and Signaling	1	1
K05795	terD	tellurium resistance protein TerD	Cellular Processes and Signaling	1	1
K05807	comL	putative lipoprotein	Poorly Characterized	1	1
K05838	ybbN	putative thioredoxin	Folding, Sorting and Degradation	4	2
K05844	rimK	ribosomal protein S6 modification protein	Translation	7	2
K05989	E3.2.1.40	alpha-L-rhamnosidase [EC:3.2.1.40]	Metabolism	1	1
K06013	E3.4.24.84	STE24 endopeptidase [EC:3.4.24.84]	Enzyme Families	1	1
K06024	scpB	segregation and condensation protein B	Replication and Repair	1	1
K06041	E5.3.1.13	arabinose-5-phosphate isomerase [EC:5.3.1.13]	Cellular Processes and Signaling	7	3
K06048	ybdK	carboxylate-amine ligase [EC:6.3.-.-]	Metabolism	1	1
K06135	pqqA	pyrroloquinoline quinone biosynthesis protein A	Poorly Characterized	9	2
K06143	creD	inner membrane protein	Cellular Processes and Signaling	1	1
K06147	ABCB-BAC	ATP-binding cassette, subfamily B, bacterial	Membrane Transport	5	2
K06158	ABCF3	ATP-binding cassette, sub-family F, member 3	Genetic Information Processing	1	1
K06168	miaB	bifunctional enzyme involved in thiolation and methylation of tRNA	Genetic Information Processing	1	1
K06177	rluA	ribosomal large subunit pseudouridine synthase A [EC:5.4.99.12]	Translation	1	1
K06178	rluB	ribosomal large subunit pseudouridine synthase B [EC:5.4.99.12]	Translation	1	1
K06180	rluD	ribosomal large subunit pseudouridine synthase D [EC:5.4.99.12]	Translation	1	1
K06189	corC	magnesium and cobalt transporter	Cellular Processes and Signaling	1	1
K06192	pqiB	paraquat-inducible protein B	Poorly Characterized	2	1
K06204	dksA	DnaK suppressor protein	Translation - Transcription	3	2
K06214	csgG	curli production assembly/transport component CsgG	Membrane Transport	23	6
K06217	phoH, phoL	phosphate starvation-inducible protein PhoH and related proteins	Cellular Processes and Signaling	475	7
K06351	kipI	inhibitor of KinA	Cellular Processes and Signaling	1	1
K06400	spoIVCA	site-specific DNA recombinase	Cellular Processes and Signaling	6	3
K06405	spoVAC	stage V sporulation protein AC	Cellular Processes and Signaling	1	1
K06415	spoVR	stage V sporulation protein R	Cellular Processes and Signaling	20	4
K06416	spoVS	stage V sporulation protein S	Cellular Processes and Signaling	8	2
K06860	K06860		Poorly Characterized	1	1
K06872	K06872		Poorly Characterized	1	1
K06877	K06877		Poorly Characterized	2	1
K06879	queF	7-cyano-7-deazaguanine reductase [EC:1.7.1.13]	Poorly Characterized	49	4
K06881	K06881		Poorly Characterized	1	1
K06884	K06884		Poorly Characterized	11	1
K06885	K06885		Poorly Characterized	1	1
K06890	K06890		Poorly Characterized	9	3
K06891	clpS	ATP-dependent Clp protease adaptor protein ClpS	Genetic Information Processing	31	4
K06894	K06894		Poorly Characterized	1	1
K06901	pbuG	putative MFS transporter, AGZA family, xanthine/uracil permease	Membrane Transport	4	3
K06902	UMF1	MFS transporter, UMF1 family	Membrane Transport	1	1
K06903	K06903		Poorly Characterized	131	3
K06904	K06904		Poorly Characterized	496	6
K06905	K06905		Poorly Characterized	4	1
K06906	K06906		Poorly Characterized	4	1
K06907	K06907		Poorly Characterized	627	7
K06909	K06909		Poorly Characterized	1744	8
K06911	K06911		Poorly Characterized	1	1
K06915	K06915		Poorly Characterized	11	3
K06917	selU	tRNA 2-selenouridine synthase [EC:2.9.1.-]	Poorly Characterized	1	1
K06918	K06918		Poorly Characterized	1	1
K06919	K06919	putative DNA primase/helicase	Genetic Information Processing	716	8
K06920	queC	queuosine biosynthesis protein QueC	Poorly Characterized	396	8
K06927	K06927		Poorly Characterized	2	1
K06938	K06938		Poorly Characterized	5	3
K06940	K06940		Poorly Characterized	7	2
K06941	rlmN	ribosomal RNA large subunit methyltransferase N [EC:2.1.1.-]	Translation	2	1
K06950	K06950	uncharacterized protein	Poorly Characterized	1	1
K06952	K06952		Poorly Characterized	1	1
K06955	K06955		Poorly Characterized	1	1
K06960	K06960		Poorly Characterized	1	1
K06966	K06966		Poorly Characterized	2	2
K06969	rlmI	ribosomal RNA large subunit methyltransferase I [EC:2.1.1.-]	Translation	2	1
K06972	K06972		Poorly Characterized	1	1
K06980	K06980		Poorly Characterized	1	1
K06991	K06991		Poorly Characterized	1	1
K06994	K06994	putative drug exporter of the RND superfamily	Poorly Characterized	2	1
K06995	K06995		Poorly Characterized	3	2
K07000	K07000		Poorly Characterized	1	1
K07001	K07001		Poorly Characterized	1	1
K07003	K07003		Poorly Characterized	2	2
K07004	K07004		Poorly Characterized	1	1
K07008	DUG3	glutamine amidotransferase	Poorly Characterized	12	2
K07010	K07010	putative glutamine amidotransferase	Enzyme Families	4	2
K07011	K07011		Poorly Characterized	49	2
K07012	K07012		Poorly Characterized	2	1
K07019	K07019		Poorly Characterized	2	1
K07024	K07024		Poorly Characterized	1	1
K07031	K07031		Poorly Characterized	15	5
K07033	K07033		Poorly Characterized	1	1
K07043	K07043		Poorly Characterized	1	1
K07044	K07044		Poorly Characterized	1	1
K07052	K07052		Poorly Characterized	1	1
K07053	K07053		Poorly Characterized	1	1
K07056	K07056		Poorly Characterized	1	1
K07058	K07058	membrane protein	Poorly Characterized	2	1
K07067	disA	DNA integrity scanning protein	Poorly Characterized	1	1
K07071	K07071		Poorly Characterized	2	1
K07074	K07074		Poorly Characterized	1	1
K07088	K07088		Poorly Characterized	2	2
K07090	K07090		Poorly Characterized	1	1
K07093	K07093		Poorly Characterized	1	1
K07098	K07098		Poorly Characterized	1	1
K07099	K07099		Poorly Characterized	1	1
K07100	K07100		Poorly Characterized	1	1
K07101	K07101		Poorly Characterized	40	2
K07103	K07103		Poorly Characterized	1	1
K07107	ybgC	acyl-CoA thioester hydrolase [EC:3.1.2.-]	Poorly Characterized	1	1
K07114	K07114	uncharacterized protein	Poorly Characterized	2	2
K07117	K07117		Poorly Characterized	4	3
K07124	K07124		Poorly Characterized	1	1
K07126	K07126		Poorly Characterized	6	2
K07130	K07130		Poorly Characterized	1	1
K07137	K07137		Poorly Characterized	271	4
K07154	K07154		Poorly Characterized	3	2
K07156	pcoC		Poorly Characterized	1	1
K07168	K07168	CBS domain-containing membrane protein	Cellular Processes and Signaling	4	1
K07169	K07169	FHA domain-containing protein	Cellular Processes and Signaling	1	1
K07175	phoH2	PhoH-like ATPase	Cellular Processes and Signaling	186	6
K07180	prkA	serine protein kinase	Cellular Processes and Signaling	52	5
K07182	K07182	CBS domain-containing protein	Cellular Processes and Signaling	2	2
K07219	K07219	putative molybdopterin biosynthesis protein	Cellular Processes and Signaling	3	1
K07228	K07228	TrkA domain protein	Cellular Processes and Signaling	1	1
K07233	copB	copper resistance protein B	Cellular Processes and Signaling	1	1
K07239	TC.HME	heavy-metal exporter, HME family	Cellular Processes and Signaling	7	2

K07243	FTR1	high-affinity iron transporter	Cellular Processes and Signaling	1	1
K07261	mepA	penicillin-insensitive murein endopeptidase [EC:3.4.24.-]	Enzyme Families	1	1
K07262	pbpG	D-alanyl-D-alanine endopeptidase (penicillin-binding protein 7) [EC:3.4.99.-]	Enzyme Families	25	2
K07263	pqqL	zinc protease [EC:3.4.99.-]	Enzyme Families	4	1
K07270	K07270	glycosyl transferase, family 25	Cellular Processes and Signaling	21	3
K07272	rgpF	rhamnosyltransferase [EC:2.4.1.-]	Glycan Biosynthesis and Metabolism	1	1
K07273	acm	lysozyme	Cellular Processes and Signaling	2	1
K07288	tspA	uncharacterized membrane protein	Cellular Processes and Signaling	1	1
K07303	E1.3.99.16B	isoquinoline 1-oxidoreductase, beta subunit [EC:1.3.99.16]	Metabolism	1	1
K07304	msrA	peptide-methionine (S)-S-oxide reductase [EC:1.8.4.11]	Genetic Information Processing	1	1
K07316	mod	adenine-specific DNA-methyltransferase [EC:2.1.1.72]	Genetic Information Processing	3	2
K07317	K07317	adenine-specific DNA-methyltransferase [EC:2.1.1.72]	Genetic Information Processing	1	1
K07319	yhdJ	putative adenine-specific DNA-methyltransferase [EC:2.1.1.72]	Genetic Information Processing	780	9
K07335	bmpA, bmpB, tmpC	basic membrane protein A and related proteins	Cellular Processes and Signaling	1	1
K07336	K07336	PKHD-type hydroxylase [EC:1.14.11.-]	Metabolism	34	6
K07357	fimB	type 1 fimbriae regulatory protein FimB	Genetic Information Processing	3	2
K07358	fimE	type 1 fimbriae regulatory protein FimE	Genetic Information Processing	2	1
K07386	pepO	putative endopeptidase [EC:3.4.24.-]	Enzyme Families	1	1
K07389	cyaC, hlyC, rtxC	cytolysin-activating lysine-acyltransferase [EC:2.3.1.-]	Genetic Information Processing	5	1
K07391	comM	magnesium chelatase family protein	Genetic Information Processing	1	1
K07393	ECM4	putative glutathione S-transferase	Genetic Information Processing	1	1
K07394	K07394	SM-20-related protein	Genetic Information Processing	8	1
K07396	K07396	putative protein-disulfide isomerase	Genetic Information Processing	1	1
K07442	TRM61, GCD14	tRNA (adenine-N1-)-methyltransferase catalytic subunit [EC:2.1.1.36]	Genetic Information Processing	1	1
K07444	ypsC	putative N6-adenine-specific DNA methylase [EC:2.1.1.-]	Genetic Information Processing	1	1
K07452	K07452, mcrB	5-methylcytosine-specific restriction enzyme B [EC:3.1.21.-]	Genetic Information Processing	1	1
K07455	recT	recombination protein RecT	Replication and Repair	90	4
K07460	yraN	putative endonuclease	Genetic Information Processing	1	1
K07461	K07461	putative endonuclease	Genetic Information Processing	7	3
K07465	K07465	putative RecB family exonuclease	Genetic Information Processing	3	2
K07474	xtmA	phage terminase small subunit	Genetic Information Processing	24	5
K07478	ycaJ	putative ATPase	Genetic Information Processing	34	4
K07491	K07491	putative transposase	Genetic Information Processing	7	3
K07496	K07496	putative transposase	Genetic Information Processing	108	8
K07497	K07497	putative transposase	Genetic Information Processing	2	2
K07501	K07501	hypothetical protein	Genetic Information Processing	59	5
K07505	K07505	hypothetical protein	Genetic Information Processing	50	2
K07507	mgtC	putative Mg2+ transporter-C (MgtC) family protein	Cellular Processes and Signaling	2	2
K07552	bcr	MFS transporter, DHA1 family, bicyclomycin/chloramphenicol resistance protein	Membrane Transport	3	2
K07566	SUA5	putative translation factor	Translation	1	1
K07568	queA	S-adenosylmethionine:tRNA ribosyltransferase-isomerase [EC:5.-.-.-]	Genetic Information Processing	1	1
K07576	K07576	metallo-beta-lactamase family protein	Genetic Information Processing	1	1
K07589	folX	D-erythro-7,8-dihydroneopterin triphosphate epimerase [EC:5.-.-.-]	Metabolism	1	1
K07726	K07726	putative transcriptional regulator	Transcription	1	1
K07728	K07728	putative transcriptional regulator	Transcription	1	1
K07729	K07729	putative transcriptional regulator	Transcription	4	1
K07738	nrdR	transcriptional repressor NrdR	Transcription	3	1
K07741	K07741	anti-repressor protein	Genetic Information Processing	11	3
K07749	frc	formyl-CoA transferase [EC:2.8.3.16]	Metabolism	1	1
K08151	tetA	MFS transporter, DHA1 family, tetracycline resistance protein	Membrane Transport	1	1
K08160	cmr, mdfA	MFS transporter, DHA1 family, multidrug/chloramphenicol efflux transport protein	Membrane Transport	1	1
K08223	fsr	MFS transporter, FSR family, fosmidomycin resistance protein	Membrane Transport	1	1
K08259	lytM	lysostaphin [EC:3.4.24.75]	Enzyme Families	1	1
K08282	E2.7.11.1	non-specific serine/threonine protein kinase [EC:2.7.11.1]	Cellular Processes and Signaling	2	2
K08304	mltA	membrane-bound lytic murein transglycosylase A [EC:3.2.1.-]	Metabolism	1	1
K08307	mltD, dniR	membrane-bound lytic murein transglycosylase D [EC:3.2.1.-]	Metabolism	23	2
K08309	slt	soluble lytic murein transglycosylase [EC:3.2.1.-]	Metabolism	12	2
K08482	kaiC	circadian clock protein KaiC	Cellular Processes and Signaling	28	2
K08602	pepF, pepB	oligoendopeptidase F [EC:3.4.24.-]	Enzyme Families	1	1
K08884	K08884	serine/threonine protein kinase, bacterial [EC:2.7.11.1]	Enzyme Families	6	3
K08930	pucA	light-harvesting protein B-800-850 alpha chain	Energy Metabolism	1	1
K08981	K08981	putative membrane protein	Poorly Characterized	2	1
K08999	K08999	hypothetical protein	Poorly Characterized	1	1
K09005	K09005	hypothetical protein	Poorly Characterized	15	4
K09007	K09007	hypothetical protein	Poorly Characterized	12	5
K09013	sufC	Fe-S cluster assembly ATP-binding protein	Membrane Transport	3	2
K09014	sufB	Fe-S cluster assembly protein SufB	Poorly Characterized	13	3
K09117	K09117	hypothetical protein	Poorly Characterized	1	1
K09125	K09125	hypothetical protein	Poorly Characterized	4	3
K09129	K09129	hypothetical protein	Poorly Characterized	1	1
K09134	K09134	hypothetical protein	Poorly Characterized	6	1
K09136	ycaO	ribosomal protein S12 methylthiotransferase	Translation	1	1
K09139	K09139	hypothetical protein	Poorly Characterized	1	1
K09181	yfiQ	hypothetical protein	Poorly Characterized	1	1
K09607	ina	immune inhibitor A [EC:3.4.24.-]	Enzyme Families	1	1
K09728	K09728	hypothetical protein	Poorly Characterized	1	1
K09744	K09744	hypothetical protein	Poorly Characterized	8	1
K09746	K09746	hypothetical protein	Poorly Characterized	2	1
K09760	rmuC	DNA recombination protein RmuC	Genetic Information Processing	1	1
K09774	lptA	lipopolysaccharide export system protein LptA	Cellular Processes and Signaling	1	1
K09776	K09776	hypothetical protein	Poorly Characterized	10	3
K09786	K09786	hypothetical protein	Poorly Characterized	20	3
K09787	K09787	hypothetical protein	Poorly Characterized	1	1
K09790	K09790	hypothetical protein	Poorly Characterized	4	2
K09791	K09791	hypothetical protein	Poorly Characterized	1	1
K09818	ABC.MN.S	manganese/iron transport system substrate-binding protein	Membrane Transport	1	1
K09861	K09861	hypothetical protein	Poorly Characterized	2	1
K09892	K09892	hypothetical protein	Poorly Characterized	3	1
K09935	K09935	hypothetical protein	Poorly Characterized	3	1
K09946	K09946	hypothetical protein	Poorly Characterized	1	1
K09955	K09955	hypothetical protein	Poorly Characterized	2	1
K09960	K09960	hypothetical protein	Poorly Characterized	54	4
K09961	K09961	hypothetical protein	Poorly Characterized	26	2
K09966	K09966	hypothetical protein	Poorly Characterized	5	2
K09968	K09968	hypothetical protein	Poorly Characterized	2	2
K09973	K09973	hypothetical protein	Poorly Characterized	1	1
K10026	queE, ykvL, ygcF	queuosine biosynthesis protein QueE	Poorly Characterized	125	5
K10704	UBE2V	ubiquitin-conjugating enzyme E2 variant	Replication and Repair	1	1
K10726	mcm, cdc21	replicative DNA helicase Mcm [EC:3.6.4.-]	Replication and Repair	22	3
K10819	E2.7.13.3	histidine kinase	Metabolism	1	1
K10896	FANCM	fanconi anemia group M protein [EC:3.6.4.13]	Replication and Repair	2	1
K10906	recE	exodeoxyribonuclease VIII [EC:3.1.11.-]	Replication and Repair	33	2
K10908	POLRMT, RPO41	DNA-directed RNA polymerase, mitochondrial [EC:2.7.7.6]	Genetic Information Processing	36	4
K11068	hlyIII	hemolysin III	Signaling Molecules and Interaction	5	2
K11107	yfaE	ferredoxin	Metabolism	9	4
K11159	K11159	carotenoid cleavage dioxygenase	Metabolism	1	1
K11527	K11527	two-component system, unclassified family, sensor histidine kinase and response regulator [EC:2.7.13.3]	Signal Transduction - Enzyme Families	2	1
K11895	impH, vasB	type VI secretion system protein ImpH	Membrane Transport	2	1
K11900	impC	type VI secretion system protein ImpC	Membrane Transport	1	1
K12065	traB	conjugal transfer pilus assembly protein TraB	Membrane Transport	1	1
K12287	mshQ	MSHA biogenesis protein MshQ	Membrane Transport	1	1
K12507	fadK	acyl-CoA synthetase [EC:6.2.1.-]	Lipid Metabolism	1	1

K12684	K12684, sigA, sepA, esp	serine protease autotransporter [EC:3.4.21.-]	Membrane Transport	3	1
K12685	ssp	subtilase-type serine protease [EC:3.4.21.-]	Membrane Transport	1	1
K12950	ctpC	cation-transporting P-type ATPase C [EC:3.6.3.-]	Metabolism	1	1
K12979	lpxO	beta-hydroxylase [EC:1.14.11.-]	Glycan Biosynthesis and Metabolism	2	2
K12989	lpcC	mannosyltransferase [EC:2.4.1.-]	Glycan Biosynthesis and Metabolism	1	1
K12997	rgpB	rhamnosyltransferase [EC:2.4.1.-]	Glycan Biosynthesis and Metabolism	1	1
K13005	rfbV	abequosyltransferase [EC:2.4.1.-]	Glycan Biosynthesis and Metabolism	1	1
K13010	per	perosamine synthetase	Glycan Biosynthesis and Metabolism - Amino Acid Metabolism	14	4
K13013	wbqV	O-antigen biosynthesis protein WbqV	Glycan Biosynthesis and Metabolism	4	3
K13016	wbpB	UDP-D-GlcNAcA oxidase [EC:1.1.1.-]	Glycan Biosynthesis and Metabolism	8	2
K13018	wbpD	UDP-D-GlcNAc3NA acetyltransferase [EC:2.3.1.-]	Glycan Biosynthesis and Metabolism	1	1
K13019	wbpI	UDP-GlcNAc3NAcA epimerase [EC:5.1.3.23]	Glycan Biosynthesis and Metabolism	6	3
K13020	wlbA, bplA	UDP-D-GlcNAcA oxidase [EC:1.1.1.-]	Glycan Biosynthesis and Metabolism	2	2
K13057	treT	trehalose synthase [EC:2.4.1.245]	Metabolism	3	2
K13275	isp	major intracellular serine protease [EC:3.4.21.-]	Folding, Sorting and Degradation - Enzyme Families	1	1
K13281	uvsE, UVE1	UV DNA damage endonuclease [EC:3.-.-.-]	Genetic Information Processing	103	5
K13611	pksJ	polyketide synthase PksJ	Lipid Metabolism	11	1
K13628	iscA	iron-sulfur cluster assembly protein	Metabolism	54	3
K13635	cbl	LysR family transcriptional regulator, cys regulon transcriptional activator	Transcription	1	1
K13678	cpoA	monoglucosyldiacylglycerol glycosyltransferase [EC:2.4.1.-]	Glycan Biosynthesis and Metabolism	5	2
K13693	K13693	glucosyl-3-phosphoglycerate synthase [EC:2.4.1.-]	Glycan Biosynthesis and Metabolism	1	1
K13694	spr	lipoprotein Spr	Enzyme Families	7	2
K13695	nlpC	probable lipoprotein NlpC	Enzyme Families	8	2
K13819	K13819	NifU-like protein	Metabolism	2	1
K13930	mdcB	triphosphoribosyl-dephospho-CoA synthase [EC:2.7.8.25]	Metabolism	1	1
K14059	int	integrase	Genetic Information Processing	17	1
K14060	pinR	putative DNA-invertase from lambdoid prophage Rac	Genetic Information Processing	1	1
K14162	dnaE2	error-prone DNA polymerase [EC:2.7.7.7]	Replication and Repair	3	2
K14266	prnA	FADH2 O2-dependent halogenase I [EC:1.14.14.7]	Metabolism	25	4
K14287	ybdL	methionine aminotransferase [EC:2.6.1.-]	Amino Acid Metabolism	1	1
K14393	actP	cation/acetate symporter	Cellular Processes and Signaling	1	1
K14415	rtcB	protein RtcB	Poorly Characterized	1	1
K14441	rimO	ribosomal protein S12 methylthiotransferase [EC:2.-.-.-]	Translation	1	1
K14623	dinD	DNA-damage-inducible protein D	Poorly Characterized	42	1
K14645	K14645	serine protease [EC:3.4.21.-]	Folding, Sorting and Degradation - Enzyme Families	3	1
K14660	nodE	nodulation protein E [EC:2.3.1.-]	Cellular Processes and Signaling	1	1
K14665	amhX	amidohydrolase [EC:3.5.1.-]	Enzyme Families	1	1
K14680	E6.5.1.3	RNA ligase [EC:6.5.1.3]	Metabolism	72	3
K14744	rzpD	putative Rz endopeptidase from lambdoid prophage DLP12 [EC:3.4.-.-]	Poorly Characterized	8	2
